# Social safety nets, women’s economic achievements and agency in 45 countries: a systematic review and meta-analysis

**DOI:** 10.1038/s41562-025-02394-0

**Published:** 2026-02-05

**Authors:** Amber Peterman, Jingying Wang, Kevin Kamto Sonke, Janina Isabel Steinert

**Affiliations:** 1 Department of Public Policy, University of North Carolina at Chapel Hill, Chapel Hill, NC USA; 2 Evaluation Office, United Nations Children’s Fund, Nairobi, Kenya; 3 Department of Sociology, University of Zurich, Zurich, Switzerland; 4 Sanford School of Public Policy, Duke University, Durham, NC USA; 5 TUM School of Social Sciences and Technology, Technical University of Munich, Munich, Germany; 6 Munich Center for Health Economics and Policy, Munich, Germany

**Keywords:** Social policy, Economics

## Abstract

There are increasing calls for economic assistance in the form of social safety nets (SSNs) to be designed and implemented to promote women’s economic inclusion and agency, contributing to closing gender disparities globally. Here we investigate the extent to which SSNs affect women’s economic achievements and agency through a systematic review and meta-analysis of randomized controlled trials implemented in low- and middle-income countries. We searched six databases utilizing search strings in English, French and Spanish through December 2024. Studies were assessed for risk of bias using an adapted version of the Joanna Briggs Institute critical appraisal tool. Our sample includes 1,307 effect sizes from 93 studies, representing 218,828 women across 45 low- and middle-income countries. Using robust variance estimation meta-analysis, we show significant overall pooled effects (Hedges’ *g* = 0.107, *P* < 0.001, 95% confidence interval (CI) 0.085–0.129), driven by increases in economic achievements (productive work, savings, assets and expenditures) and agency (voice, autonomy and decision-making). We find significant treatment effects for unconditional cash transfers (Hedges’ *g* = 0.128, *P* < 0.001, 95% CI 0.097 to 0.159), social care services (Hedges’ *g* = 0.122, *P* < 0.001, 95% CI 0.071 to 0.174), asset transfers (Hedges’ *g* = 0.115, *P* < 0.001, 95% CI 0.071 to 0.160) and public work programmes (Hedges’ *g* = 0.127, *P* = 0.031, 95% CI 0.015 to 0.239). We find comparatively smaller effects for conditional cash transfers (Hedges’ *g* = 0.059, *P* = 0.019, 95% CI 0.011 to 0.108) and found no evidence of effects for in-kind transfers. SSNs can empower women economically and socially; however, limitations and evidence gaps remain, including the need for further rigorous testing of design and operational components, the role of contextual factors and cost–benefit analysis with a gender lens.

## Abstract (web summary)

This meta-analysis of randomized controlled trials indicates that social safety nets can improve women’s economic achievements and agency. Pooled treatment effects are largest for unconditional cash transfers, public work programmes, social care services and asset transfers.

## Main

Social safety nets (SSNs), or economic assistance including cash, in-kind and asset transfers, are widely used policy instruments to promote household economic security, resiliency to shocks and investment in human capital^1,2^. Alongside their ability to cost-effectively reach and promote dignity and inclusion among marginalized populations, an increasingly recognized advantage of SSNs is their ability to address drivers of gender inequality^3,4^. Advocates for a gender-sensitive approach have argued for provisions at the systems, policy and programme levels that consider and seek to close gender disparities across a wide range of outcomes^5–7^. Evidence supports these efforts, showing that carefully designed SSNs can have multifaceted positive effects for women, including transforming their economic status via participation in the labour force, increasing income and building wealth stores, as well as increasing their sense of agency via participation in household and community decision-making^1,3,8,9^. Nonetheless, governments have struggled to put these recommendations into practice. For example, among 3,099 global government social protection and labour market responses that were planned, adapted or implemented through 2021 in response to the coronavirus disease 2019 (COVID-19) pandemic, less than 20% took gender into account (defined as either targeting women’s economic security or supporting unpaid care)^6^. As investment in SSNs increasingly responds to global shocks, including conflict and climate change, better understanding of their potential to promote women’s economic and social empowerment is needed.

Do SSNs increase women’s economic achievements and promote their agency within households and communities? Previous reviews suggest they can^1,3,8,9^. However, so far, reviews have been largely narrative, lacking meta-analytic methodology able to aggregate evidence across SSN typologies, geographies and outcomes. Furthermore, studies captured in previous reviews are dominated by cash transfers, rather than broader modalities. In addition, multiple reviews set out to aggregate evidence on the role of design and implementation components in delivering impacts for women; however, many conclude the evidence base is too mixed or too thin to make firm conclusions^1,3^. Finally, some studies point to potential adverse effects or unintended consequences of SSNs for women. A common example raised is the potential for cash transfers with conditions to increase women’s burden of unpaid care, reinforcing her involvement in childcare or domestic work^10,11^. This might occur if women are designated as responsible for attending mandatory trainings associated with the intervention or for monitoring children’s schooling or health due to coresponsibilities. It is also possible that interventions that appear ‘successful’ in terms of household-level impacts could simultaneously result in no or adverse effects for women specifically, or increased inequality across household members, warranting further examination whether these trends hold across multiple studies.

The objective of this study is to synthesize evidence on the effectiveness of SSNs on women’s economic achievements and agency in low- and middle-income countries (LMICs). We define SSNs broadly following the World Bank’s Atlas of Social Protection Indicators of Resilience and Equity (ASPIRE) non-contributory programming to include seven instruments: (1) unconditional cash transfers (UCTs), (2) conditional cash transfers (CCTs), (3) food, vouchers or consumable in-kind transfers, (4) productive asset transfers, (5) public work programmes, (6) fee waivers and subsidies and (7) social care services^12^. We include SSN with complementary programming (bundled), as well as stand-alone economic benefits programmes. Using a systematic review and meta-analysis of experimental evaluations, we answer the following questions: (1) What is the direction and magnitude of impact of SSNs on women’s economic achievements and agency?; (2) How do these impacts vary according to SSN modality and outcome measure, as well as enablers and barriers related to intervention and evaluation designs, target group and context?; and (3) What are the cost–benefit calculations accompanying these interventions? We define economic achievements and agency broadly to capture multidimensional aspects of each concept and provide a comprehensive view of evidence to inform future investment and policy.

We aggregate results from 1,307 effect sizes found in 115 publications, representing 93 randomized controlled trials (RCTs) and 218,828 women across 45 LMICs. Literature is relatively recent, with over half of included papers published in the past 5 years (2019 or after)—indicating a quickly emerging and dynamic field. We find SSNs improve women’s economic achievements (Hedges’ *g* = 0.113, degrees of freedom (d.f.) 72.3, *P* < 0.001, 95% confidence interval (CI) 0.085–0.142, *n* = 843) and agency (Hedges’ *g* = 0.101, d.f. 55.3, *P* < 0.001, 95% CI 0.063 to 0.139, *n* = 462). Pooled effects show that impacts from UCTs (Hedges’ *g* = 0.128, d.f. 54.7, *P* < 0.001, 95% CI 0.097 to 0.159, *n* = 817), social care (Hedges’ *g* = 0.122, d.f. 8.98, *P* < 0.001, 95% CI 0.071 to 0.174, *n* = 105) and asset transfers (Hedges’ *g* = 0.115, d.f. 14.7, *P* < 0.001, 95% CI 0.071 to 0.160, *n* = 216) are highly significant, while public works programmes are comparative in magnitude, but of weaker statistical significance, probably in part due to lower power (Hedges’ *g* = 0.127, d.f. 7.67, *P* = 0.031, 95% CI 0.015 to 0.239, *n* = 106). Impacts from CCTs are comparatively smaller (Hedges’ *g* = 0.059, d.f. 18.6, *P* = 0.019, 95% CI 0.011 to 0.108, *n* = 167) and associated with smaller effect sizes in meta-regressions. Finally, there is no evidence of a significant impact for food, vouchers and in-kind transfers (Hedges’ *g* = 0.071, d.f. 5.97, *P* = 0.123, 95% CI −0.026 to 0.169, *n* = 112). Pooled effects for the domain of economic achievement show strong effects for assets (Hedges’ *g* = 0.235, d.f. 14.9, *P* < 0.001, 95% CI 0.125 to 0.345, *n* = 83), savings (Hedges’ *g* = 0.229, d.f. 19.9, *P* < 0.001, 95% CI 0.147 to 0.311, *n* = 75), expenditures (Hedges’ *g* = 0.177, d.f. 17.9, *P* = 0.002, 95% CI 0.072 to 0.282, *n* = 55), labour force participation (Hedges’ *g* = 0.106, d.f. 42.6, *P* < 0.001, 95% CI 0.058 to 0.154, *n* = 155) and productive work intensity (for example, hours worked or earnings) (Hedges’ *g* = 0.075, d.f. 48.9, *P* < 0.001, 95% CI 0.044 to 0.106, *n* = 370). Meanwhile, impacts for debt and loans (Hedges’ *g* = 0.105, d.f. 7.98, *P* = 0.233, 95% CI −0.082 to 0.291, *n* = 21) and care work intensity (Hedges’ *g* = -0.015, d.f. 14.2, *P* = 0.467, 95% CI −0.058 to 0.028, *n* = 62) are insignificant and care work participation has insufficient power to estimate effects. Pooled effects for the domain of agency show that voice (Hedges’ *g* = 0.172, d.f. 11.0, *P* = 0.011, 95% CI 0.048 to 0.297, *n* = 49), autonomy (Hedges’ *g* = 0.105, d.f. 32.1, *P* = 0.001, 95% CI 0.046 to 0.165, *n* = 152), and decision-making (Hedges’ *g* = 0.087, d.f. 37.5, *P* = 0.001, 95% CI 0.036 to 0.137, *n* = 238) are robust and significant, while aspirations and leadership have insufficient power to estimate effects. Apart from the association with conditionalities, meta-regressions investigating study, intervention and outcome-level factors show few significant predictors of higher (lower) effects. We hypothesize that low power and high heterogeneity between studies may have contributed to these minimal results.

Our study builds on two bodies of evidence. The first seeks to understand which interventions are most promising to build women’s agency and economic achievements. Our results align with a narrative review of 160 experimental and quasi-experimental evaluations from LMICs, aimed at identifying what works to enhance women’s agency (including measures of economic, social and political standing)^13^. Among 16 different intervention types examined, 4 were selected as having ‘strong or moderately strong’ evidence across multiple forms of agency, including cash and in-kind transfers and the graduation approach, both of which fall under our definition of SSNs. Our review also builds on meta-analyses examining effects of economic interventions more broadly on women’s agency or economic standing. Results from previous meta-analyses show that our pooled effects are comparable to those found for vocational training interventions and their impacts on women’s employment and earnings are lower than those found for economic self-help groups on empowerment outcomes and are consistently higher than those found for microcredit and savings interventions and their impacts on a range of similar economic and agency outcomes^14–18^. The second strand of literature aims to unpack under what circumstances SSNs can improve women’s outcomes and how they can be better designed and implemented to leverage gender equality impacts. Previous narrative reviews and practice-based recommendations have suggested that targeting women, ensuring a benefits are of sufficient value, incorporating key complementary programming and attention to gender in operations and implementation matters^1,3,8,9^. However, like our study, pervious reviews suggest that, for many design and contextual factors, more research is needed to make firm conclusions. Our study shows that impacts of SSN on women’s economic achievement and agency are promising, but many gaps remain.

## Results

### Sample

**Fig. 1:**
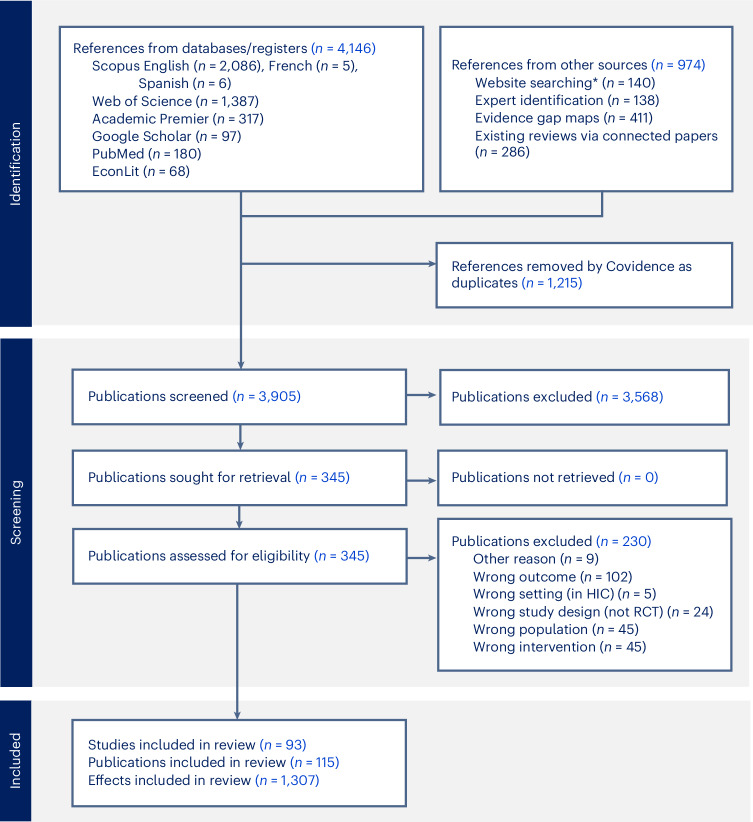
PRISMA flow diagram. HIC, high income country; RCT, randomised controlled trials. *Websites searched included the World Bank open knowledge repository, World Bank Gender Innovation Labs, Asian Development Bank, Inter-American Development Bank, African Development Bank, J-PAL, Innovations for Poverty Action, UNICEF Innocenti, Oxford Policy Management, the International Food Policy Research Institute, Socialprotection.org and the Transfer Project.

Figure 1 presents the Preferred Reporting Items for Systematic Reviews and Meta-Analyses (PRISMA) flow diagram for the study (see ‘Sample construction’ section in the Methods for a detailed description). The final sample for the analysis originated from 5,120 paper hits (4,146 from databases and 974 from other sources) and includes 1,307 effect sizes from 115 papers, representing 93 RCTs and 218,828 women. Supplementary Fig. 1 shows the evolution of papers across years by publication type (top panel) and by region (bottom panel).

**Fig. 2:**
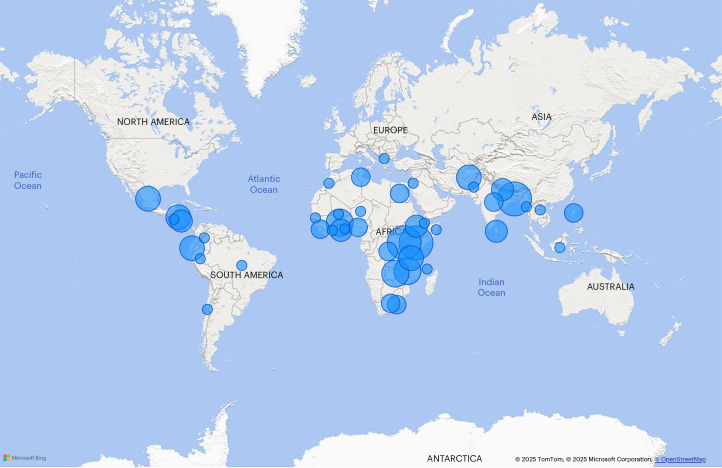
Geographical distribution of overall effect sizes. Distribution of effects (*n* = 1,307) across 45 countries; including 57% of all impacts in sub-Saharan Africa, 21% in South Asia, 11% in Latin America and the Caribbean, 9% in the Middle East and North Africa, 2% in East Asia and the Pacific and less than 1% in Europe and Central Asia.

Table 1 reports sample descriptives for included studies at the publication level (*n* = 115), the intervention arm level (*n* = 135) and the effect level (*n* = 1,307), overall and by type of SSN (note that percentages across SSN types do not sum to 100%, as some publications evaluate interventions with multiple treatment arms studying different types of SSN, or layer multiple SSNs within an intervention package). The majority of publications examine UCTs (*n* = 65 or 57%) and CCTs (*n* = 24 or 21%), with fewer publications evaluating asset transfers (*n* = 17 or 15%), social care (*n* = 9 or 8%), public works programmes and food, vouchers or in-kind transfer programmes (*n* = 9 and 7, respectively). Publications cover a total of 45 countries, with effects concentrated in sub-Saharan Africa (57%), South Asia (21%) and Latin America and the Caribbean (11%) (Fig. 2). Panel A of Table 1 shows that most publications focus on lower-middle income settings (52%), followed by low income (33%) and upper-middle income (15%) settings. A minority of studies take place in fragile settings (22%) or urban settings, with overall approximately a third of studies including at least some or all the sample in urban areas.

**Table 1:** Sample characteristics

	All	UCTs	CCTs	Food, voucher or in-kind	Asset transfer	Public works	Social care
**Panel A: publication level**							
*N*	115	65	24	7	17	9	9
Year of publication							
Before 2015	0.12	0.11	0.25	0.00	0.12	0.11	0.00
Between 2015 and 2019	0.38	0.40	0.50	0.57	0.65	0.11	0.22
After 2019	0.50	0.49	0.25	0.43	0.24	0.78	0.78
Region							
Sub-Saharan Africa	0.52	0.62	0.42	0.14	0.29	0.22	0.44
South Asia	0.19	0.29	0.04	0.14	0.53	0.11	0.11
Latin America and the Caribbean	0.17	0.06	0.42	0.57	0.12	0.11	0.33
Middle East and North Africa	0.06	0.02	0.04	0.14	0.00	0.33	0.11
East Asia and Pacific	0.04	0.02	0.08	0.00	0.06	0.11	0.00
Europe and Central Asia	0.01	0.00	0.00	0.00	0.00	0.11	0.00
Income group							
Low income	0.33	0.42	0.13	0.14	0.29	0.11	0.33
Lower-middle income	0.52	0.52	0.58	0.43	0.65	0.56	0.44
Upper-middle income	0.15	0.06	0.29	0.43	0.06	0.33	0.22
Fragile setting (any)	0.22	0.22	0.25	0.57	0.12	0.33	0.00
Urban setting (any)	0.30	0.23	0.38	0.43	0.12	0.33	0.67
**Panel B: intervention arm level**							
*N*	135	75	25	9	19	10	12
Implementer							
Government	0.38	0.37	0.48	0.11	0.16	0.50	0.42
NGO, UN or other	0.39	0.40	0.36	0.67	0.63	0.20	0.17
Researchers	0.23	0.23	0.16	0.22	0.21	0.30	0.42
Scale of implementation							
Pilot	0.33	0.29	0.40	0.33	0.26	0.50	0.17
Mid-level	0.47	0.48	0.48	0.44	0.32	0.30	0.58
At scale	0.21	0.23	0.12	0.22	0.42	0.20	0.25
Value of economic benefit							
Total value (US$)	474	533	274	400	711	631	387
First tercile	0.39	0.39	0.40	0.22	0.32	0.40	0.50
Second tercile	0.34	0.27	0.52	0.56	0.16	0.20	0.42
Third tercile	0.27	0.35	0.08	0.22	0.53	0.40	0.08
Targeting							
Poverty targeting	0.68	0.73	0.76	0.56	0.79	0.50	0.42
Gender targeting	0.75	0.72	0.84	0.67	0.47	0.60	1.00
Plus components							
Gender-neutral	0.21	0.20	0.28	0.22	0.42	0.00	0.33
Gender-sensitive	0.37	0.41	0.44	0.44	0.42	0.20	0.08
Economic	0.13	0.17	0.16	0.00	0.26	0.00	0.08
Health or protection	0.09	0.09	0.08	0.00	0.21	0.00	0.17
Training or information	0.44	0.43	0.60	0.67	0.53	0.20	0.17
Other	0.10	0.15	0.00	0.11	0.32	0.00	0.17
Female sample age							
Age: ≤24 years	0.16	0.12	0.32	0.11	0.05	0.20	0.08
Age: 25–39 years	0.68	0.69	0.48	0.89	0.74	0.40	0.92
Age: 40+ years	0.13	0.15	0.20	0.00	0.16	0.30	0.00
**Panel C: effect level**	All	UCTs	CCTs	Food, voucher or in-kind	Asset transfer	Public works	Social care
*N*	1,307	817	167	112	216	106	91
Domain: economic	0.64	0.62	0.68	0.25	0.53	0.79	0.92
Labour force participation	0.12	0.07	0.16	0.09	0.06	0.41	0.16
Productive work intensity	0.28	0.27	0.39	0.09	0.20	0.24	0.49
Care work participation	0.01	0.00	0.00	0.00	0.00	0.00	0.11
Care work intensity	0.05	0.04	0.02	0.01	0.00	0.13	0.13
Savings	0.06	0.08	0.03	0.01	0.06	0.00	0.01
Debt or loans	0.02	0.02	0.02	0.01	0.01	0.00	0.00
Assets	0.06	0.08	0.02	0.01	0.17	0.00	0.00
Expenditure	0.04	0.05	0.04	0.02	0.01	0.02	0.01
Aggregate economic	0.01	0.01	0.00	0.02	0.02	0.00	0.00
Domain: agency	0.35	0.38	0.32	0.75	0.47	0.21	0.08
Decision-making	0.18	0.20	0.05	0.38	0.35	0.08	0.03
Autonomy and self-efficacy	0.12	0.12	0.11	0.29	0.05	0.08	0.04
Aspirations and goals	0.00	0.00	0.00	0.00	0.00	0.04	0.00
Voice	0.04	0.03	0.14	0.06	0.06	0.00	0.00
Leadership	0.00	0.00	0.00	0.00	0.00	0.00	0.00
Aggregate agency	0.01	0.02	0.01	0.02	0.00	0.00	0.00
Duration of intervention							
12+ months	0.56	0.69	0.43	0.46	0.68	0.04	0.58
Time post-intervention at survey							
12+ months	0.50	0.55	0.46	0.43	0.85	0.09	0.22

Sample sizes across SSN types do not sum to the full sample, as interventions may include more than one SSN. CCT, conditional cash transfer; UCT, unconditional cash transfer.

Table 1 (panel B) summarizes characteristics of study intervention arms (*n* = 135). Overall, governments were the primary implementers (38%), followed by non-governmental organizations (NGOs) or United Nations (UN) agencies (39%) and research teams (23%). Approximately half of intervention arms were assessed as mid-level in scale, while the remaining were split between pilot (33%) and at-scale implementation (21%). The average total value of economic benefits over implementation periods studied is US$474, which was relatively larger in asset transfer and public works arms (just over US$711 and US$631, respectively) and relatively smaller in CCT arms and social care arms (US$274 and US$387, respectively). Most intervention arms were poverty targeted in some way (68%), as well as gender targeted (75%), including targeting adolescent girls or women as mothers, caregivers, entrepreneurs or female-headed households. Most interventions also have some form of plus component (59%, 21% with a gender-neutral plus component and 37% with a gender sensitive plus component) with asset transfers and CCTs showing greater likelihood of complementary programming (84% and 72%, respectively), while public work programmes and care services show lower likelihood of the same (20% and 42%, respectively). Plus components were the most likely to be training or informational (44% of all interventions), or include other types of economic (13%), health or protection (9%) or other programming (10%). Most women in the study sample were in the reproductive ages, with only 16% of average samples being younger than 24 years old (youth) and 13% of average samples being over the age of 40 years.

We aimed to categorize holistic measures of women’s economic achievement and agency following existing frameworks, excluding measures that were not entirely woman specific. For each category and domain, aggregate indicators or indices were also considered if they were woman specific and included a majority of qualifying indicators. Effects are split 64% (35%) across economic achievement (agency). Within the economic achievement domain, the most common indicators are productive work intensity (28% of all effects), followed by labour force participation (12%), assets (6%) and savings (6%). Within the agency domain, the most common indicators are decision-making (18% of all effects), followed by autonomy and self-efficacy (12%) and voice (4%). Very few studies measure care work participation, debt or loans, aspirations and goals, or leadership. Further details detailing outcome indicator definitions and parameters are included in the Methods and Supplementary Table 1. The average effect relates to interventions that last 12 months (range 1–60 months), with 56% of interventions lasting 12 months or longer. The average effect relates to follow-up measures taken approximately 14 months after interventions ended (range 0–140, or nearly 12 years), with 50% of effects being collected 12 months or longer post-intervention (see Supplementary Fig. 2 for a visual distribution of intervention and follow-up timing). Supplementary Table 2 gives further details of each publication, including authors, year of publication, country of study, intervention type and name, number of aggregate impacts included in the meta-analysis (total and by domain) and overall quality assessment score.

### Pooled effects

**Fig. 3:**
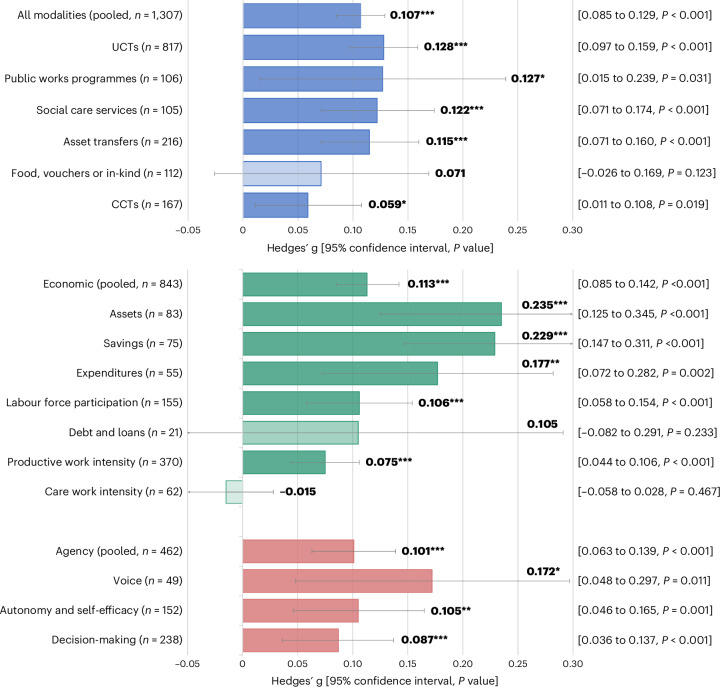
Pooled effects by SSN type (top) and outcome indicator (bottom). Average pooled effect sizes are calculated using standardized impacts from RVE and reported with 95% CIs (error bars). All statistical tests reported are two-sided. Full results are reported in Supplementary Tables 3–5. Estimate for fee waivers (top) and for care work participation, aspirations and goals and leadership (bottom) are not shown due to insufficient power to calculate effects. *P < 0.05, **P < 0.01, ***P < 0.001.

Figure 3 summarizes overall pooled effects sizes disaggregated by SSN modality (Fig. 3, top) and by indicators (Fig. 3, bottom). Pooled effects are reported in standardized units (Hedges’ *g*) using robust variance estimation (RVE) modelling, which corrects standard errors for dependency within studies that present multiple relevant effect estimates per outcome type. Supplementary Tables 3 and 4 give details for these estimations. Impacts overall for all SSN modalities (top) are positive and highly significant with a Hedges’ *g* of 0.107 (d.f. 89.6, *P* < 0.001, 95% CI 0.085 to 0.129, *n* = 1307). When disaggregated by type of SSN, pooled effects are significant from UCTs (Hedges’ *g* = 0.128, d.f. 54.7, *P* < 0.001, 95% CI 0.097 to 0.159, *n* = 817), social care (Hedges’ *g* = 0.122, d.f. 8.98, *P* < 0.001, 95% CI 0.071 to 0.174, *n* = 105) and asset transfers (Hedges’ *g* = 0.115, d.f. 14.7, *P* < 0.001, 95% CI 0.071 to 0.160, *n* = 216). In addition, the pooled coefficient on public work programmes (Hedges’ *g* = 0.127, d.f. 7.67, *P* = 0.031, 95% CI 0.015 to 0.239, *n* = 106) is similarly large in magnitude, but significant at a lower level. Finally, impacts from CCTs (Hedges’ *g* = 0.059, d.f. 18.6, *P* = 0.019, 95% CI 0.011 to 0.108, *n* = 167) and in-kind transfers (Hedges’ *g* = 0.071, d.f. 5.97, *P* = 0.123, 95% CI −0.026 to 0.169, *n* = 112) are positive, but relatively smaller and the latter is insignificant. Overall, heterogeneity between studies is high, with most *I*^2^ values in the 80–95% range and most *τ*^2^ values exceeding 0.02, including substantial diversity in interventions, settings, study designs or operationalization of outcomes. Supplementary Table 5 replicates effects by domain of outcome, showing relatively consistent impacts across economic achievement and agency, with some exceptions. For example, effects for both CCT domains are insignificant but of comparable magnitude to the pooled effect; impacts for in-kind transfers driven by the agency domain and impacts for care services and public work programmes are driven by the economic achievement domain.

Figure 3 displays effects by indicator type and domain (Fig. 3, bottom), showing similar magnitude of Hedges’ *g* for economic achievement (Hedges’ *g* = 0.113, d.f. 72.3, *P* < 0.001, 95% CI 0.085 to 0.142, *n* = 843) and agency domains (Hedges’ *g* = 0.101, d.f. 55.3, *P* < 0.001, 95% CI 0.063 to 0.139, *n* = 462). Supplementary Table 6 shows details underlying these estimates. Strong effects for economic achievement are seen for assets (Hedges’ *g* = 0.235, d.f. 14.9, *P* < 0.001, 95% CI 0.125 to 0.345, *n* = 83), savings (Hedges’ *g* = 0.229, d.f. 19.9, *P* < 0.001, 95% CI 0.147 to 0.311, *n* = 75), expenditures (Hedges’ *g* = 0.177, d.f. 17.9, *P* = 0.002, 95% CI 0.072 to 0.282, *n* = 55), labour force participation (Hedges’ *g* = 0.106, d.f. 42.6, *P* < 0.001, 95% CI 0.058 to 0.154, *n* = 155) and productive work intensity (Hedges’ *g* = 0.075 d.f. 48.9, *P* < 0.001, 95% CI 0.044 to 0.106, *n* = 370). Impacts for debt and loans (Hedges’ *g* = 0.105, d.f. 7.98, *P* = 0.233, 95% CI −0.082 to 0.291, *n* = 21) and care work intensity (Hedges’ *g* = -0.015, d.f. 14.2, *P* = 0.467, 95% CI −0.058 to 0.028, *n* = 62) are insignificant and care work participation has insufficient power to estimate effects. Significant pooled effects are seen for agency outcomes of voice (Hedges’ *g* = 0.172, d.f. 11.0, *P* = 0.011, 95% CI 0.048 to 0.297, *n* = 49), autonomy and self-efficacy (Hedges’ *g* = 0.105, d.f. 32.1, *P* = 0.001, 95% CI 0.046 to 0.165, *n* = 152) and decision-making (Hedges’ *g* = 0.087, d.f. 37.5, *P* < 0.001, 95% CI 0.036 to 0.137, *n* = 238). Meanwhile, aspirations and goals, as well as leadership have insufficient effect sizes and statistical power to estimate pooled effects. Similar to previous pooled estimates, most *I*^2^ values are in the 80–95% range, and most *τ*^2^ values exceed 0.02, thus pointing to substantial heterogeneity and variability between studies.

We provide a number of sensitivity analyses and robustness checks for these main results and report these in more detail in the Methods. First, we replicate results with an alternative model, a multilevel random-effects model with robust standard errors clustered at the study level. We find very similar results with only slight changes to point estimates and 95% CIs at the second or third decimal. In addition, we conduct a robustness check removing influential effects using visual inspection of Cook’s *D*, again finding very similar effects^19^ (Supplementary Fig. 3 and Supplementary Table 7). Finally, we provide a visual representation of pooled effects using the orchard plot, an alternative to the forest plot, for estimations with a large number of effects. We report these in Supplementary Fig. 4 (by outcome domain) and Supplementary Fig. 5 (by SSN type).

### Meta-regression

Table 2 reports RVE meta-regression results by background characteristics, grouped into three levels: study, intervention and effect (characteristics are presented in Table 1 and defined in Supplementary Table 8). The left panel uses a set of basic controls consisting of study quality factors, while the right panel uses a set of extended controls at each level (study, intervention and effect levels). Overall, significant correlations are largely consistent between basic and extended control models; however, we prefer the latter and thus note findings for this model where possible when summarizing results. At the study level, studies in Europe and Central Asia are associated with higher effect sizes, as compared with sub-Saharan Africa (the omitted category) (*β* = 0.526, d.f. 16.38, *P* < 0.001, 95% CI 0.435 to 0.618), while studies in Latin America and the Caribbean are associated with smaller effect sizes (*β* = −0.063, d.f. 24.21, *P* = 0.025, 95% CI −0.117 to −0.008, although only in the basic control model). However, the result for Europe and Central Asia is driven by one study with a high benefit level and thus should be interpreted with caution. At the intervention level, there is evidence that CCTs are associated with smaller effect sizes as compared with UCTs (the omitted category), although only in the extended control model (*β* = −0.075, d.f. 31.79, *P* = 0.020, 95% CI −0.137 to −0.012). Likewise, pilot interventions are associated with smaller effect sizes (as compared with mid-level interventions) (*β* = −0.079, d.f. 34.13, *P* = 0.013, 95% CI −0.141 to −0.018). At the effect level, as compared with productive work intensity, savings (*β* = 0.134, d.f. 18.97, *P* = 0.002, 95% CI 0.057 to 0.211), assets (*β* = 0.127, d.f. 11.38, *P* = 0.010, 95% CI 0.036 to 0.217) and voice (*β* = 0.113, d.f. 5.95, *P* = 0.048, 95% CI 0.001 to 0.224, only in the basic control model) are associated with higher magnitude effects. No other effect-level outcomes show significance. We conduct a variety of robustness checks varying the cut-offs and functional form of these indicators but find no additional meaningful correlations. *I*^2^ values exceed 90% in all cases, pointing to substantial variability between studies and suggesting that covariates do not sufficiently explain this variation. We provide a summary of a sensitivity analysis of these findings using the multilevel random-effects model in the Methods.

**Table 2:** RVE meta-regression results across all outcomes by publication, implementation and effect characteristics

	Basic controls			Extended controls		
	Coefficient	SE	*I*^2^	Coefficient	SE	*I*^2^
Panel A: study level						
Region (omitted = sub-Saharan Africa)						
South Asia	0.033	(0.029)	91.78%	0.046	(0.029)	91.95%
Latin America and the Caribbean	−0.063*	(0.026)	“	−0.043	(0.046)	“
Middle East and North Africa	−0.009	(0.041)	“	0.017	(0.047)	“
East Asia and Pacific	−0.070	(0.037)	“	−0.045	(0.044)	“
Europe and Central Asia	0.519***	(0.023)	“	0.526***	(0.043)	“
Income group (omitted = lower-middle)						
Low income	0.038	(0.029)	91.63%	0.044	(0.034)	“
Upper-middle income	−0.020	(0.025)	“	0.008	(0.041)	“
Fragile setting	−0.010	(0.037)	91.89%	−0.019	(0.041)	“
Urban setting	−0.033	(0.026)	91.89%	−0.014	(0.030)	“
Panel B: intervention arm level						
Intervention type (omitted = UCT)						
CCT	−0.055	(0.032)	91.79%	−0.075*	(0.031)	92.64%
Food, voucher or in-kind transfers	−0.046	(0.049)	“	−0.045	(0.055)	“
Asset transfers	0.002	(0.031)	“	−0.014	(0.039)	“
Public work programmes	0.011	(0.049)	“	0.056	(0.050)	“
Social care services	−0.009	(0.029)	“	−0.009	(0.042)	“
Implementer (omitted = NGO, UN or other)						
Government	−0.008	(0.027)	91.88%	−0.026	(0.033)	“
Researchers	−0.002	(0.035)	“	−0.025	(0.041)	“
Scale of implementation (omitted = mid-level)						
Pilot	−0.058*	(0.026)	91.79%	−0.079*	(0.030)	“
At scale	−0.028	(0.028)	“	−0.028	(0.034)	“
Value of economic benefit (omitted = first tercile)						
Second tercile	−0.018	(0.025)	91.88%	−0.004	(0.028)	“
Third tercile	−0.008	(0.031)	“	−0.005	(0.035)	“
Targeting approach						
Poverty targeted	−0.001	(0.026)	91.93%	−0.010	(0.034)	“
Gender targeted	0.012	(0.024)	“	0.036	(0.040)	“
Plus components (omitted = no Plus)						
Gender-neutral	0.022	(0.050)	92.12%	0.049	(0.053)	“
Gender-sensitive	0.026	(0.043)	“	0.037	(0.053)	“
Economic	0.051	(0.032)	“	0.059	(0.044)	“
Health or protection	0.005	(0.045)	“	0.007	(0.048)	“
Training or information	−0.008	(0.037)	“	−0.010	(0.043)	“
Other	−0.011	(0.041)	“	−0.021	(0.041)	“
Female sample age (omitted = ≤24 years)						
Age: 25–39 years	0.002	(0.031)	91.96%	−0.019	(0.039)	“
Age: 40+ years	0.042	(0.041)	“	0.053	(0.051)	“
Panel C: effect level						
Outcome category (omitted = productive work intensity)						
Labour force participation	0.045	(0.023)	91.26%	0.044	(0.025)	91.40%
Care work participation	0.227	(0.162)	“	0.229	(0.158)	“
Care work intensity	−0.049	(0.029)	“	−0.050	(0.029)	“
Savings	0.138**	(0.037)	“	0.134**	(0.037)	“
Debt or loans	0.021	(0.052)	“	0.027	(0.054)	“
Assets	0.125*	(0.041)	“	0.127*	(0.041)	“
Expenditure	0.110	(0.054)	“	0.107	(0.055)	“
Decision-making	0.031	(0.034)	“	0.025	(0.034)	“
Autonomy and self-efficacy	0.028	(0.034)	“	0.025	(0.032)	“
Voice	0.113*	(0.046)	“	0.115	(0.048)	“
Duration of the intervention (omitted = <12 months)						
12+ months	0.016	(0.028)	91.88%	0.019	(0.026)	“
Time post-intervention at survey (omitted = <12 months)						
Over 12+ months	−0.011	(0.023)	91.88%	−0.001	(0.022)	“

All estimates are from RVE regressions with *n* = 1,307 effects and 93 studies. CCT, conditional cash transfer; SE, standard error; UCT, unconditional cash transfer. *P < 0.05, **P < 0.01, ***P < 0.001. *I*^2^ estimates are entered once per model and apply to the remaining estimates in each category; double quote symbol (") indicates ‘same as above’. Basic control estimates are obtained from separate regressions by background characteristic, controlling for publication type, year-of-publication splines, an indicator for low- or medium-quality assurance, and whether the publication reports an individual (rather than cluster) RCT. Extended control estimates additionally control for all other covariates at the same level (study, intervention or effect).

### Cost–benefit analysis

A total of 25 papers (or 22%) representing 23 studies (or 25%) reported some measure of cost–benefit within studies. Due to the low percentage of studies reporting these measures, as well as the diversity of measures reported, we summarize these in narrative form. The most common measure reported were benefit–cost ratios (BCRs) (19 studies), followed by internal rate of return (IRR) (12 studies), cost-effectiveness (5 studies), net present value (4 studies) and economic multipliers (2 studies). Supplementary Table 9 presents these studies sorted by size of the measure, starting with BCR (the most reported measure), followed by remaining measures. Studies reporting cost–benefit results are overwhelmingly evaluating UCTs or asset transfers (or a combination of the two), while the remaining evaluate CCTs (two studies), public works (one study) or social care services (1 study). Overall, BCRs (or range of estimates) are positive and include within bounds a number greater than one in all but one study—ranging from a high of 16.9 for a UCT in Tunisia^20^ to −1.98 for a combined asset and in-kind transfer in Honduras^21^. Studies use a variety of methods and assumptions in calculations; however, only three (Colombia, Egypt and Nicaragua) take women’s labour force participation or earnings into account in benefit calculations, while most base calculations exclusively on household consumption and asset accumulation. As interventions typically deliver a wide range of additional benefits, including women’s economic achievements, which contribute directly to households’ well-being and increase resilience against shocks, BCRs summarized here are probably lower bounds. Ten studies report exclusively positive IRR estimates, ranging from a high of 73% in a UCT in Niger^22^ to a low of 6% in an asset transfer and UCT intervention in Ghana^21^. The two remaining studies report IRR lower bounds that include negative values; however, these occur under assumptions that impacts would dissipate immediately or after 2 years^22,23^. The IRRs for most reported interventions indicate that most interventions would pay for themselves (break even points) after a nominal number of years. Five studies reported cost-effectiveness measures, typically comparing different study treatment arms. For example, a UCT versus micro-franchising experiment in urban Kenya targeting young women reported UCT arms were generally more cost-effective^24^. However, in two other UCT experiments, the cost-effectiveness of a psychological plus component alone^25^ or a ‘full package’ arm including numerous plus components was found to be 1.6–1.7 times higher than the UCT alone^22^. Finally, economic multipliers in two different UCTs in Zambia were found to be 1.6–1.7, indicating households spent or saved 60–70% more than they received via transfers^26^. Despite these promising results, as previously mentioned, few studies explicitly included gender-specific benefits in their measures (five studies included at least one woman’s outcome in calculations, and three focused on women’s outcomes). This indicates that the benefits to women aggregated in this review, and gender equality considerations more generally, are largely absent from cost–benefit calculations.

## Discussion and policy implications

We show highly significant pooled effects of SSNs on women’s economic achievement and agency. These effect sizes are similar to those found in previous meta-analyses of vocational training on female youth employment and earnings (50 studies, pooled effect size 0.109)^17^ but smaller than those found for economic self-help groups considering empowerment outcomes (for example, political, social and economic agency; 7 studies, pooled effect size 0.18)^14^ and larger than those found for microcredit and savings interventions on a range of similar outcomes to ours (17 (12) studies, pooled effect sizes: 0.027 economic empowerment (0.019 agency))^15^ (Supplementary Table 10). They are also similar to effect sizes found for the impact of cash transfers on subjective well-being and mental health (45 studies, pooled effect size 0.13)^27^ or for economic empowerment interventions on intimate partner violence (16 studies, pooled effect size 0.09)^28^. Our analysis reflects a substantial increase in the evidence base from previous reviews and reinforce that SSNs benefit women, increasing their agency and economic standing.

When examining effects by outcome category, we see strong support for SSNs boosting and benefitting women’s labour force participation, productive work intensity, savings, assets and expenditures. Favourable impacts align with previous reviews and analyses of cash transfers in LMICs—showing little evidence of ‘dependency’ effects, whereby participants may reduce work effort due to benefits (for example, there is a negative relationship between the intervention and either labour force participation or work intensity)^29,30^. In addition, we show no evidence of increased care work intensity and debt or loans (both with null effects). While it would be encouraging to see reductions in care work intensity, signalling a possible shift in norms around men’s involvement in domestic work—the number of studies that measure care work on the intensive margin is still relatively low (16 studies). We also find strong evidence of positive impacts on voice, autonomy, self-efficacy and decision-making; however, sample sizes are insufficient to estimate effects on aspirations, goals or leadership. Decision-making indicators make up a relatively smaller proportion of all agency indicators (52%) compared with previous reviews; nonetheless, they suffer from measurement limitations, including lack of specificity, ambiguity regarding joint decision-making, and variability in indicator construction, among others^31–33^. There are also few estimates for combined (aggregate) measures of agency, indicating that most experimental studies SSNs have not attempted to holistically measure agency as an overall construct.

Our second research objective was to examine variation in impacts by SSN type, as well as by design and contextual factors. The most striking differences emerging for CCTs and food, voucher or in-kind transfers—for which pooled effects are qualitatively smaller in magnitude and, for the latter, insignificant. The differences for CCTs emerge in the meta-regression models (as compared with UCTs) while controlling for other intervention-level factors that might explain this difference (that is, value of benefits and gender targeting). One hypothesis may be that that CCTs (like in-kind transfers) may restrict women’s choices regarding use of benefits, thus giving her less autonomy over spending. In addition, due to conditionalities, there may be some disempowering aspects of programmes, leading to reinforcement of gender roles, as has been found in other studies^10,11^. For example, if women are responsible for attending required trainings or ensuring children attend health check-ups, this may both reduce her time to engage in productive work (particularly if meetings are far away or at inconvenient times), as well as reinforce her role as a primary caretaker. For these reasons, there have been increasing calls to drop conditionalities, in order to ensure the most marginalized women can maintain eligibility and to avoid overburdening them with programme compliance requirements^6^. Our evidence supports these recommendations; however, we note that we cannot fully disentangle conditions from other design features of such programmes.

Our final research objective was to assess the cost–benefit evidence associated with included studies. A minority of studies included costing estimates, and among those that did, few considered impacts on women as part of benefit calculations, instead relying on household-level impacts. Thus, while our summary confirms that interventions largely appear to be smart investments, additional shifts are needed for cost–benefit analysis to incorporate a gender lens. This shift would necessitate an approach that could account for intrahousehold benefits or count (aggregate) non-economic benefits such as agency measures.

A key limitation of the current analysis is this lack of concrete programme design and contextual factors that appear to meaningfully predict effectiveness. This could be in part due to power issues in the meta-regression, as well as generalized high heterogeneity of studies. For example, the current SSN literature stresses the importance of targeting women and providing complementary programming to deliver holistic impacts—yet we do not observe these design components being associated with larger effect sizes. These findings align with two recent meta-analyses: one on early childhood outcomes concluding that cash plus was rarely more effective than cash alone^34^, and the second finding that many educational gains may be made for girls through non-targeted programmes^35^. Nonetheless, our results do not lend themselves to strong conclusions, in part because of the heterogeneity of plus programming in our sample, on the one hand, and the low variability in gender targeting, on the other. Relatedly, although we find little evidence of publication bias, we cannot investigate researchers’ decisions to measure women’s outcomes or analyse outcomes disaggregated by gender in the first place. It could be that the studies that produce impacts on women are those most likely to have objectives related to and design considerations appropriate for benefitting women. If this is the case, we may be both underestimating the importance of design factors—for example, the contribution of gender-informed design—as well as overestimating the potential for gender equality impacts. In addition, it is well established that impacts of SSNs can vary over time, including the fade-out of initial impacts or the accumulation of longer-term impacts, including intergenerational impacts. Our sample is primarily short term—on average, interventions are 12 months long and follow-up surveys are 14 months later. Thus, impacts may evolve as more studies conduct longer-term follow-ups, and it remains unclear how this will affect conclusions. Finally, the fact that we include only experimental studies and, despite searches, did not identify studies in French or Spanish limits the generalizability of our findings.

Our review shows that SSNs improve women’s economic achievements and agency in LMICs—a conclusion that holds for most intervention types. There is significant momentum at the global level to invest in systems and programmes that ensure women benefit equally and to ensure gender gaps do not widen^5,6,36^. Our results indicate that SSNs have the greatest potential to benefit women if they are designed using unconditional modalities with cash, asset or care-based benefits. In addition, we encourage policymakers and practitioners to adopt existing practical recommendations, including eliminating barriers that may limit women’s access to SSNs, extending coverage to previously excluded groups, investing in linkages with complementary services and prioritizing women’s leadership and political voice in decision-making structures^6^. Researchers should continue to close evidence gaps, with respect to understudied geographies, including in fragile settings, SSN typologies and outcomes. Future research should rigorously test design and operational components, unpack the role of contextual factors, including gender norms in delivering benefits for women, and expand cost–benefit analysis to incorporate a gender lens.

## Methods

The review follows best practice for systematic reviews and meta-analyses in social science, including guidance from PRISMA. Full PRISMA checklists are provided in Supplementary Table 11 (overall) and Supplementary Table 12 (abstracts).

### Sample construction

Inclusion and exclusion criteria for qualifying studies and impact estimates were prespecified in a systematic review and meta-analysis protocol (PROSPERO #CRD42022382158). The protocol was registered in December 2022 and available on the PROSPERO website, and no amendments were made after this date. We briefly describe these criteria with respect to eight parameters: intervention, setting, population, outcomes, methodology, time frame, type of publication and language. Supplementary Table 1 presents inclusion and exclusion criteria in greater detail.

The review focused on SSNs, broadly following the World Bank’s ASPIRE categorization of non-contributory programming, including: UCTs and CCTs, food, vouchers or in-kind transfers, productive asset transfers, public works programmes, fee waivers and subsidies, and social care services^12^. ASPIRE also includes school feeding as a modality, but we omitted this from the search stage, as we anticipated school feeding to have fewer links to our target outcomes—and few studies examining these linkages. For the social care service category, we consider non-contributory (and non-cash) family support services, including childcare, eldercare, care for people with disabilities, and child protection services—as long as the intervention conveyed an economic benefit (vouchers, free access, subsidized care and so on)^4^. We include interventions implemented as stand-alone or those bundled with plus or complementary programming (for example, layered trainings, benefits or linkages to other health, and social or economic services), as long as the associated impacts included at least one SSN intervention. The latter is often the case for graduation programmes, which traditionally include a lumpy asset transfer (for example, livestock), in addition to monthly or bimonthly consumption support (UCTs), and a bundle of additional training or group-based activities. We recognize the complexities of including evaluations of SSN that have many other bundled benefits, both multiple economic benefits as well as diverse complementary programming. However, we wanted to be purposefully inclusive, as there is a long-standing history of SSNs delivering complementary programming, dating back to the early 2000s with CCTs such as *Progresa* (Mexico), *Bolsa Familia* (Brazil) and other programmes in Latin America^37^. In fact, most studies identified included at least one plus component (59% of the 135 intervention arms in our sample). Therefore, we aim to unpack the potential contribution of plus components in analysis directly, while acknowledging that undoubtably we miss nuance across intervention types. We do not include evaluations of plus components alone.

We included studies in LMICs that measured adult women’s outcomes (samples primarily over the age of 18 years). Qualifying outcomes included measures of women’s economic achievement or agency. Categories of economic achievement followed the Center for Global Development’s economic empowerment compendium^38^, in the following eight domains: (1) labour force participation (extensive margin, such as employment, self-employment, operated a business and so on), (2) productive work intensity, earnings or quality (intensive margin, such as total income, hours worked, profit, type of contract, level of benefits and so on), (3) unpaid care work (extensive margin, such as conducted any unpaid care work, use of care services and so on), (4) unpaid work intensity or quality (intensive margin, such as hours worked, quality or conditions around work and so on), (5) savings (such as any savings, amount saved, use of financial services for savings and so on), (6) debt or loans (such as any outstanding loan, borrowed on credit, amount outstanding and so on), (7) assets (such as ownership, rights around durable or productive assets, including livestock, business assets, information technology and so on) and (8) expenditures (such as personal expenditure on durable goods, expenditures for own business and so on). Categories of agency followed the conceptualization by the Bill and Melinda Gates Foundation’s model of empowerment^39^, in the following five domains: (1) decision-making (for example, household or individual role or joint decision-making or bargaining power and so on), (2) autonomy and self-efficacy (for example, power, control, mobility, independence, confidence, self-worth and so on), (3) aspiration and goals (for example, stated aspirations or goals related to any other qualifying measure), (4) voice (for example, ability to speak in public, membership or participation in groups, voting and collective agency) and (5) leadership (for example, leadership positions, participation in local governance and so on). For each category and domain, aggregate indicators or indices were also considered as long as they included most qualifying indicators. Outcomes at the household level were not eligible.

Experimental studies were included that measured intent-to-treat effects, whereas quasi-experimental or non-experimental studies as well as those measuring only treatment-on-the-treated effects were excluded. Consistent with social science guidance for meta-analyses, academic articles published in journals, as well as grey literature including technical reports, working papers, preprints and discussion papers were included, as long as they sufficiently explain and present methodology and results^40^. Studies in English, French or Spanish were included from 2003 onwards. From previous reviews and from our understanding of the evolution of experimental impact evaluations considering gender in the SSN literature in LMICs, starting from 2003 ensured few (if any) publications would be left out.

### Searches and selection of studies

The overview of search and selection of studies is presented in Fig. 1 as a PRISMA flow diagram. Search strings were built using keywords for intervention type, population, outcome, methodology and setting and piloted in English using three databases (Scopus, Web of Science and PubMed). Seven well-known studies were used to pilot the search string, which represented a variety of SSN types and disciplinary foci and outcome measures. These studies were all published or released by 2022 to increase the likelihood they would be indexed in databases. Search strings were assessed on a case-by-case basis on their ability to identify these seven papers. Where papers were excluded, search strings were modified (where advantageous) and rerun until authors were satisfied with the inclusivity of the search process. Supplementary Table 13 provides the seven studies used to pilot the search string and their hits in the three pilot databases.

Searches were subsequently conducted in English across six databases comprising both social science and public health repositories starting in January and ending in February 2023 (Scopus, Embase, EconLit, Web of Science, PubMed and Google Scholar). Search strings in French and Spanish were produced using professional translators and replicated in Scopus, the database with the largest number of hits and largest compilation of non-English articles (see Supplementary Table 14 for search strings in English, French and Spanish). Following PRISMA guidelines, automated searches in English were conducted monthly in Scopus and integrated up until 1 December 2024. In addition, studies were compiled from websites of leading organizations working on primary impact evaluations of SSNs, through identification of prior and derivative works of nine SSN review papers using artificial intelligence literature review tools (www.connectedpapers.com), from existing evidence gaps maps and solicitation from experts in the gender and SSN field^1,3,8,9,16,28,41–43^. Organizational websites searched included World Bank Open Knowledge Repository, the World Bank Gender Innovation Labs, the Asian Development Bank, the Inter-American Development Bank, the African Development Bank, J-PAL, Innovations for Poverty Action, the International Food Policy Research Institute, Oxford Policy Management, Socialprotection.org, the Transfer Project and UNICEF Office of Research–Innocenti. Our multipronged strategy increased the likelihood that key papers would not be excluded. However, it is possible that eligible studies were not identified for a number of reasons, such as descriptions that did not clearly classify interventions as SSNs (per our keywords) or the inclusion of recently released studies.

Studies were screened and assessed for eligibility using Covidence (https://www.covidence.org/) software and documented using PRISMA guidelines. Titles and abstracts of studies were screened by reviewer 1 in discussion with reviewer 2 where studies required a second opinion. Full texts of retrieved studies were further assessed for eligibility by reviewer 1, and qualifying effects within each eligible study were agreed upon by reviewers 1 and 2. Full texts of studies not available or additional supplementary information was solicited from corresponding or lead authors directly. For extraction and analysis, articles and papers examining the same impact evaluation were considered as one study, whereas papers presenting data from different impact evaluations were considered unique studies. For example, if five papers analysed the original *Progresa* evaluation data (1997–2001), these papers would all be included if they presented unique impacts (outcomes over time) and are considered one study. In this example, impacts would be included only once, typically the first time they were published. Exceptions occurred if earlier publications omitted essential information (for example, sample sizes and indicator definitions) or used modelling approaches that were incompatible with producing standardized effect sizes. Therefore, to be included, each paper needed to include unique estimates, rather than replicating estimates that already existed in previous publications. For pure replication studies, a different criterion was applied, whereby papers could still qualify for inclusion if they produced qualitatively different impacts (reversing either significance or sign of the impact). However, within our pool of eligible studies, no replication effort met these criteria; thus, our sample does not include dedicated papers that engage in pure replication.

The final analysis sample included 93 studies, 115 publications (or papers), 135 intervention arms and 1,307 effects. The final sample was compiled from 5,120 hits (4,146 from databases and 974 from other sources). After removal of 1,215 duplicates, a total of 3,905 abstracts were screened, and 345 full texts were screened. Among papers assessed for eligibility via full text screening, the most common reasons for exclusion were that the paper did not report on an eligible outcome (102 papers or 44%), did not evaluate an eligible intervention (45 papers or 20%) or reported effects in the wrong population (45 papers or 20%, typically reporting effects for households in general, among men, children or a pooled sample with no disaggregation). Papers excluded for ineligible interventions included those that strictly compared design features of a SSN without a non-SSN control group (for example, what is known as A/B testing, such as comparing mobile versus manual delivery of a UCT or comparing male versus female targeting of a UCT, without a control group)^44,45^. We excluded these studies as they are not comparable to randomized designs that included at least one SSN effect. Overall, nine studies (4%) were dropped because of other reasons, including incomplete reporting and low confidence in study results. Several of these cases involved studies that estimated impacts for women using an interaction term in a pooled model of both men and women, and we were unable to recover the information needed to calculate the direct effect on women alone. Supplementary Table 2 gives authors, year of publication, country of study, intervention type and name, and number of aggregate impacts included in the meta-analysis for each included study (sorted alphabetically by country of study, date and author last name). A full reference list of all primary papers can be found at the end of the Supplementary Information (ordered alphabetically by first author’s last name).

In addition to the exclusions documented in Supplementary Fig. 1, we excluded outlier effects, defined as those with Hedges’ *g* ≤ −1 or ≥1. In total, 34 observations, or approximately 3% of the analysis sample, were dropped that have Hedges’ *g* outside this acceptable range. Before dropping coefficients, they were rechecked to ensure accuracy of the extraction. All but two of these coefficients were positive, with several relating directly to the type of SSN. For example, some of these coefficients reflected labour force participation effects in a public works programme, use of care services in a childcare intervention, or effects on savings in a UCT. In this case, we would expect effects to be large, as they in part reflect direct inputs or activities of a particular intervention. As many of these coefficients were credible, despite being outside the acceptable range, and only two were negative, this indicates that dropping outliers is a conservative approach to estimating pooled effects. Of note regarding the representativeness of the sample, despite conducting primary searches in French and Spanish, all publications in the final analysis were in English.

### Study extraction and descriptive analysis

A detailed guide was developed to facilitate standardized extraction of study components and piloted by all study authors. Primary extraction was randomly assigned to either reviewer 3 or reviewer 4 with secondary checks of all information completed by either reviewer 1 or reviewer 2. In exceptional cases, primary extraction was completed by reviewer 1 or reviewer 2—primarily for studies with complex designs or analyses requiring additional steps, such as analyses including both men and women with heterogeneity assessment, or cases where replicating study results with primary data was necessary to obtain complete information. Coefficients representing qualifying outcome indicators were extracted for all unique follow-up periods (timepoints) for the ‘preferred model’ of each unique indicator construction. We did not extract coefficients of robustness checks duplicating preferred specifications or of heterogenous effects (unless these were gender specific and, thus, represented average impacts on women). Where papers were missing critical information necessary to calculate standard effect sizes or required complementary characteristics, corresponding or lead authors were contacted directly to obtain missing information. Alternatively, if information on programme design was available in a referenced or alternative existing paper, information was extracted from the additional paper. According to protocols, papers or effects were dropped if information was not obtained after a second follow-up email; however, in practice, many additional follow-ups were made to request information. Additional papers and studies identified and added through automated monthly searches were screened and extracted by one reviewer (eight studies in total). Descriptive analyses for study background characteristics and all descriptive graphs were generated using Stata version 15.0.

Costing estimates were extracted for a subset of studies reporting any qualifying measure, including any formal analysis of the following: (a) BCR, (b) IRR, (c) net present value, (d) cost-effectiveness or (e) economic multiplier. The inclusion approach for different cost-related estimates was purposefully flexible to allow a diversity of methodological approaches, but required the comparison of costs with some form of benefits originating from the economic component of the SSN. In one case of a CCT for youth entrepreneurship in Sierra Leone, costing estimates were presented yet not included in our summary^46^. This was because the cost-effectiveness measures were based on full sample estimates for profits, capital stock and household consumption, yet the impacts for female youth (approximately 50% of the sample) were substantially different as compared with male youth. Therefore, we consider the full sample cost-effectiveness estimates as misleading if applied to exclusively to the female subsample.

### Risk of bias

Studies were assessed for risk of bias at using a modification of the Joanna Briggs Institute (JBI) critical appraisal tool for experimental studies^47^. Similar to extraction, bias assessment was randomly assigned to either reviewer 3 or reviewer 4, with secondary checks of all information completed by either reviewer 1 or reviewer 2. As prespecified, we do not exclude any eligible studies on the basis of the results of the quality assessment alone; however, we account for study quality in the meta-regression analyses. We follow a modified version of cut points from previous studies using the JBI tool for experimental studies as follows: low (<50% items ‘yes’), medium (50–70% items ‘yes’) and high (>70% items ‘yes’)^48^. Supplementary Table 15 presents the revised criteria (scores range from 0 to 10) and deviations from the original tool. Deviations were primarily to accommodate standard practice for social science experimental studies. For example, SSN trials would be unable to include aspects such as blinding of participants or implementers to the treatment condition after the baseline period. These criteria are more relevant for health or medical trials that use dedicated placebo treatments and arguably should not be conditions required for quality assessments in social science trials.

Overall, quality assessments deemed studies to be overwhelmingly high quality, probably reflecting general standardized design and reporting for experimental studies in the economic development field. Scores ranged from 60% (8 studies) to 100% (5 studies), with 90% being the modal category (39 studies). The mean overall score among included studies is 81%, and over 65% of papers qualify as ‘high quality’ (>70% of items scored ‘yes’), with the remaining studies classified as medium quality. No studies scored as low quality. The highest-scoring single items were for true randomization (item 1), baseline balance (item 2) and comparability of outcome measurement between study arms (item 8), while the lowest were for reporting on appropriate power (item 9) and blinding of participants and data collectors at baseline (items 3 and 4). Quality assurance scores by study are reported in Supplementary Table 2. Given the overall high quality of the studies and our control for quality assurance in all meta-regression analyses (using an indicator equal to 1 if the study was medium rather than high quality), we have high confidence in the results.

### Aggregation of effect sizes

To aggregate estimates across studies, we standardized treatment effects for all outcomes. Standardized effect sizes are scale-free measures that allow comparison of the magnitude and direction of treatment effects across different studies. To guide standardization, we generally follow the equations presented in the book *Practical Meta-Analysis* and cross-check our calculations using the online meta-analysis calculator hosted by the Campbell Collaboration^49^. In the case that publications present results using follow-up means only (19 effects, or 1% of the sample), we transformed treatment effects into the standardized mean difference, Cohen’s *d*, calculated as

1 $$d=\,\frac{\mathrm{Yt}-\mathrm{Yc}}{{\mathrm{SD}}_{\mathrm{pooled}}},$$

where Yt and Yc represent the means in the treatment and control group and SD_pooled_ is the pooled standard deviation. We converted effects from regressions on logged dependent variables (18 effects or 1% of the sample) to unstandardized coefficients. For four coefficients where authors reported preferred effects using dependent variables transformed with the inverse cosine, we substituted effects using winsorized raw indicators from the authors’ appendix materials; the sign and significance of these alternatives were consistent with the preferred effects^50^.

Treatment effects expressed as unstandardized regression coefficients *β* were converted to Cohen’s *d* using

2 $$d=\frac{\beta }{{\mathrm{SD}}_{\mathrm{pooled}}\,},$$

where SD_pooled_ is given by

3 $${\mathrm{SD}}_{\mathrm{pooled}}=\sqrt{\frac{\frac{{\mathrm{sd}}^{2}\left(\mathrm{nt}+\mathrm{nc}-1\right)-{\beta }^{2}(\mathrm{nt}\times \mathrm{nc})}{\mathrm{nt}+\mathrm{nc}}}{\mathrm{nt}+\mathrm{nc}-2}},$$

and where nt and nc are the sample sizes in the treatment and control group, respectively, an sd^2^ is calculated on the basis of the standard error of the regression coefficient using

4 $${\mathrm{sd}}^{2}=\mathrm{SE}\sqrt{\mathrm{nt}+\mathrm{nc}}.$$

In total, 1,104 effects, or 85%, were from unstandardized coefficients from ordinary least-squares regressions. Treatment effects expressed as standardized regression coefficients (149 effects or 11% of the sample) were first translated into unstandardized regression coefficients and then converted to Cohen’s *d*.

Treatment effects expressed as odds ratios (OR) (six effects or <1% of the sample) were converted to *d* using

5 $$d=\frac{(\mathrm{ln}(\mathrm{OR})\sqrt{3})/}{{\rm{\pi }}}.$$

Lastly, we converted all Cohen’s *d* into Hedges’ *g* values, which are corrected for potential bias that could result from small sample sizes, applying the following formula^49^:

6 $$g=d\left(1-\frac{3}{4N-9}\right),$$

where *N* is the total sample size, summarized across treatment and control groups. All items were coded such that higher scores (values) equalled more favourable economic achievement and agency. For example, we conceptualize additional hours of unpaid care work as an adverse outcome; thus, we reverse code the category for unpaid care work intensity. In addition, we code any single indicator as reverse if the construction of the outcome variable is unexpectedly signed (for example, woman’s lack of agency or unemployment, rather than an expression of agency as a positive construct or engagement in employment). Thus, all effects within a single category can be interpreted consistently as larger effects equalling a more favourable outcome.

### Meta-analysis and meta-regression

We use RVE meta-analysis to pool overall effect sizes as well as by SSN type and outcome. The RVE model allowed us to account for the nested structure of our data in which multiple outcomes were measured for the same sample of individuals in one study. Specifically, RVE corrects standard errors for dependency within studies that present multiple relevant effect estimates per outcome type^51,52^. Our unit of analysis were 93 study ‘clusters’, comprising a total of 1,307 effect size estimates, where reported effects for the same study are nested within the study cluster and assumed to correlate with each other. The correlated-effects RVE model accounts for the variation within and between study clusters as well as for the number of effect sizes per cluster by using weights, *w*_*ij*_. We followed the convention of assuming a within-cluster correlation of *r* = 0.8 (ref. ^52^).

The final RVE model is then given by

7 $${g}_{ij}=\alpha +{v_j}+{\varepsilon }_{ij},$$

where *g*_*ij*_ is the estimated effect size *i* in the study cluster *j*, $\alpha$ denotes the mean of the distribution of true effects across all clusters, *v*_*j*_ is a study-level random effect, var(*v*_*j*_) is the between-study variance in true effects and ${\varepsilon }_{ij}$ represents the residual for the *i*th effect size in the *j*th study cluster. The pooled Hedges’ *g* estimate is given by *g*_*ij*_, for all studies the qualifying outcome of interest. We estimated the RVE model (1) pooled across all outcome and SSN types, (2) disaggregated by outcome types (first by domain of economic achievement versus agency, then all outcome indicator categories separately) and (3) disaggregated by SSN type. Quantitative measures of heterogeneity (*τ*^2^), which captures the between-study variance, and consistency (*I*^2^), which estimates the percentage of between-study variance resulting from heterogeneity rather than random error, are also reported^53^. Due to the large number of individual effect sizes, we do not report summary statistics for each individual effect, including means for intervention and control groups, impact estimates and measures of precision (for example, CIs or standard errors).

To unpack the contributions of target group and setting characteristics, evaluation features, and intervention design, we conducted RVE meta-regressions using two-tailed tests. We first ran a separate regression for each covariate independently, controlling for publication type, year-of-publication splines (published before 2015 or published after 2019), quality assurance (if medium quality) and if the publication was an individual RCT (instead of cluster). In a second model, we additionally controlled for factors at each level, running three regressions for each of study-, intervention- and effect-level characteristics. At the study level, we tested the region and income group of the country of study, fragility of the study setting (defined as the setting being actively in conflict, post-conflict or taking place during COVID-19 or another epidemic) and urbanicity of the study setting. At the intervention level, we tested the following characteristics: SSN type, SSN implementer, scale of the programme (pilot, mid-level or at-scale), value of the economic benefit, targeting of the programme (for example, if poverty-targeted or if gender-targeted), and if the SSN included a plus component (defined as any complementary layered or integrated programme activity), as well as the type of plus programming (defined as training or information components, economic components, health or protection components or other components), whether it was gender-neutral or gender-sensitive, and the age of the women included in the sample. As noted in Table 1, interventions may include more than one SSN. For example, they may include both an asset transfer and an UCT. In this case, for RVE pooled effects by SSN type, they are analysed in both categories but included in the meta-regression as their primary type (defined as the one with higher economic value). In total, there were 202 effects with more than one SSN intervention included in the analysis. One study included a third SSN; however, as it was the only study with a category of ‘Fee waiver or subsidy’, the final analysis coding dropped this third intervention^54,55^. At the effect level, we tested the outcome category, duration of intervention at follow-up measurement and time post-intervention at follow-up measurement. The duration of the intervention ranged from 1 to 60 months (mean of 12 months), while follow-up measurements ranged from zero (intervention still running) to 140 months (or nearly 12 years, mean of 14 months). Supplementary Fig. 2 shows the distribution of these two indicators overall in the sample, as well as by SSN type. Based on their distributions, we dichotomized both to be indicators of 12 months or longer to increase statistical power. These two indicators help determine whether the length of the intervention matters and whether effects fade or are sustained over time after the intervention ends. Supplementary Table 8 gives detailed descriptions of all indicators used as covariates.

For the RVE meta-regression, we augmented the model from equation (7) by including each of the above characteristics as ‘moderators’ (or covariates). The resulting mixed-effects model takes the following form:

8 $${g}_{{i}{j}}=\alpha +{\bf{X}}{\prime} \beta +{v}_{{j}}+{\varepsilon }_{{i}{j}},$$

where **X**′_*ij*_ is a vector of study-, intervention- and effect-level covariates included in the RVE meta-regression. Due to sample size issues, we were unable to run fully adjusted models aggregating covariates across all levels. We did not have a formal sample size determination method for the pooled analysis or the subsample analysis conducted. Rather, the sample size was determined by the number of eligible studies and qualifying effect sizes. However, meta-analyses and meta-regressions were performed only if the d.f. were greater than 4, as simulations have shown that the approximation of the variance distribution is not valid if d.f. <4 (ref. ^56^). To maximize statistical power, we ran meta-regressions only for pooled outcomes (economic achievement and agency), as the pooled effect sizes showed very similar magnitude and significance across the two domains. We do not formally test normality and equal variance tests for individual eligible effects; however, for all RVE models, we present measures of heterogeneity (*τ*^2^), which captures the between-study variance, and consistency (*I*^2^), which estimates the percentage of between-study variance resulting from heterogeneity rather than random error. The analyses were implemented in R 4.3.1 using the package ‘robumeta’^57^.

### Sensitivity analysis and robustness checks

We conducted several sensitivity analyses to verify the robustness of our results. First, as noted in Supplementary Table 8, we had missing observations for three characteristics for which information was not reported in publications and not recoverable via emails to authors (urban setting, age of woman and value of transfer). In total, urban classification was missing for 63 effects (5% of the sample), age of the woman was missing for 298 effects (23% of the sample) and value of the transfer was missing for 54 effects (4% of the sample). We replaced these missing observations with the average or modal category and conducted sensitivity analyses controlling for missingness; however, the results are robust to this variation, as well as to analyses using only the subset of effects with non-missing values.

Second, we ran sensitivity analyses with an alternative to our prespecified model, to assess the robustness of results to model choice. Instead of the RVE model, we estimated a multilevel random-effects model with robust standard errors clustered at the study level (based on the R package ‘metafor’). The pooled effect sizes and 95% CIs are very consistent across our main findings, with only slight changes in the magnitude of effects on the second or third decimal, largely showing the RVE model is slightly more conservative as compared with the multilevel model. The meta-regression results shown in Table 2 for the RVE model show some slight differences with the multilevel model in the significance of correlates; however, they are also largely consistent (detailed results not shown). Focusing on the extended control model, the previously significant coefficients on labour force participation, assets and expenditures become insignificant, whereas the following previously insignificant coefficients become significant: ‘other’ plus components (*P* < 0.05 level), care work participation and intensity (*P* < 0.05 level each) and over 12 months’ time to follow-up (*P* < 0.05 level). The latter results reinforce the few consistent design and contextual factors that seem to matter for overall impacts.

Third, following previous meta-analyses, we examine the robustness of our results to the inclusion of ‘influential’ effects^19,58^. We calculate Cook’s distance *D*, which measures how much the estimated pooled effect size would change if an effect was dropped from the analysis. Cook’s *D* is calculated based on the random-effects multilevel model using the R package ‘metafor’. There is no unified norm for determining a threshold for Cook’s *D* in meta-analysis, with previous studies using a predetermined threshold of *D* > 0.5 (ref. ^58^). We compute Cook’s *D* by intervention type and plot these in Supplementary Fig. 3. Only one effect is above the 0.5 threshold; therefore, as suggested by general guidance, we use visual inspection to identify thresholds for each type of SSN (see dotted blue lines ranging from 0.005 to 0.10; Supplementary Fig. 3). Correspondingly, visual inspection identifies several outlier effects within each SSN type, ranging from 4 (public works programmes) to 11 (UCTs). We then rerun our main RVE models, excluding these influential cases. The results of this analysis are reported in Supplementary Table 7. Effects are similar in magnitude and significance as compared with the main results; however, several point estimates are slightly lower (asset transfers, Hedges’ *g* = 0.109; social care services, Hedges’ *g* = 0.081) and one is slightly higher (public works programmes, Hedges’ *g* = 0.138). As is expected, the *I*^2^ values (consistency) decrease across models, ranging from 78% to 92%. We acknowledge that visual inspection is a purposefully subjective methodology; however, the results give additional confidence in the robustness of effects presented.

Finally, we sought to provide a visualization of our main results. As the standard forest plot is unpractical for a meta-analysis with a large number of effect sizes, we provide an alternative, the orchard plot (using the R package ‘orchaRd’)^59^. These plots are shown in Supplementary Fig. 4 (by economic achievement and agency domains) and Supplementary Fig. 5 (by SSN type). We note that these visualizations follow the aforementioned random-effects multilevel models with clustered standard errors (at the study level) rather than our preferred RVE models as the ‘robumeta’ package is not compatible with the ‘orchaRd’ package. Orchard plots visualize our full sample (rather than excluding influential effects), showing 95% CIs (thick lines) and overall heterogeneity, as indicated by prediction intervals (thin lines) around each effect estimate.

### Publication bias

Publication bias may occur if studies are selected by journals or self-censored by authors on the basis of the direction and statistical significance of the estimated treatment effect. We assessed publication bias based on visual inspection of a Doi plot and Luis Furuya-Kanamori (LFK) index to identify and quantify potential asymmetry of study effects^60,61^. Doi plots are a newer method for detecting publication bias in meta-analyses and found to have better diagnostic accuracy and sensitivity as compared with funnel plots and the Egger’s regression test, particularly if heterogeneity between studies is high^62^. The analyses were implemented in R 4.3.1 using the package ‘metasens’. In the absence of publication bias, the Doi plot should be visually symmetrical and the value of the LFK index should not lie outside the range of −1 to 1. Based on inspection of the Doi plot and the accompanying LFK index of 0.69, we conclude that our findings are probably not biased due to omitted studies (Supplementary Fig. 6).

### Reporting summary

Further information on research design is available in the Nature Portfolio Reporting Summary linked to this article.

## Supplementary information

Supplementary Information Supplementary Materials and Methods, Supplementary Figs. 1–6, Supplementary Tables 1–15 and supplementary works cited for included studies.

Reporting Summary

## Data Availability

Data are available via the Center for Open Science public repository at https://osf.io/brvy2/ (ref. ^63^). The data extraction form is not publicly available; however, requests can be made to the corresponding author.

## Code availability

All replication files and code used for the analysis are available via the Center for Open Science repository at https://osf.io/brvy2/ (ref. ^63^).

## References

[1] Bastagli, F. et al. The impact of cash transfers: a review of the evidence from low- and middle-income countries. *J. Social Policy* **48**, 569–594 (2019).

[2] Hidrobo, M., Hoddinott, J., Kumar, N. & Olivier, M. Social protection, food security, and asset formation. *World Dev.* **101**, 88–103 (2018).

[3] Peterman, A., Kumar, N., Pereria, A. & Gilligan, D. *Towards Gender Equality: A Review of Evidence on Social Safety Nets in Africa* (International Food Policy Research Institute, 2020).

[4] Gender-responsive age-sensitive social protection: a conceptual framework. *UNICEF Office of Research-Innocenti* https://www.unicef-irc.org/publications/1116-gender-responsive-age-sensitive-social-protection-a-conceptual-framework.html (2020).

[5] Cookson, T. P., Ebner, N., Amron, Y. & Kukreja, K. Social protection systems and gender: a review of the evidence. *Glob. Social Policy*10.1177/14680181231180507 (2023).

[6] Gavrilovic, M. et al. Gender-responsive social protection post–COVID-19. *Science* **375**, 1111–1113 (2022). 35271320 10.1126/science.abm5922

[7] *Social Protection Systems, Access to Public Services and Sustainable Infrastructure for Gender Equality and the Empowerment of Women and Girls; 2019 Commission on the Status of Women, Agreed Conclusions* (UN Women, 2019).

[8] Halim, D., Perova, E. & Reynolds, S. Childcare and mothers’ labor market outcomes in lower- and middle-income countries. *World Bank Res. Observ.* **38**, 73–114 (2023).

[9] Perera, C. et al. Impact of social protection on gender equality in low- and middle-income countries: a systematic review of reviews. *Campbell Syst. Rev.* **18**, e1240 (2022). 36913187 10.1002/cl2.1240 PMC9133545

[10] Cookson, T. P. *Unjust Conditions: Women’s Work and the Hidden Cost of Cash Transfer Programs* (Luminos, 2018).

[11] Molyneux, M. Mothers at the service of the new poverty agenda: Progresa/Oportunidades, Mexico’s conditional transfer programme. *Social Policy Admin.* **40**, 425–449 (2006).

[12] *The State of Social Safety Nets 2018* (World Bank, 2018); 10.1596/978-1-4648-1254-5

[13] Chang, W. et al. What works to enhance women’s agency: cross-cutting lessons from experimental and quasi-experimental studies. *Abdul Latif Jameel Poverty Action Lab* https://www.povertyactionlab.org/sites/default/files/research-paper/gender_womens-agency-review_2020-march-05.pdf (2020).

[14] Brody, C. et al. Economic self-help group programs for improving women’s empowerment: a systematic review. *Campbell Syst. Rev.* **11**, 1–182 (2015).

[15] Duvendack, M., Leon, M. D. A. & Filopoulos, J. *The Impact of Credit and Savings Interventions on Women’s Economic Empowerment and Agency: A Systematic Review and Meta-Analysis* (University of East Anglia, 2023).

[16] Lwamba, E. et al. Strengthening women’s empowerment and gender equality in fragile contexts towards peaceful and inclusive societies: a systematic review and meta-analysis. *Campbell Syst. Rev.* **18**, e1214 (2022). 36913184 10.1002/cl2.1214 PMC8904729

[17] Stöterau, J., Kemper, J. & Ghisletta, A. The impact of vocational training interventions on youth labor market outcomes: a meta-analysis. 10.2139/ssrn.4217580 (2022).

[18] Vaessen, J. et al. The effects of microcredit on women’s control over household spending in developing countries: a systematic review and meta-analysis. *Campbell Syst. Rev.* **10**, 1–205 (2014).

[19] Cook, R. D. in *International Encyclopedia of Statistical Science* (ed. Lovric, M.) 301–302 (Springer, 2011); 10.1007/978-3-642-04898-2_189

[20] Gazeaud, J., Khan, N., Mvukiyehe, E. & Sterck, O. With or without him? Experimental evidence on cash grants and gender-sensitive trainings in Tunisia. *J. Dev. Econ.* **165**, 103169 (2023).

[21] Banerjee, A. et al. A multifaceted program causes lasting progress for the very poor: evidence from six countries. *Science* **348**, 1260799 (2015). 25977558 10.1126/science.1260799

[22] Bossuroy, T. et al. Tackling psychosocial and capital constraints to alleviate poverty. *Nature* **605**, 291–297 (2022). 35477764 10.1038/s41586-022-04647-8 PMC9095470

[23] Botea, I., Brudevold-Newman, A., Goldstein, M., Low, C. & Roberts, G. Supporting women’s livelihoods at scale: evidence from a nationwide multi-faceted program. *SSRN Scholarly Paper* https://papers.ssrn.com/abstract=4560552 (2023).

[24] Brudevold-Newman, A., Honorati, M., Jakiela, P. & Ozier, O. *A Firm of One’s Own: Experimental Evidence on Credit Constraints and Occupational Choice* (World Bank, 2017); https://documents.worldbank.org/en/publication/documents-reports/documentdetail/428361487270218330/A-firm-of-ones-own-experimental-evidence-on-credit-constraints-and-occupational-choice

[25] Orkin, K. et al. *Aspiring to a Better Future: How a Simple Psychological Intervention Increases Investment* Working Paper No. 31735 http://www.nber.org/papers/w31735 (National Bureau of Economic Research, 2023).

[26] Handa, S., Natali, L., Seidenfeld, D., Tembo, G. & Davis, B. Can unconditional cash transfers raise long-term living standards? Evidence from Zambia. *J. Dev. Econ.* **133**, 42–65 (2018). 31396000 10.1016/j.jdeveco.2018.01.008 PMC6687333

[27] McGuire, J., Kaiser, C. & Bach-Mortensen, A. M. A systematic review and meta-analysis of the impact of cash transfers on subjective well-being and mental health in low- and middle-income countries. *Nat. Hum. Behav.* **6**, 359–370 (2022). 35058643 10.1038/s41562-021-01252-z

[28] Eggers del Campo, I. & Steinert, J. I. The effect of female economic empowerment interventions on the risk of intimate partner violence: a systematic review and meta-analysis. *Trauma Violence Abuse* **23**, 810–826 (2022). 33287669 10.1177/1524838020976088

[29] Banerjee, A., Hanna, R., Kreindler, G. E. & Olken, B. A. Debunking the stereotype of the lazy welfare recipient: evidence from cash transfer programs. *World Bank Res. Observ.* **32**, 155–184 (2017).

[30] Handa, S. et al. Myth-busting? Confronting six common perceptions about unconditional cash transfers as a poverty reduction strategy in Africa. *World Bank Res. Observ.* **33**, 259–298 (2018). 10.1093/wbro/lky003 PMC6830568 31693721

[31] Ambler, K., Doss, C., Kieran, C. & Passarelli, S. He says, she says: spousal disagreement in survey measures of bargaining power. *Econ. Dev. Cult. Change* **69**, 765–788 (2021).

[32] Peterman, A., Schwab, B., Roy, S., Hidrobo, M. & Gilligan, D. O. Measuring women’s decisionmaking: Indicator choice and survey design experiments from cash and food transfer evaluations in Ecuador, Uganda and Yemen. *World Dev.* **141**, 105387 (2021).

[33] Seymour, G. & Peterman, A. Context and measurement: an analysis of the relationship between intrahousehold decision making and autonomy. *World Dev.* **111**, 97–112 (2018).

[34] Little, M. T. et al. Effectiveness of cash-plus programmes on early childhood outcomes compared to cash transfers alone: a systematic review and meta-analysis in low- and middle-income countries. *PLoS Med.* **18**, e1003698 (2021). 34582447 10.1371/journal.pmed.1003698 PMC8478252

[35] Evans, D. K. & Yuan, F. What we learn about girls’ education from interventions that do not focus on girls. *World Bank Econ. Rev.* **36**, 244–267 (2022).

[36] Aliga, A. et al. Smart investment in global childcare requires local solutions and a coordinated research agenda. *BMJ Glob. Health* **8**, e012827 (2023). 37666575 10.1136/bmjgh-2023-012827 PMC10481721

[37] Handa, S. & Davis, B. The experience of conditional cash transfers in Latin America and the Caribbean. *Dev. Policy Rev.* **24**, 513–536 (2006).

[38] Buvinić, M., O’Donnell, M., Knowles, J. C. & Bourgault, S. Measuring women’s economic empowerment: a compendium of selected tools. *Center for Global Development* https://www.cgdev.org/publication/measuring-womens-economic-empowerment-compendium-selected-tools (2020).

[39] van Eerdewijk, A., Wong, F., Newton, J. & Vaast, C. A conceptual model of women and girls’ empowerment. *KIT Institute* https://www.kit.nl/project/empowerment-of-women-and-girls-conceptual-model-and-measurement-guidance/ (2017).

[40] Irsova, Z., Doucouliagos, H., Havranek, T. & Stanley, T. D. Meta-analysis of social science research: a practitioner’s guide. *J. Econ. Surveys* **38**, 1547–1566 (2024).

[41] Buvinić, M. & Furst-Nichols, R. Promoting women’s economic empowerment: what works?. *World Bank Res. Observ.* **31**, 59–101 (2016).

[42] Buvinić, M. & O’Donnell, M. Gender matters in economic empowerment interventions: a research review. *World Bank Res. Observ.* **34**, 309–346 (2019).

[43] Laszlo, S. *The Gender Transformative Potential of Graduation Programs* GrOW Research Working Paper Series No. 25 (Institute for the Study of International Development, McGill University, 2019); https://grow.research.mcgill.ca/publications/working-papers/gwp-2019-25.pdf

[44] Aker, J. C. Comparing cash and voucher transfers in a humanitarian context: evidence from the Democratic Republic of Congo. *World Bank Econ. Rev.* **31**, 44–70 (2017).

[45] Armand, A., Attanasio, O., Carneiro, P. & Lechene, V. The effect of gender-targeted conditional cash transfers on household expenditures: evidence from a randomized experiment. *Econ. J.* **130**, 1875–1897 (2020).

[46] Rosas, N. & Sabarwal, S. Public works as a productive safety net in a post-conflict setting: evidence from a randomized evaluation in Sierra Leone. *World Bank Group* https://openknowledge.worldbank.org/handle/10986/23916 (2016).

[47] Aromataris, E. & Munn, Z. JBI manual for evidence synthesis. *JBI*10.46658/JBIMES-20-01 (2020).

[48] Islam, M. A. et al. Prevalence of headache in patients with coronavirus disease 2019 (COVID-19): a systematic review and meta-analysis of 14,275 patients. *Front. Neurol.* **11**, 562634 (2020). 33329305 10.3389/fneur.2020.562634 PMC7728918

[49] Lipsey, M. W. & Wilson, D. B. *Practical Meta-Analysis* (Sage Publications, 2001).

[50] Rodríguez-Barranco, M., Tobías, A., Redondo, D., Molina-Portillo, E. & Sánchez, M. J. Standardizing effect size from linear regression models with log-transformed variables for meta-analysis. *BMC Med. Res. Methodol.* **17**, 44 (2017). 28302052 10.1186/s12874-017-0322-8 PMC5356327

[51] Hedges, L. V., Tipton, E. & Johnson, M. C. Robust variance estimation in meta-regression with dependent effect size estimates. *Res. Synth. Methods* **1**, 39–65 (2010). 26056092 10.1002/jrsm.5

[52] Tipton, E. Robust variance estimation in meta-regression with binary dependent effects. *Res. Synth. Methods* **4**, 169–187 (2013). 26053656 10.1002/jrsm.1070

[53] Deeks, J. J., Higgins, J. P., Altman, D. G., Joanne, M. E. & Veroniki, A. A. in *Cochrane Handbook for Systematic Reviews of Interventions* (eds Higgins J. P. T. et al.) Ch. 10 (2024); www.cochrane.org/handbook

[54] Bedoya, G., Coville, A., Haushofer, J., Isaqzadeh, M. & Shapiro, J. P. No household left behind: afghanistan targeting the ultra poor impact evaluation. *National Bureau of Economic Research*10.3386/w25981 (2019).

[55] Bedoya, G. et al. The enduring impacts of a big push during multiple crises: experimental evidence from Afghanistan. *World Bank Group*10.1596/1813-9450-10596 (2023).

[56] Tipton, E. Small sample adjustments for robust variance estimation with meta-regression. *Psychol. Methods* **20**, 375–393 (2015). 24773356 10.1037/met0000011

[57] Fisher, Z. & Tipton, E. robumeta: an R-package for robust variance estimation in meta-analysis. Preprint at https://arxiv.org/abs/1503.02220 (2015).

[58] Packheiser, J. et al. A systematic review and multivariate meta-analysis of the physical and mental health benefits of touch interventions. *Nat. Hum. Behav.* **8**, 1088–1107 (2024). 38589702 10.1038/s41562-024-01841-8 PMC11199149

[59] Nakagawa, S. et al. orchaRd 2.0: an R package for visualising meta-analyses with orchard plots. *Methods Ecol. Evol.* **14**, 2003–2010 (2023).

[60] Furuya-Kanamori, L., Barendregt, J. J. & Doi, S. A. R. A new improved graphical and quantitative method for detecting bias in meta-analysis. *Int. J. Evid. Based Healthc.* **16**, 195–203 (2018). 29621038 10.1097/XEB.0000000000000141

[61] Shamim, M. A., Dwivedi, P. & Padhi, B. K. Beyond the funnel plot: the advantages of Doi plots and prediction intervals in meta-analyses. *Asian J. Psychiatr.* **84**, 103550 (2023). 36958229 10.1016/j.ajp.2023.103550

[62] Harrer, M., Cuijpers, P., Furukawa, T. A. & Ebert, D. D. in *Doing Meta-Analysis with R: A Hands-On Guide* (eds Harrer, M. et al.) Ch. 9 (Chapman & Hall, 2022).

[63] Steinert, J. I. & Peterman, A. Project: Social safety nets, women’s economic achievements and agency: a systematic review and meta-analysis. *Open Science Framework*10.17605/OSF.IO/BRVY2 (2025). 10.1038/s41562-025-02394-0 PMC13121000 41644770

