# Supplementary material for: Social safety nets, women’s economic achievements and agency in 45 countries: a systematic review and meta-analysis
Source: Nat Hum Behav. 2026 Feb 5;10(4):698–714. doi: 10.1038/s41562-025-02394-0 (PMC13121000; doi:10.1038/s41562-025-02394-0)
Supplement: Supplementary file 1 — Supplementary Materials and Methods, Supplementary Figs. 1–6, Supplementary Tables 1–15 and supplementary works cited for included studies. [file 41562_2025_2394_MOESM1_ESM.pdf]

nature human behaviour

Supplementary information

https://doi.org/10.1038/s41562-025-02394-0

Social safety nets, women's economic achievements and agency in 45 countries: a systematic review and meta-analysis

In the format provided by the authors and unedited

Table of Contents

1. Figures 1
Fig. S1. Number of Included Publications Over Time.. 1
Fig. S2. Distribution of Intervention Duration and Follow-up Timing 2
Fig. S3. Cook's Distance (D) Values by Social Safety Net Type........ 3
Fig. S4. Orchard Plots for Pooled Effects by Domain. 4
Fig. S5. Orchard Plots for Pooled Effects by SSN type........ 5
Fig. S6. Doi Plot Assessing Publication Bias. 6

2. Tables 7
Table S1. Inclusion and Exclusion Criteria 9
Table S2. Publication Details by Country and Year. 10
Table S3. Pooled Effect Sizes for All Social Safety Net Interventions 18
Table S4. Pooled Effect Sizes Across All Outcomes by Different Social Safety Net Interventions. 19
Table S5. Pooled Effect Sizes for Economic Achievement and Agency Domains by Social Safety Net Intervention Types 20
Table S6. Pooled Effect Sizes Across All Social Safety Net Interventions by Different Outcomes 21
Table S7. Pooled Effect Sizes Across All Outcomes by Different Social Safety Net Interventions, Excluding Influential Effects.. 22
Table S8. Indicator Definitions for Covariates at the Publication, Intervention and Effect Levels. 24
Table S9. Summary of Cost-Benefit Analysis in Included Studies 28
Table S10. Summary of related meta-analyses on women's economic achievement and agency outcomes (2013 – 2023)........ 29
Table S11. Preferred Reporting Items for Systematic Reviews and Meta-Analyses (PRISMA) Checklist 32
Table S12. Preferred Reporting Items for Systematic Reviews and Meta-Analyses (PRISMA) Checklist for Abstracts........ 33
Table S13. Search String Primary Papers and Pilot Results 34
Table S14. Scopus Search String in English, Spanish and French. 40
Table S15. Revised Joanna Briggs Institute Quality Assessment for Experimental Studies.... 41

3. Works cited for included papers (alphabetical order) 42

1. Figures

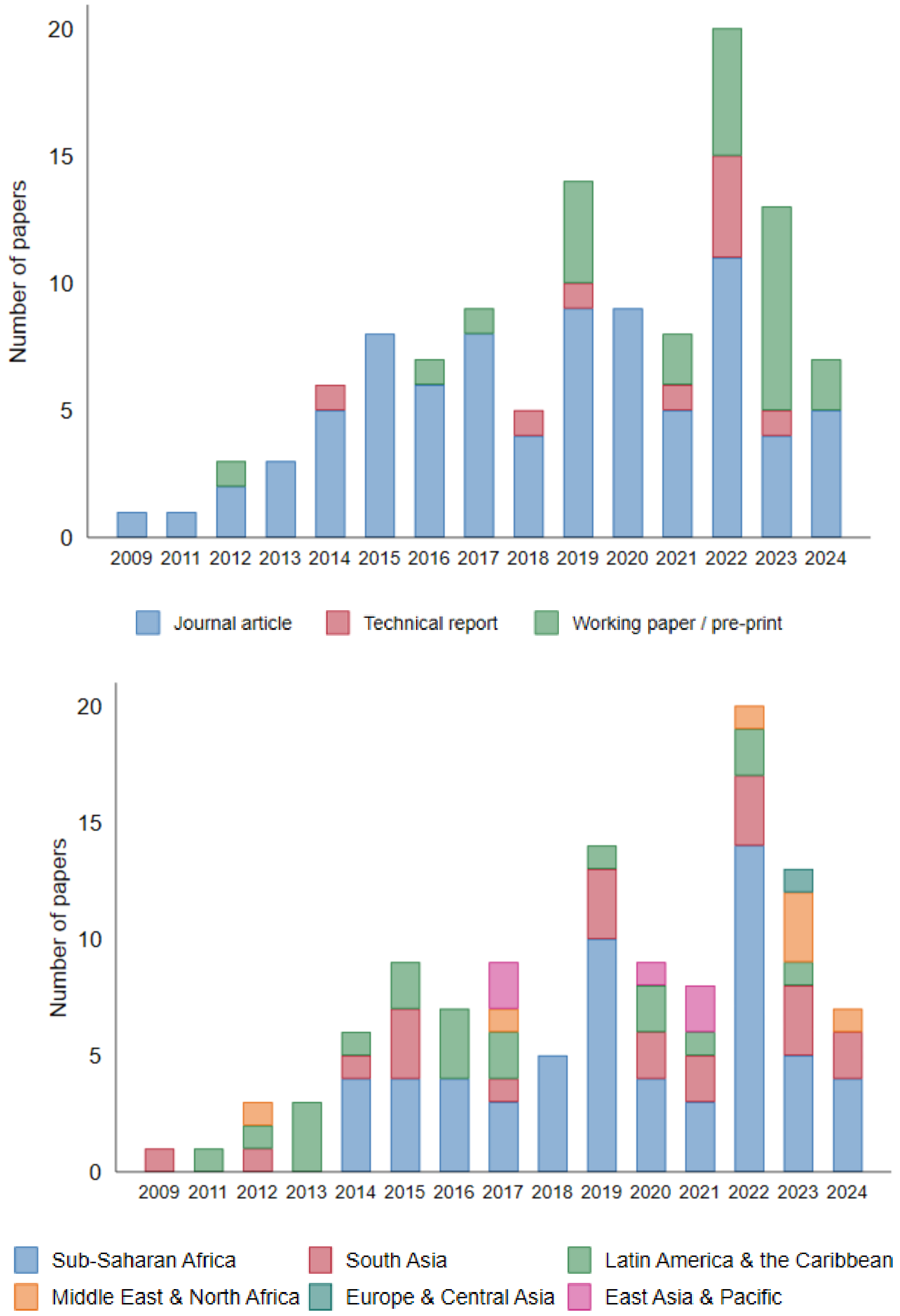

Fig. S1. Number of Included Publications Over Time. Top panel shows papers over time by type of publication (n = 115); Bottom panel shows papers over time by geographic region (n = 115).

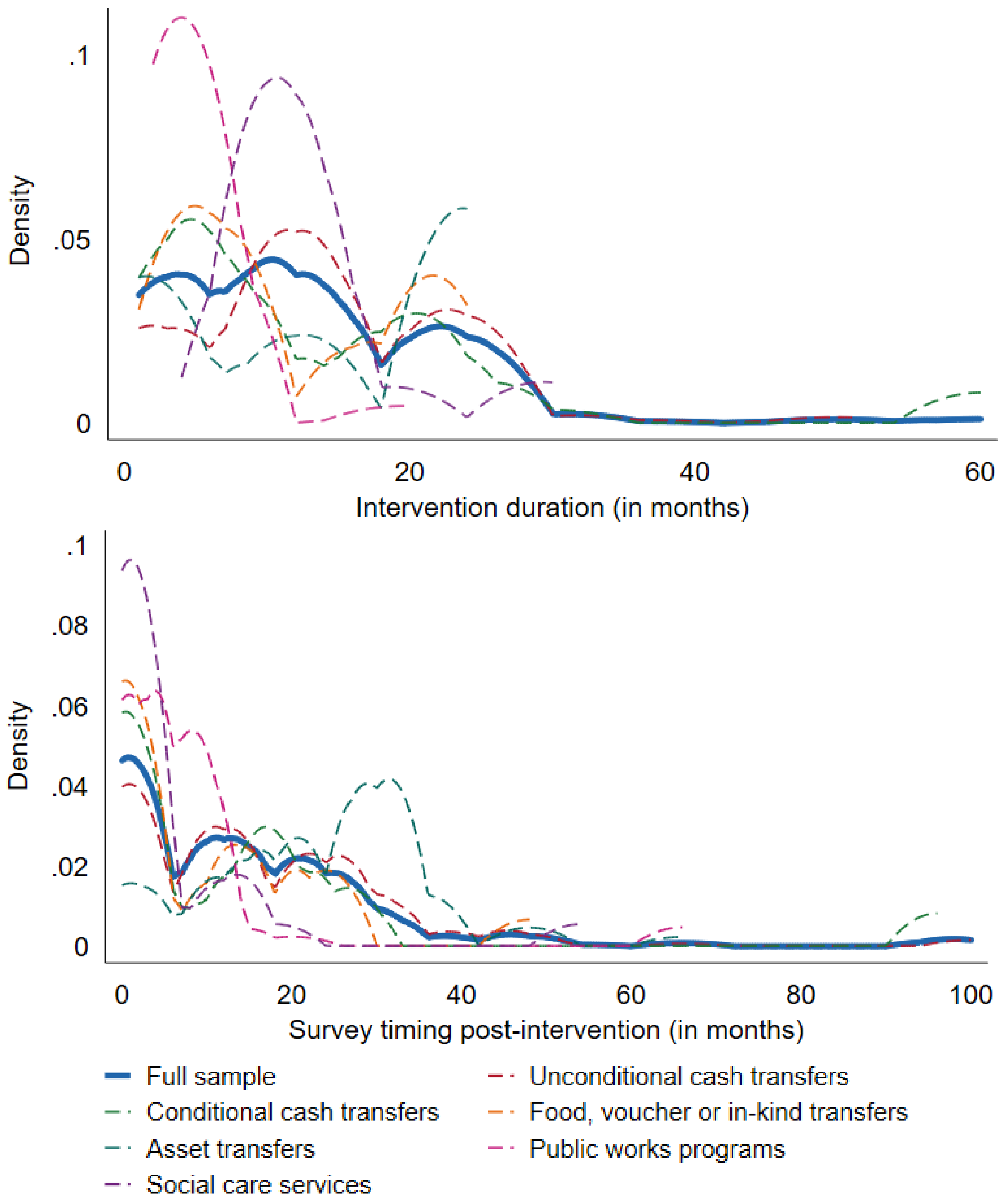

Fig. S2. Distribution of Intervention Duration and Follow-up Timing. Top panel shows density of effect-level intervention duration (in months), while bottom panel shows the effect-level distribution of the follow-up measurement (in months), truncated at 100 months (n = 1,307); Both figures show the primary SSN in the case an intervention had more than one and include a 2.7 kernel bandwidth.

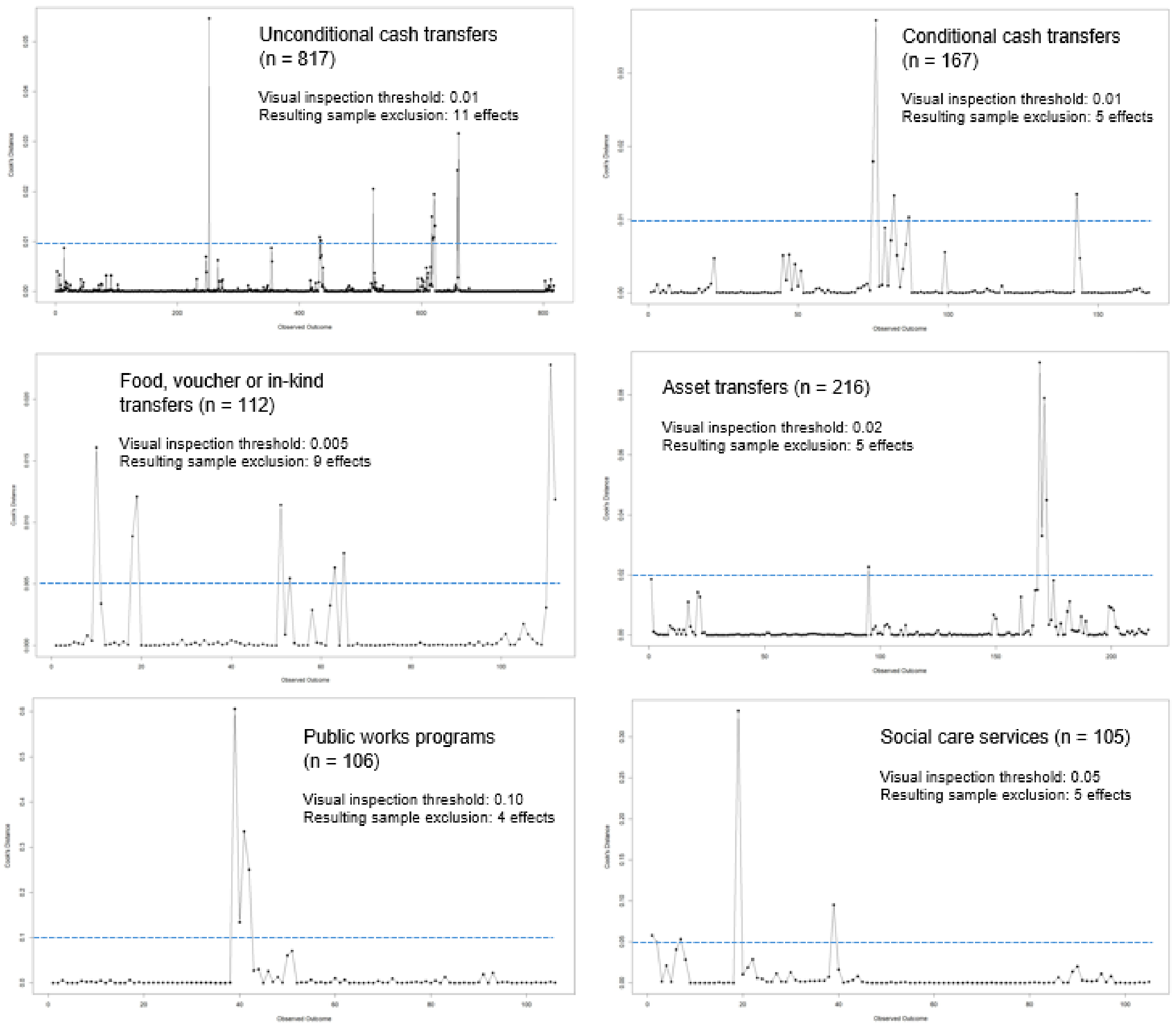

Fig. S3. Cook's Distance (D) Values by Social Safety Net Type. Cook's *D* is calculated based on the random-effects multi-level model and measures how much the estimated pooled effect size would change if an effect was dropped from the analysis. Blue lines represent visual inspection outlier thresholds and associated number of effects dropped in outlier sensitivity analyses.

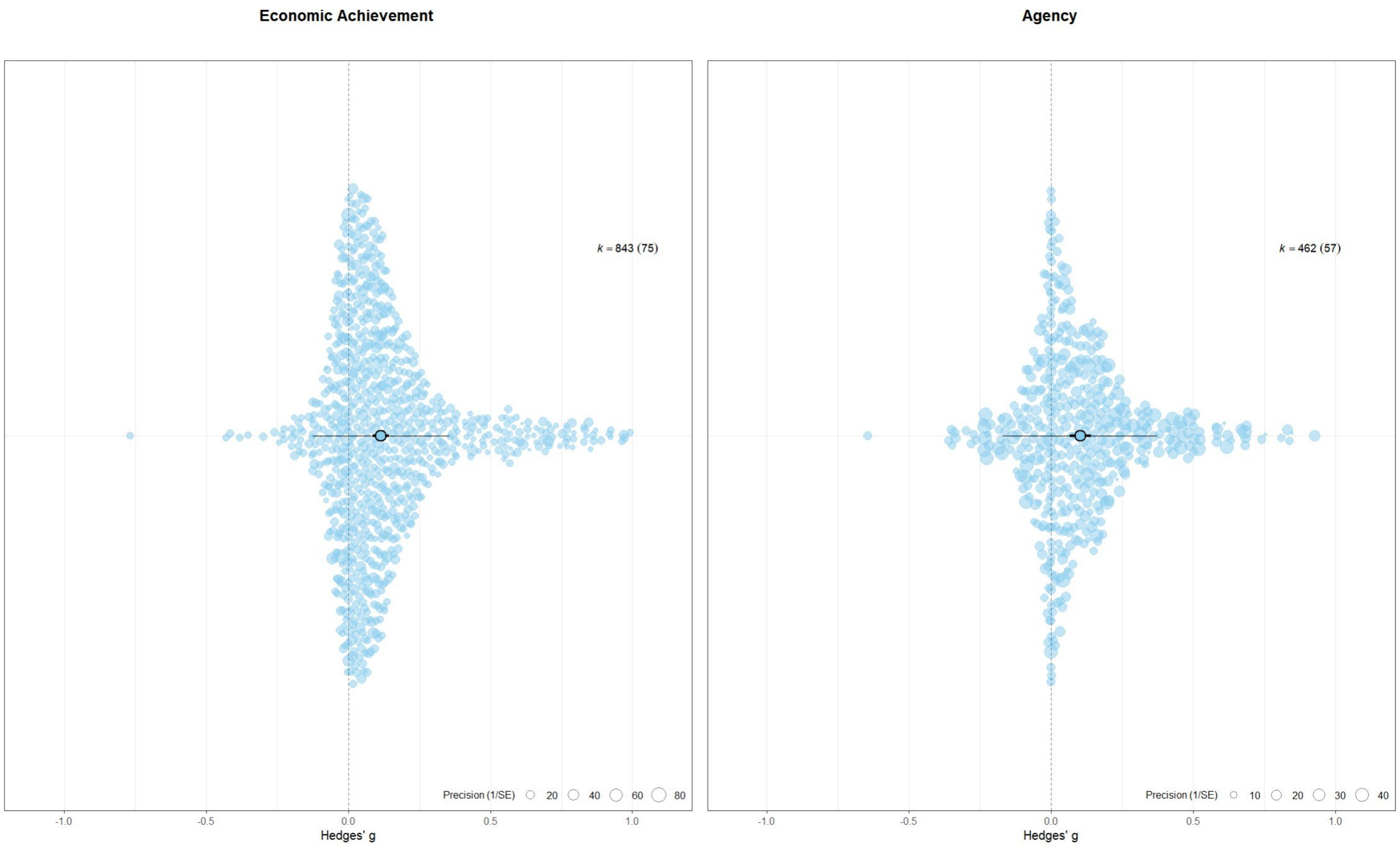

Fig. S4. Orchard Plots for Pooled Effects by Domain. Orchard plots visualizing pooled effects of SSNs on women's economic achievement (left panel) and agency (right panel) from multi-level random effects models with standard errors clustered at the study level: effect size (Hedges' g, circles), 95% confidence intervals (thick lines) and 95% prediction intervals (thin lines).

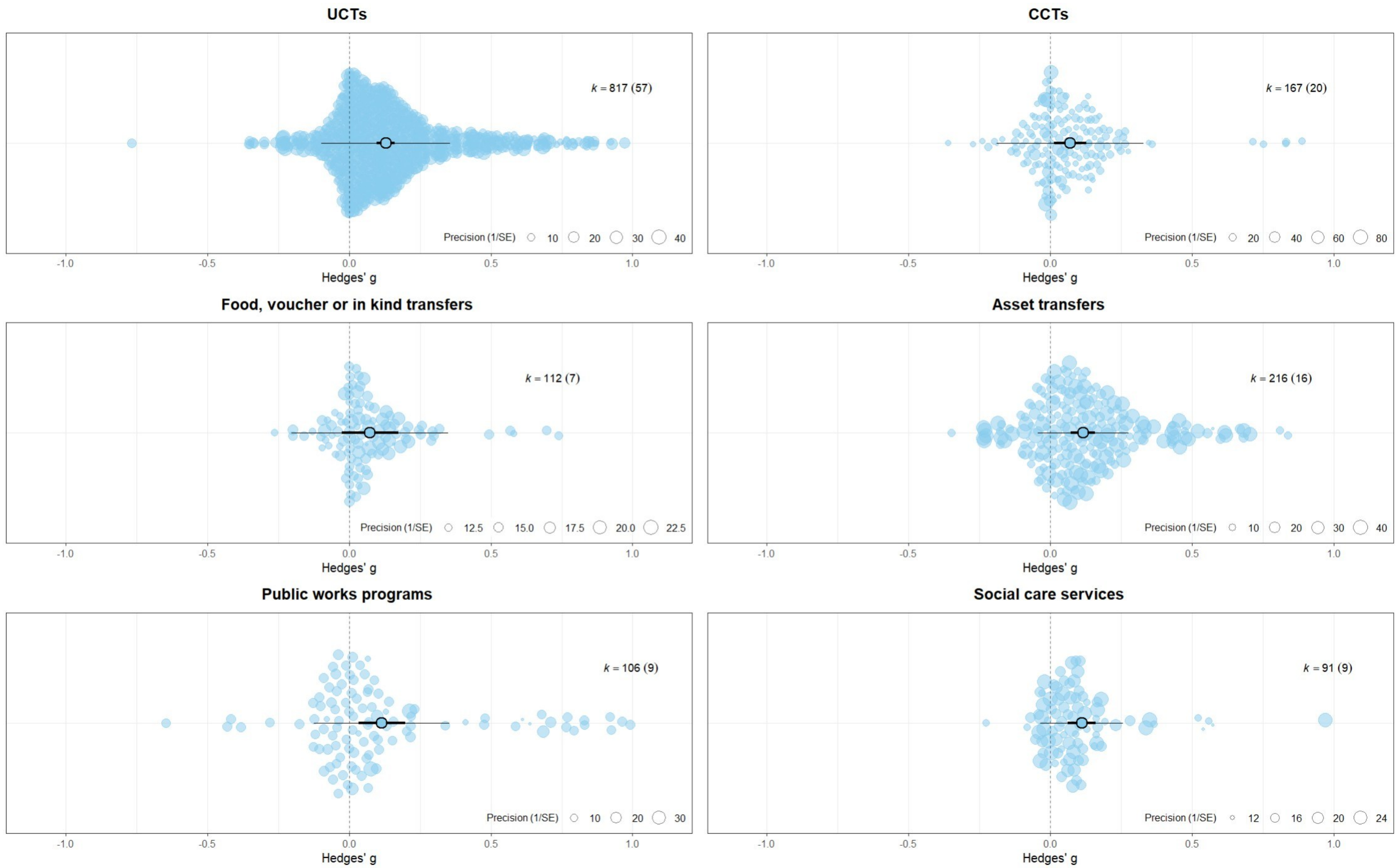

Fig. S5. Orchard Plots for Pooled Effects by SSN type. Orchard plots visualizing pooled effects of SSNs on women's economic achievement and agency by type of SSN from multi-level random effects models with standard errors clustered at the study level: effect size (Hedges' g, circles), 95% confidence intervals (thick lines) and 95% prediction intervals (thin lines).

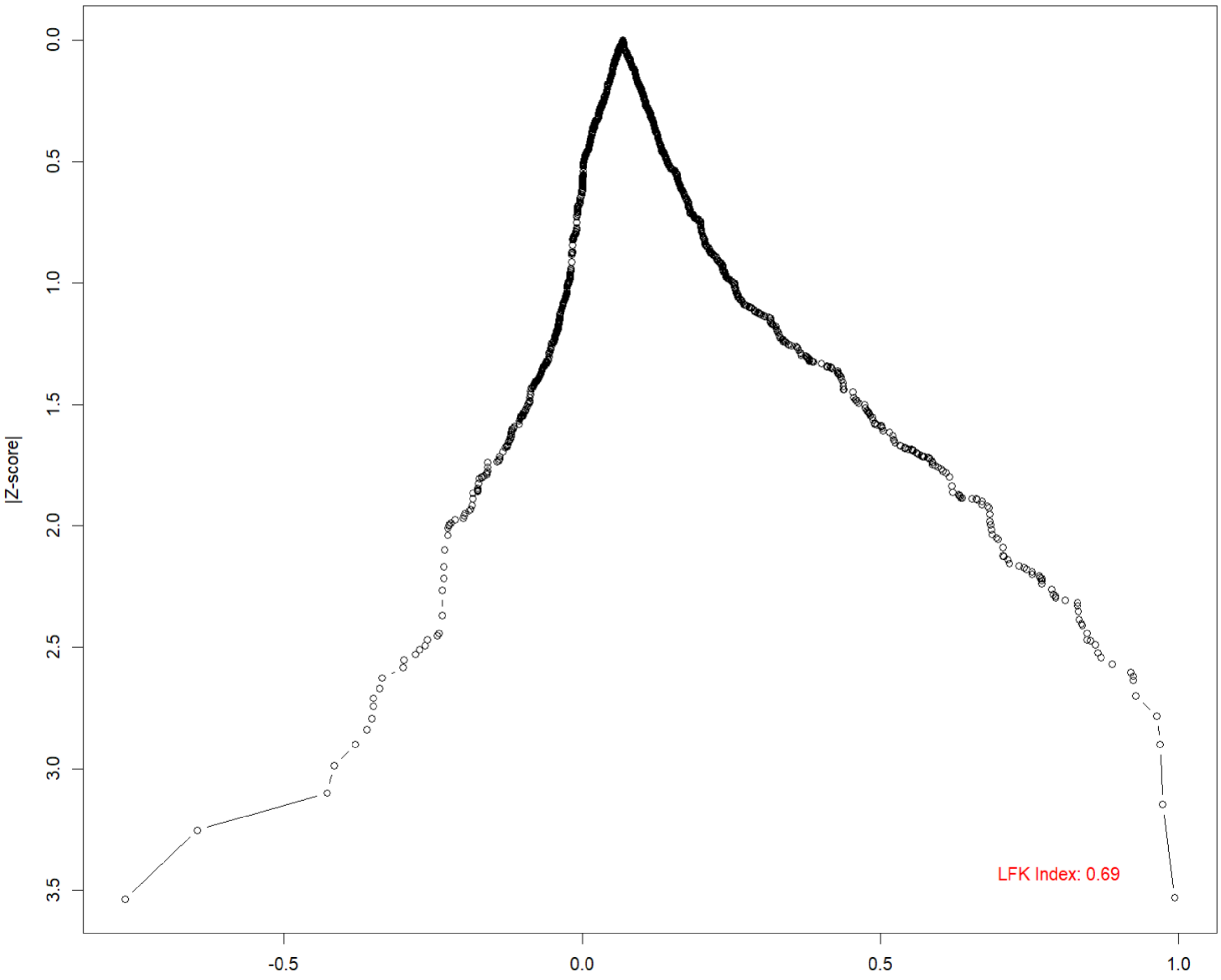

Fig. S6. Doi Plot Assessing Publication Bias. Analysis conducted using the full sample of effect sizes (n = 1,307); In the absence of publication bias, the Doi plot should be visually symmetrical and the value of the Luis Furuya-Kanamori (LFK) index should not lie outside the range of -1 to 1; calculated LFK index = 0.69, within the range of symmetry

2. Tables

Domain	Inclusion criteria	Exclusion criteria
Intervention	Social safety nets, or social assistance interventions, broadly aligned with the World Bank's Atlas of Social Protection Indicators of Resilience and Equity (*ASPIRE*) categorization for non-contributory programming as follows:<br>• **Unconditional cash transfers** (e.g., poverty-targeted cash transfers, family allowances, emergency cash support, non-contributory old-age social pensions, disability benefits)<br>• **Conditional cash transfers** (e.g., cash transfers including co-responsibilities or behavioral requirements which are monitored and enforced)<br>• **Food, vouchers or consumable in-kind transfers** (e.g., food stamps, take-home food rations, emergency food support, school feeding, in-kind supplies)<br>• **Productive asset transfers** (e.g., livestock, business equipment, materials or lumpy asset transfers, including graduation programs)<br>• **Public works** (e.g., cash-for-work, food-for-work, wage or job subsidies or direct job creation activities)<br>• **Fee waivers and targeted subsides** (e.g., health insurance exemptions, education waivers or scholarships, housing subsidies or allowances, utility or agricultural input subsides, transportation benefits)<br>• **Social care services** (e.g., non-cash family support services, including childcare, eldercare, care for people with disabilities, child protection services – as long as the intervention conveyed an economic benefit – including vouchers, free access, subsidized care etc.)<br><br>Interventions may be evaluated as stand-alone programming, bundled together with multiple social safety nets, or bundled with additional 'plus programming' of various forms (e.g., livelihood or nutrition trainings, parenting programs)	• Evaluations of social protection or job market interventions beyond social safety nets, including micro-finance interventions, savings interventions, mentoring or skills training interventions, interventions which are purely with educational or early childhood development objectives<br>• Evaluations which evaluate a 'plus' component only, layered over a social safety net<br>• Evaluations which compare two equally valued social safety nets (e.g., cash vs. food transfers with no control group or cash transfers to men vs. women with no control group)<br>• Evaluations which compare a dosage effect of the same social safety net intervention without a clear exposure period for different groups
Setting	Low- and middle-income countries	High-income countries

Population	Impacts reported for adult women (18 years or older) or samples consisting of 95% women, or mixed female ages which have an overall mean sample age of 18 or over, regardless of who reports on outcomes	Impacts reported exclusively for men, in populations younger than 18 years, in a mixed sample of men and women without disaggregation (exception is a mixed sample when there are ≤ 5% men in the total sample), or where the overall mean sample age is younger than 18 years
Outcomes	**Economic achievement**: Following the categories and terminology in the CGD measuring women's economic empowerment compendium:<br>• **Labor force participation** (extensive margin, e.g., any formal or informal employment or work-for-pay, any own farm or self-employment non-farm work, any business operation)<br>• **Productive work intensity or quality of work** (intensive margin, e.g., number of hours worked, wage, profits, revenue, earnings and income, or quality of work, such as "receives benefits" or "flexible work hours" or "daytime shifts")<br>• **Unpaid care work** (extensive margin, e.g., any unpaid care work, use/uptake of formal or informal care services)<br>• **Unpaid care work intensity or quality of work** (e.g., number of hours worked in care or domestic tasks, quality of activities)<br>• **Savings** (e.g., any or amount saved, use of financial services for savings)<br>• **Debt or loans** (e.g., any or amount outstanding owed, use of financial services for credit, loans)<br>• **Assets** (e.g., durable or productive asset ownership including livestock, business assets, information technology)<br>• **Expenditure** (e.g., personal expenditure on durable goods or investment in productive activities)<br>**Agency**: Following the conceptualization by the Bill and Melinda Gates Foundation's Conceptual Model of Empowerment focused on "choice" "voice" and "leadership":<br>• **Decision making** (e.g., household and individual sole or joint decision-making and bargaining power)<br>• **Autonomy and self-efficacy** (e.g., self-efficacy, power, control, mobility, independence, self-worth and value, confidence)<br>• **Aspirations and goals** (e.g., stated aspirations related to economic or agency outcomes)<br>• **Voice** (e.g., ability to speak out, collective agency, voting, membership and participation in groups)	• Measures labeled as economic achievement or agency which are unclear in terms of definition<br>• Measures of economic achievement or agency which are hypothetical or expected, rather than realized (e.g., expected wage, reservation wage or decision-making outcome respondent would accept)<br>• Aggregate indices which are composed of majority indicators outside economic achievement or agency domains

	• **Leadership** (e.g., leadership positions, participation in local governance)<br>Aggregates in either economic or agency domain that include multiple measures	
Methodology	Quantitative evaluations using experimental designs: individual or cluster-randomized control trials reporting intent-to-treat (ITT) estimates	• Evaluations reporting only treatment-on-the-treated (TOT) or per-protocol analyses<br>• Quantitative evaluations using quasi-experimental or non-experimental methods<br>• Qualitative evaluations
Time frame	Studies published from 2003 to 2024	Studies published before 2003
Type of publication	Journal article, working or discussion paper, pre-print, technical report	Policy brief, presentation or other outputs with insufficient information to determine eligibility criteria and methodological details
Language	Studies in English, French or Spanish	Studies in all other languages

Table S1. Inclusion and Exclusion Criteria.

ID	Authors	Type	Year	Country	Intervention	Type(s) of SSN	Included impacts			Quality assessment
							Total	Economic	Agency	
58	Bedoya et al.	†	2019	Afghanistan	Targeting the Ultra Poor program	Asset transfer; UCT; Fee waiver or subsidy	13	5	8	90%
58	Bedoya et al.	†	2023	Afghanistan	Targeting the Ultra Poor program	Asset transfer; UCT; Fee waiver or subsidy	8	4	4	90%
70	Gibbs et al.	*	2020	Afghanistan	Women for Women International	UCT	4	2	2	90%
92	Kashefi & Naito	*	2023	Afghanistan	Promoting Entrepreneurship among Youth in Afghanistan	UCT	10	10	0	80%
17	Roy et al.	*	2015	Bangladesh	BRAC's Targeting the Ultra Poor (TUP) Program	Asset transfer; UCT	66	19	47	80%
17	Bandiera et al.	*	2017	Bangladesh	BRAC's Targeting the Ultra Poor (TUP) Program	Asset transfer; UCT	6	6	0	80%
57	Roy et al.	*	2019	Bangladesh	The Transfer Modality Research Initiative	UCT; Food, voucher or in-kind transfer	20	6	14	90%
57	Roy et al.	*	2024	Bangladesh	The Transfer Modality Research Initiative	UCT; Food, voucher or in-kind transfer	12	4	8	90%
57	Ahmed et al.	*	2023	Bangladesh	The Transfer Modality Research Initiative	UCT; Food, voucher or in-kind transfer	8	8	0	90%
71	Hussam et al.	*	2022	Bangladesh	NR	Public works; UCT	10	4	6	80%
72	Hossain et al.	*	2022	Bangladesh	NR	UCT	2	0	2	90%
73	Karasz et al.	*	2021	Bangladesh	ASHA (Hope) project	UCT	5	0	5	90%
93	Rahman et al.	*	2021	Bangladesh	Ultra-Poor Graduation program	UCT	5	5	0	70%
36	Attanasio et al.	†	2022	Brazil	Rio de Janeiro's public daycare program	Social care services	8	8	0	88%
18	Karimli et al.	*	2020	Burkina Faso	Trickle Up and Trickle Up plus	UCT	46	46	0	90%
18	Ismayilova et al.	*	2018	Burkina Faso	Trickle Up and Trickle Up plus	UCT	4	0	2	90%

Table S2. Publication Details by Country and Year. * = journal article, † = working paper or pre-print, § = technical report. CCT = conditional cash transfer, NR = not reported, UCT = unconditional cash transfer; Quality assessment is the percentage of 'yes' answers among those applicable by study using a modified version of the Joanna Briggs Institute assessment tool for experimental studies (see Table S15).

ID	Authors	Type	Year	Country	Intervention	Type(s) of SSN	Included impacts			Quality assessment
							Total	Economic	Agency	
31	Ajayi et al.	†	2022	Burkina Faso	Youth Employment and Skills Project + Mobile Creches	Social care services	12	9	3	80%
75	Olney et al.	*	2016	Burkina Faso	3-y enhanced-homestead food production program	Asset transfer	5	0	5	70%
75	van den Bold et al.	*	2015	Burkina Faso	3-y enhanced-homestead food production program	Asset transfer	9	9	0	70%
15	Martizez & Perticara	*	2017	Chile	Chile's 4–7 Program	Social care services	10	10	0	90%
43	Attanasio et al.	*	2011	Colombia	Jóvenes en Acción	CCT	9	9	0	80%
33	Gazeaud et al.	§	2022	Comoros	The Social Safety Net Project	Public works	3	2	1	90%
30	Angelucci et al.	*	2023	DRC	Stronger Nations Stronger Women	UCT	27	22	5	100%
38	Donald & Vaillant	†	2023	DRC	NR	Social care services	31	28	3	70%
87	Devoto et al.	†	2024	Djibouti	Djiboutian Public Works Program	Public works	30	27	3	90%
63	Hidrobo & Fernald	*	2012	Ecuador	Bono de Desarrollo Humano	UCT	2	0	2	90%
77	Peterman et al.	*	2021	Ecuador	World Food Programme's food, cash & voucher program	Food, voucher or in-kind transfer; CCT	9	0	9	90%
77	Buller et al.	*	2016	Ecuador	World Food Programme's food, cash & voucher program	Food, voucher or in-kind transfer; CCT	8	0	8	90%
77	Hidrobo et al.	*	2016	Ecuador	World Food Programme's food, cash & voucher program	Food, voucher or in-kind transfer; CCT	5	3	2	90%
6	Caria et al.	§	2022	Egypt	NR	Social care services	2	2	0	80%
65	Crepon et al.	*	2023	Egypt	NR	Food, voucher or in-kind transfer; UCT	26	24	2	70%
89	Christian et al.	§	2023	El Salvador	Food Assistance for Assets Program (FFA) for Women	Public works; UCT	34	26	8	70%

Table S2. Publication Details by Country and Year (continued, 2). * = journal article, † = working paper or pre-print, § = technical report. CCT = conditional cash transfer, NR = not reported, UCT = unconditional cash transfer; Quality assessment is the percentage of 'yes' answers among those applicable by study using a modified version of the Joanna Briggs Institute assessment tool for experimental studies (see Table S15).

ID	Authors	Type	Year	Country	Intervention	Type(s) of SSN	Included impacts			Quality assessment
							Total	Economic	Agency	
19	Alderman et al.	§	2021	Ethiopia	Strengthen PSNP4 Institutions and Resilience (SPIR) Development Food Security Activity (DFSA)	UCT; Asset transfer	20	20	0	90%
19	Ranganathan et al.	*	2022	Ethiopia	Strengthen PSNP4 Institutions and Resilience (SPIR) Development Food Security Activity (DFSA)	UCT; Asset transfer	10	0	10	90%
44	Banerjee et al.	*	2015	Ethiopia	Graduation Program	Asset transfer	2	0	2	90%
11	Fafchamps et al.	*	2014	Ghana	NR	UCT; Asset transfer	4	4	0	90%
45	Banerjee et al.	*	2015	Ghana	Graduation Program	Asset transfer; UCT	4	2	2	90%
45	Banerjee et al.	*	2022	Ghana	Graduating from Ultra Poverty ("GUP")	Asset transfer	11	7	4	90%
46	Banerjee et al.	*	2015	Honduras	Graduation Program	Asset transfer; Food, voucher or in-kind transfer	2	0	2	90%
82	Alzua et al.	*	2013	Honduras	Programa de Asignación Familiar ("Family Allowance Program") (PRAF)	CCT	3	3	0	70%
82	Molina Millán et al.	*	2019	Honduras	Programa de Asignación Familiar ("Family Allowance Program") (PRAF)	CCT	11	11	0	70%
83	Benedetti et al.	*	2016	Honduras	Bono 10,000	CCT	2	2	0	80%
29	Nandi et al.	*	2020	India	Uttam Unnati ('great progress')	Social care services	7	7	0	80%
47	Banerjee et al.	*	2015	India	Graduation Program	Asset transfer; UCT	1	0	1	90%
56	Banerjee et al.	*	2017	Indonesia	Program Keluarga Harapan (PKH)	CCT	1	1	0	60%
88	Wolf et al.	*	2024	Ivory Coast	100WEEKS Program	UCT	1	1	0	70%

Table S2. Publication Details by Country and Year (continued, 3). * = journal article, † = working paper or pre-print, § = technical report. CCT = conditional cash transfer, NR = not reported, UCT = unconditional cash transfer; Quality assessment is the percentage of 'yes' answers among those applicable by study using a modified version of the Joanna Briggs Institute assessment tool for experimental studies (see Table S15).

ID	Authors	Type	Year	Country	Intervention	Type(s) of SSN	Included impacts: Total	Economic	Agency	Quality assessment
8	Groh et al.	†	2012	Jordan	Jordan New Opportunities for Women (Jordan NOW) pilot	Public works	38	32	6	90%
2	Clark et al.	*	2019	Kenya	NR	Social care services	6	5	1	70%
3	Brooks et al.	*	2022	Kenya	NR	UCT	5	5	0	80%
7	Haushofer & Shapiro	*	2016	Kenya	GiveDirectly	UCT	4	0	4	90%
7	Haushofer et al.	†	2019	Kenya	GiveDirectly	UCT	2	2	0	90%
21	Gobin et al.	*	2017	Kenya	Rural Entrepreneur Access Program	UCT	3	3	0	70%
26	Brudevold-Newman et al.	†	2017	Kenya	NR	UCT; Asset transfer	46	36	10	90%
28	Asfaw et al.	*	2014	Kenya	The Kenya Cash Transfer Programme for Orphans and Vulnerable Children	UCT	4	4	0	80%
37	Orkin et al.	†	2023	Kenya	NR	UCT	2	0	2	90%
59	Gallardo et al.	§	2022	Kenya	Development Impact Bond	CCT	1	1	0	70%
41	Perova et al.	†	2021	Lao People’s Democratic Republic	Road Management Group Program	Public works	1	0	1	70%
12	Pace et al.	*	2019	Lesotho	Child Grants Program	UCT	1	1	0	80%
12	Daidone et al.	*	2019	Lesotho	Lesotho Child Grant Program	UCT	9	9	0	80%
1	Baird et al.	*	2019	Malawi	Zomba Cash Transfer Program	UCT; CCT	24	15	9	70%
67	Ambler et al.	†	2019	Malawi	NR	UCT; Food, voucher or in-kind transfer	61	0	61	70%
76	Angeles et al.	*	2019	Malawi	Malawi Social Cash Transfer	UCT	2	2	0	90%

Table S2. Publication Details by Country and Year (continued, 4). * = journal article, † = working paper or pre-print, § = technical report. CCT = conditional cash transfer, NR = not reported, UCT = unconditional cash transfer; Quality assessment is the percentage of ‘yes’ answers among those applicable by study using a modified version of the Joanna Briggs Institute assessment tool for experimental studies (see Table S15).

ID	Authors	Type	Year	Country	Intervention	Type(s) of SSN	Included impacts: Total	Economic	Agency	Quality assessment
76	Lambon-Quayefio et al.	*	2024	Malawi	Malawi Social Cash Transfer	UCT	4	4	0	90%
90	Bedi et al.	†	2023	Malawi	Multifaceted Anti-poverty Program for the Ultra Poor	UCT	123	99	24	70%
24	Heath et al.	*	2020	Mali	*Programme de Filets Sociaux (Jigisemejiri)*	UCT	5	2	3	70%
42	Aguila & Smith	*	2020	Mexico	*Reconocer Urbano*	UCT	6	6	0	80%
54	Banerjee et al.	*	2017	Mexico	*Programa de Apoyo Alimentario (PAL)*	UCT	4	4	0	60%
81	Alzua et al.	*	2013	Mexico	*Programa de Educacion, Salud y Alimentacion*	CCT	7	7	0	70%
81	Urbina	*	2020	Mexico	*Programa de Educacion, Salud y Alimentacion*	CCT	3	0	3	70%
69	Field & Maffioli	†	2021	Myanmar	National maternal cash transfer pilot	UCT	10	0	10	88%
13	Janzen et al.	†	2023	Nepal	Heifer International's livestock transfer program	Asset transfer; UCT	20	8	12	80%
16	Gram et al.	*	2019	Nepal	The Low Birth Weight South Asia Trial (LBW-SAT)	UCT; Food, voucher or in-kind transfer	32	0	32	70%
16	Harris-Fry et al.	*	2022	Nepal	The Low Birth Weight South Asia Trial (LBW-SAT)	UCT; Food, voucher or in-kind transfer	2	0	2	70%
32	Hojman & Boo	*	2022	Nicaragua	*Programa Urbano*	Social care services	1	1	0	60%
52	Alzua et al.	*	2013	Nicaragua	*Red de Protección Social* ("Social Protection Network")	CCT	6	6	0	80%
64	Macours & Vakis	*	2014	Nicaragua	*Atención a Crisis*	CCT; UCT	15	0	15	90%
39	Bossuroy el al.	*	2022	Niger	Niger national cash transfer program	UCT	44	28	16	90%

Table S2. Publication Details by Country and Year (continued, 5). * = journal article, † = working paper or pre-print, § = technical report. CCT = conditional cash transfer, NR = not reported, UCT = unconditional cash transfer; Quality assessment is the percentage of 'yes' answers among those applicable by study using a modified version of the Joanna Briggs Institute assessment tool for experimental studies (see Table S15).

ID	Authors	Type	Year	Country	Intervention	Type(s) of SSN	Included impacts: Total	Economic	Agency	Quality assessment
34	Carneiro et al.	§	2019	Nigeria	Child Development Grant Programme	UCT	10	10	0	100%
62	Bakhtiar et al.	†	2024	Nigeria	Feed the Future Nigeria Livelihoods Project	UCT	21	0	21	60%
50	Armand et al.	†	2023	North Macedonia	the Subsidized Employment Program	Public works	2	2	0	90%
48	Banerjee et al.	*	2015	Pakistan	Graduation Program	Asset transfer; UCT	2	0	2	90%
49	Banerjee et al.	*	2015	Peru	Graduation Program	Asset transfer; UCT	2	0	2	90%
55	Banerjee et al.	*	2017	Philippines	*Pantawid Pamilya Program*	CCT	4	4	0	60%
66	Edmonds & Theoharides	*	2020	Philippines	*Kabuhayan Para sa Magulang ng Batang Manggagawa (KASAMA)*	Asset transfer	5	5	0	100%
68	Ambler et al.	†	2019	Senegal	NR	UCT	18	0	18	70%
74	Rosas & Sabarwal	†	2016	Sierra Leone	Cash for Work Program	Public works	2	2	0	60%
91	Rosas et al.	*	2022	Sierra Leone	Cash plus Training Program for Youth	CCT	7	6	1	90%
23	Abdullahi et al.	*	2022	Somalia	Humanitarian Support and Re-Integration of IDP and Returnees in Mogadishu & Building Resilient Communities in Somalia	UCT	12	12	0	80%
5	Kilburn et al.	*	2018	South Africa	HIV Prevention Trial Network 068	CCT	2	0	2	80%
5	Kilburn et al.	*	2019	South Africa	HIV Prevention Trial Network 068	CCT	4	4	0	80%

Table S2. Publication Details by Country and Year (continued, 6). * = journal article, † = working paper or pre-print, § = technical report. CCT = conditional cash transfer, NR = not reported, UCT = unconditional cash transfer; Quality assessment is the percentage of 'yes' answers among those applicable by study using a modified version of the Joanna Briggs Institute assessment tool for experimental studies (see Table S15).

ID	Authors	Type	Year	Country	Intervention	Type(s) of SSN	Included impacts			Quality assessment
							Total	Economic	Agency	
40	de Mel et al.	*	2012	Sri Lanka	NR	UCT; Asset transfer	16	16	0	90%
51	de Mel et al.	*	2009	Sri Lanka	NR	UCT; Asset transfer	4	4	0	90%
84	de Mel et al.	*	2014	Sri Lanka	Start-and-Improve Your Business program	CCT	16	16	0	90%
9	Calderone et al.	†	2022	Tanzania	STRYDE 2.0	CCT	20	15	5	90%
22	Kuringe et al.	*	2022	Tanzania	Determined, Resilient, Empowered, AIDS-free, Mentored and Safe (DREAMS) initiative	UCT	1	1	0	60%
25	Tanzania cash plus evaluation team	§	2018	Tanzania	Productive Social Safety Net	CCT; UCT	5	3	2	70%
27	Palermo et al.	*	2021	Tanzania	Productive Social Safety Net	CCT	4	2	2	90%
20	Briaux et al.	*	2020	Togo	Pilot Cash Transfer Program	UCT	2	0	2	100%
14	Gazeaud et al.	*	2023	Tunisia	Cash Grant Study	UCT	16	14	2	90%
86	Leight & Mvukiyehe	†	2023	Tunisia	Community Works and Local Participation Project	Public works	8	4	4	71%
10	Blattman et al.	*	2014	Uganda	Youth Opportunities Program	UCT	6	4	2	90%
10	Blattman et al.	*	2020	Uganda	Youth Opportunities Program	UCT	7	7	0	90%
10	Fiala et al.	†	2022	Uganda	Youth Opportunities Program	UCT	6	6	0	90%
35	Bjorvatn et al.	†	2022	Uganda	NR	Social care services; UCT	22	22	0	90%
60	Gallardo et al.	§	2022	Uganda	Development Impact Bond	CCT	1	1	0	70%

Table S2. Publication Details by Country and Year (continued, 7). * = journal article, † = working paper or pre-print, § = technical report. CCT = conditional cash transfer, NR = not reported, UCT = unconditional cash transfer; Quality assessment is the percentage of 'yes' answers among those applicable by study using a modified version of the Joanna Briggs Institute assessment tool for experimental studies (see Table S15).

ID	Authors	Type	Year	Country	Intervention	Type(s) of SSN	Included impacts			Quality assessment
							Total	Economic	Agency	
61	Blattman et al.	*	2016	Uganda	Women's INcome Generating Support	CCT	4	0	4	90%
61	Green et al.	*	2015	Uganda	Women's INcome Generating Support	CCT	6	6	0	90%
78	Peterman et al.	*	2021	Uganda	World Food Programme	Food, voucher or in-kind transfer; UCT	4	0	4	90%
85	Gupta et al.	*	2023	Uganda	GiveDirectly	UCT	1	1	0	70%
4	Bonilla et al.	*	2017	Zambia	Zambia's Child Grant Program	UCT	2	0	2	90%
4	Handa et al.	*	2018	Zambia	Zambia's Child Grant Program	UCT	4	4	0	90%
79	Botea et al.	†	2023	Zambia	Supporting Women's Livelihoods Prorgram	UCT	32	28	4	80%
80	Handa et al.	*	2018	Zambia	Multiple Category Targeting Grant	UCT	4	4	0	100%
80	AIR et al.	§	2014	Zambia	Multiple Category Targeting Grant	UCT	2	0	2	100%

Table S2. Publication Details by Country and Year (continued, 8). * = journal article, † = working paper or pre-print, § = technical report. CCT = conditional cash transfer, NR = not reported, UCT = unconditional cash transfer; Quality assessment is the percentage of 'yes' answers among those applicable by study using a modified version of the Joanna Briggs Institute assessment tool for experimental studies (see Table S15).

	**All outcomes**	**Economic achievement**	**Agency**
	(1)	(2)	(3)
**Hedges' g**	**0.107*** **	**0.113*** **	**0.101*** **
**(SE)**	(0.011)	(0.014)	(0.020)
**95% CI**	[0.085, 0.129]	[0.085, 0.142]	[0.063, 0.139]
I^2 (consistency)	91.92%	92.53%	94.54%
τ^2 (heterogeneity)	0.025	0.025	0.042
**N of studies**	93	75	57
**N of effect sizes**	1307	843	462

Table S3. Pooled Effect Sizes for All Social Safety Net Interventions. Average pooled effect sizes are calculated using standardized impacts from robust variance estimation and reported with standard errors (SEs) and 95% confidence intervals (CIs); All statistical tests reported are two-sided; *p<0.05, **p<0.01, ***p<0.001.

	UCT	CCT	Asset transfers	In-kind transfers	Public work programs	Social care services
	(1)	(2)	(3)	(4)	(5)	(6)
**Hedges' g**	**0.128*** **	**0.059***	**0.115*** **	**0.071**	**0.127***	**0.122*** **
**(SE)**	(0.015)	(0.023)	(0.021)	(0.040)	(0.048)	(0.023)
**95% CI**	[0.097, 0.159]	[0.011, 0.108]	[0.071, 0.160]	[-0.026, 0.169]	[0.015, 0.239]	[0.071, 0.174]
I^2 (consistency)	91.93%	89.43%	90.01%	82.15%	94.56%	93.15%
τ^2 (heterogeneity)	0.033	0.011	0.032	0.019	0.062	0.034
**N of studies**	57	20	16	7	9	10
**N of effect sizes**	817	167	216	112	106	105

Table S4. Pooled Effect Sizes Across All Outcomes by Different Social Safety Net Interventions. Average pooled effect sizes are calculated using standardized impacts from robust variance estimation and reported with standard errors (SEs) and 95% confidence intervals (CIs); All statistical tests reported are two-sided; CCT = conditional cash transfer; UCT = unconditional cash transfer. *p<0.05, **p<0.01, ***p<0.001.

	UCT	CCT	Asset transfers	In-kind transfers	Public works	Social care
Panel A: Economic achievement outcomes						
**Hedges' G**	**0.122*** **	**0.056**	**0.127*** **	*Insufficient*	**0.178***	**0.127*** **
**(SE)**	(0.016)	(0.035)	(0.025)	*power*	(0.057)	(0.026)
**95% CI**	[0.089, 0.155]	[-0.018, 0.131]	[0.072, 0.183]		[0.042, 0.313]	[0.067, 0.188]
I^2 (consistency)	89.76%	92.05%	90.12%		95.54%	93.54%
τ^2 (heterogeneity)	0.024	0.015	0.038		0.076	0.035
**N of studies**	42	19	11	3	8	9
**N of effect sizes**	507	114	115	28	84	84
Panel B: Agency outcomes						
**Hedges' G**	**0.145*** **	**0.072**	**0.113*** **	**0.046*** **	**0.011**	*Insufficient*
**(SE)**	(0.026)	(0.038)	(0.023)	(0.012)	(0.035)	*power*
**95% CI**	[0.093, 0.197]	[-0.015, 0.159]	[0.063, 0.163]	[0.016, 0.076]	[-0.076, 0.097]	
I^2 (consistency)	95.12%	86.64%	92.73%	58.38%	80.48%	
τ^2 (heterogeneity)	0.062	0.017	0.037	0.006	0.018	
**N of studies**	39	10	12	7	7	3
**N of effect sizes**	308	53	101	84	22	7

Table S5. Pooled Effect Sizes for Economic Achievement and Agency Domains by Social Safety Net Intervention Types. Average pooled effect sizes are calculated using standardized impacts from robust variance estimation and reported with standard errors (SEs) and 95% confidence intervals (CIs); All statistical tests reported are two-sided; CCT = conditional cash transfer; UCT = unconditional cash transfer. *p<0.05, **p<0.01, ***p<0.001.

	Labor force participation	Productive work intensity	Care work participation	Care work intensity	Savings	Debt or loans	Assets	Expenditures
	(1)	(2)	(3)	(4)	(5)	(6)	(7)	(8)
**Hedges' g**	**0.106*** **	**0.075*** **	*Insufficient*	**-0.015**	**0.229*** **	**0.105**	**0.235*** **	**0.177** **
**(SE)**	(0.024)	(0.016)	*power*	(0.020)	(0.039)	(0.081)	(0.052)	(0.050)
**95% CI**	[0.058, 0.154]	[0.044, 0.106]		[-0.058, 0.028]	[0.147, 0.311]	[-0.082, 0.291]	[0.125, 0.345]	[0.072, 0.282]
I^2 (consistency)	92.95%	86.35%		80.01%	93.78%	93.57%	93.74%	93.14%
τ^2 (heterogeneity)	0.019	0.014		0.009	0.033	0.042	0.061	0.035
**N of studies**	44	52	5	16	21	9	16	19
**N of effect sizes**	155	370	12	62	75	21	83	55

	Decision-making	Autonomy and self-efficacy	Aspirations and goals	Voice	Leadership
	(9)	(10)	(11)	(12)	(13)
**Hedges' g**	**0.087*** **	**0.105** **	*Insufficient*	**0.172***	*Insufficient*
**(SE)**	(0.025)	(0.029)	*power*	(0.057)	*power*
**95% CI**	[0.036, 0.137]	[0.046, 0.165]		[0.048, 0.297]	
I^2 (consistency)	93.89%	92.13%		94.49%	
τ^2 (heterogeneity)	0.042	0.032		0.062	
**N of studies**	39	34	4	12	0
**N of effect sizes**	238	152	19	49	0

Table S6. Pooled Effect Sizes Across All Social Safety Net Interventions by Different Outcomes. Average pooled effect sizes are calculated using standardized impacts and reported with standard errors (SEs) and 95% confidence intervals (CIs); All statistical tests reported are two-sided; *p<0.05, **p<0.01, ***p<0.001.

	UCT	CCT	Asset transfers	In-kind transfers	Public work programs	Social care services
	(1)	(2)	(3)	(4)	(5)	(6)
**Hedges' g**	**0.125*** **	**0.046***	**0.109*** **	**0.072**	**0.138***	**0.081*** **
**(SE)**	(0.014)	(0.022)	(0.021)	(0.053)	(0.042)	(0.013)
**95% CI**	[0.097, 0.154]	[-0.001, 0.094]	[0.064, 0.154]	[-0.076, 0.221]	[0.032, 0.243]	[0.052, 0.110]
$\mathbf{I^2}$ (consistency)	89.68%	86.51%	89.98%	77.99%	92.23%	75.08%
τ^2 (heterogeneity)	0.025	0.012	0.035	0.018	0.052	0.007
**N of studies**	54	18	14	5	7	9
**N of effect sizes**	806	162	211	103	102	86
**N of effect sizes removed**	11	5	5	9	4	5
**Cook's d threshold used**	0.01	0.01	0.02	0.005	0.10	0.05

Table S7. Pooled Effect Sizes Across All Outcomes by Different Social Safety Net Interventions, Excluding Influential Effects. Average pooled effect sizes are calculated using standardized impacts from robust variance estimation and reported with standard errors (SEs) and 95% confidence intervals (CIs), removing influential outliers (Fig. S3); All statistical tests reported are two-sided; CCT = conditional cash transfer; UCT = unconditional cash transfer. *p<0.05, **p<0.01, ***p<0.001.

Level	Indicator(s)	Description
Study	Region	Five different regions following the World Bank classification (sub-Saharan Africa, South Asia, Latin America and the Caribbean, Middle East and North Africa, Europe and Central Asia): https://datahelpdesk.worldbank.org/knowledgebase/articles/906519-world-bank-country-and-lending-groups
Study	Income group	Three different income groups following the World Bank classification (low-income, lower-middle income, upper-middle income)—in rare cases, studies were included that took place in a high income country if they qualified as upper-middle income at the time of the implementation: https://datahelpdesk.worldbank.org/knowledgebase/articles/906519-world-bank-country-and-lending-groups
Study	Fragility	Classified as: 1) post-conflict, war or disaster, 2) active conflict or humanitarian crisis or 3) COVID-19 or other pandemic / epidemic if mentioned explicitly in the publication. Otherwise, classified as stable or development setting.
Study	Urban setting	Classified as urban setting if part or all of the evaluation sample lived in an urban or peri-urban area. Urban classification was missing for 63 effects (5% of the sample) – meta-regression results are robust to analysis of only non-missing sample.
Intervention	Type of SSN	See inclusion and exclusion criteria for details (Table S1)
Intervention	Implementer	Three different implementors: 1) Government, 2) Researchers, 3) NGOs, United Nations organizations or others (private sector). When more than one implementer was mentioned, the one primarily responsible for delivering the economic component (rather than plus components) was recorded.
Intervention	Scale	Categorized into three levels: 1) pilot or small scale (if self-described as a pilot or local implementation, often as part of a start-up phase of implementation), 2) mid-level (if described as covering several geographic areas or dozens of clusters, but not yet at large-scale), 3) at scale (an established program, covering many geographic areas or majority of a country or reaching hundreds of thousands of participants).
Intervention	Value of economic benefit	Reported monthly value (in USD) for economic benefit(s), including in purchasing power parity (PPP) if given, during the intervention period. Monthly values are calculated using weekly, bi-monthly or other duration equivalents. Meta-regressions are robust to controlling for an indicator of PPP (versus nominal). Averages for values are taken if there is variation in economic benefits within an intervention arm. Currency conversions use the midpoint of the intervention period using Oanda currency converter. Value of the transfer was missing for 54 effects (4% of the sample) – meta-regression results are robust to analysis of only non-missing sample.
Intervention	Poverty targeted	Indicator = 1 if the intervention targeting was described to include poverty as a factor, either via a proxy means test, categorical or geographical targeting. Included mention of ‘poor’, ‘ultra-poor’, ‘labor-constrained’ etc.

Intervention	Gender targeted	Indicator = 1 if the intervention targeting was described to include gender as a factor, for example, mention of targeting mothers, female caregivers, adolescent girls, women entrepreneurs, primary adult female or female-headed household.
Intervention	Plus component	Indicators for if the intervention arm has at least one plus component, defined as any complementary layered or integrated additional programming, including the following: i) training and information, ii) livelihood or economic, iii) health, including mental health or psychological, social behavior change for health outcomes, child protection or violence component or iv) other. Interventions were classified first as (i) training and information regardless of if they incorporated training on a health or economic component and thereafter into additional categories. Interventions were coded as gender sensitive if they specifically targeted women or were designed around gender considerations, and gender neutral if otherwise.
Intervention	Female sample age	Mean age in years of the female sample for which impacts are estimated and splines are generated for the following ranges: 1) 24 years or younger, 2) 25-39 years, 3) 40 years or older. If no mean age is given for the full sample, control sample is recorded or age range (from which a mean figure is calculated). Mean age of the females' spouse (husband) is substituted if no female age is given. Age is recorded up to one decimal point. After replacements, age of the woman was missing for 298 effects (23% of the sample) – meta-regression results are robust to analysis of only non-missing sample.
Effect	Outcome category	See inclusion and exclusion criteria for details (Table S1)
Effect	Duration of intervention	Coded in months (rounding up to the whole month, thus a lump sum transfer with no other component was coded as one month) and indicator = 1 if the value was 12 months or higher. For intervention with multiple components, the intervention period representing the longest total duration was recorded.
Effect	Time post intervention at survey	Coded in months (rounding down to zero if the follow-up survey was completed while the intervention was ongoing) and indicator = 1 if the value was 12 months or higher. For surveys covering two or more months, averages were taken and the mean post-intervention time was recorded.

Table S8. Indicator Definitions for Covariates at the Publication, Intervention and Effect Levels.

Authors	Year	Country	Type(s) of SSN	Cost estimate	Gender-specific estimates?	Interpretation
Gazeaud et al.	2022	Tunisia	UCT	BCR: 16.9	No	Considering household consumption and assets at two years, the BCR is 16.9 (using a social discount rate of 5%); the benefits fully exceed the costs after 1.2 years of intervention.
Rahman et al.	2021	Bangladesh	UCT	BCR: 8.47	No	Considering household consumption (years 1 and 2) and assets (year 2), the BCR is 8.47 using a social discount rate of 5% and assuming benefits last for 20 years from the transfer date.
Hojman & Boo	2022	Nicaragua	Social care services	BCR: 6.2	Partially	Considering mother's income and child noncognitive skill gains (on future earnings), the BCR is 6.2 (using a 3% interest rate); the marginal value of public funds is 70, putting it in the top 20% of those recently reviewed.
Banerjee et al.	2015	India	Asset transfer; UCT	BCR: 4.3; IRR: 23%	No	Considering household consumption (each year) and assets (at three years), the BCR is 4.33 and an IRR of 23.4% (using a social discount rate of 5%).
Angelucci et al.	2022	DRC	UCT	BCR: 3.7; IRR: 20%; NPV: $1,306	No	Considering household non-durable consumption at 12-months and post intervention follow-up the BCR is 3.68, the IRR is 19.9% and the NPV is $1,306 (using a social discount rate of 5%).
Bandiera et al.	2017	Bangladesh	Asset transfer; UCT	BCR: 3.2; IRR: 16-23%	Partially	Assuming household consumption and asset benefits at year four are repeated over 20 years, the program has a BCR of 3.2 (using a social discount rate of 5%); the IRR is between 16% and 23%, depending on assumptions around opportunity cost of time.
Banerjee et al.	2015	Ethiopia	Asset transfer	BCR: 2.6; IRR: 13%	No	Considering household consumption (each year) and assets (at three years), the BCR is 2.6 and an IRR of 13.3% (using a social discount rate of 5%).
Bedoya et al.	2019	Afghanistan	Asset transfer; UCT; Fee waiver or subsidy	BCR: 2.3; IRR: 26%	No	Considering impacts on household non-durable consumption and assuming a continuation over a 10-year period, the BCR is 2.3, the IRR is 26% and the break-even point is 4 years after program start (using a social discount rate of 5%).
Janzen et al.	2023	Nepal	Asset transfer; UCT	BCR: 1.8; NPV: $108	No	Considering the value of goat herds at year four and goat sales over time, the BCR is 1.83 and the NPV of benefits is $108 (using a social discount rate of 10%).

Table S9. Summary of Cost-Benefit Analysis in Included Studies. BCR = benefit cost ratio; CCT = conditional cash transfer; CE = cost-effectiveness; IRR = internal rate of return; NPV = net present value; UCT = unconditional cash transfer.

Authors	Year	Country	Type(s) of SSN	Cost estimate	Gender-specific estimates?	Interpretation
Banerjee et al.	2015	Pakistan	Asset transfer; UCT	BCR: 1.8; IRR: 10%	No	Considering household consumption (each year) and assets (at three years), the BCR is 1.79 and an IRR of 9.5% (using a social discount rate of 5%).
Banerjee et al.	2015	Peru	Asset transfer; UCT	BCR: 1.5; IRR: 8%	No	Considering household consumption (each year) and assets (at three years), the BCR is 1.46 and an IRR of 7.5% (using a social discount rate of 5%).
Banerjee et al.	2015	Ghana	Asset transfer; UCT	BCR: 1.3; IRR: 7%	No	Considering household consumption (each year) and assets (at three years), the BCR is 1.33 and an IRR of 6.90% (using a social discount rate of 5%).
Bedoya et al.	2023	Afghanistan	Asset transfer; UCT; Fee waiver or subsidy	BCR: 1.1	No	Considering household non-durable consumption with post-intervention impacts up to five years (impacts are expected to dissipate by year 9), the BCR is 1.1 with the break-even point at 6 years after the asset transfer (using a social discount rate of 5%).
Bossuroy el al.	2022	Niger	UCT	BCR: 0.8-18.0; IRR: -9-73%; CE: various	No	Considering household consumption, if all impacts dissipate after two years, the BCR is 0.80 (capital) and 1.27 (full package) with IRRs of -9% (capital) and 21% (full package); If all impacts are sustained into perpetuity, the BCR is10.38 (capital) and 18.04 (full package) with IRRs of 48% (capital) and 73% (full package) (using a social discount rate of 5%); Full package is 1.6 - 1.7x higher BCR as compared to capital only arm.
Crépon et al.	2023	Egypt	UCT; Food, voucher or in-kind	BCR: 1.0-2.0; CE: various	Yes	Considering impacts on women's labor income are sustained for 30 (40) months, the BCR for in-kind grants is 1.58 (1.99) and for cash grants is 0.97 (1.22); Based on women's labor income, in-kind grants would cover the cost of the intervention after 17.8 months, while cash grants would cover the cost of the intervention after 31.28 months; On cost-effectiveness, for women's employment: Grants (both cash and in-kind) create jobs for women at a cost of 5.9x cost of the grant, while loans create the same of 2.42x cost of the grant; For subjective wellbeing: Loans increase 1 "util" for a cost of 1.03x cost of the grant, while in-kind would cost 2.76 for the same increase (no significant impacts for cash grants).

Table S9. Summary of Cost-Benefit Analysis in Included Studies (continued, 2). BCR = benefit cost ratio; CCT = conditional cash transfer; CE = cost-effectiveness; IRR = internal rate of return; NPV = net present value; UCT = unconditional cash transfer.

Authors	Year	Country	Type(s) of SSN	Cost estimate	Gender-specific estimates?	Interpretation
Orkin et al.	2023	Kenya	UCT	BCR: 0.5-1.0; CE: various	No	Considering household non-durable consumption, education, housing and land expenditures, and non-land asset stocks at endline (17-months after the intervention, thereafter dissipating), the BCR for the psychological only intervention is 0.96, the cash is 0.56 and the combined is 0.46, making the psychological only of higher cost effectiveness (2-6x) as compared to the cash only arm.
Botea et al.	2023	Zambia	UCT	BCR: 0.4-8.5; IRR: -64-42%	No	Considering household consumption at one-year post-intervention, the BCR ranges from 0.36-7.27 and IRR ranges from -64%-36% for the full package (considering complete dissipation vs. perpetuity, BCR is >1 assumption 25% dissipation); BCR ranges from 0.42-8.42 and IRR ranges from -58%-42% for the financial capital arm (considering the same scenarios).
Banerjee et al.	2022	Ghana	Asset transfer; UCT	BCR: 0-1.2	No	Considering household consumption (each year) and assets (at three years), the BCR of the full package is 1.2, while the asset transfer only arm has a BCR of effectively zero (using a social discount rate of 5%).
Banerjee et al.	2015	Honduras	Asset transfer; Food, voucher or in-kind transfer	BCR: -1.98	No	Considering household consumption (each year) and assets (at three years), the BCR is -1.98 (using a social discount rate of 5%).
Blattman et al.	2016	Uganda	CCT	IRR: 23-24%; NPV: $9,309-$10,302	No	Considering household non-durable consumption, BCRs are 23-24% and NPV is $9,309-$10,302 (with and without group training), indicating the present value of consumption is nearly 5x the cost of the program (using a social discount rate of 5%).
Attanasio et al.	2011	Colombia	CCT	IRR: 22-35%	Yes	Considering employment and earnings impacts, gains are $3,805 (if permanent) and $1,478 (if depreciation occurs 10% annually), leading to IRR of 35% and 21.6%, respectively.

Table S9. Summary of Cost-Benefit Analysis in Included Studies (continued, 3). BCR = benefit cost ratio; CCT = conditional cash transfer; CE = cost-effectiveness; IRR = internal rate of return; NPV = net present value; UCT = unconditional cash transfer.

Authors	Year	Country	Type(s) of SSN	Cost estimate	Gender-specific estimates?	Interpretation
Rosas & Sabarwal	2016	Sierra Leone	Public works	CE: various	Partially	Cost per temporary job created (for men and women) is $198, while the cost per additional $1 income generated from these jobs is $7.
Brudevold-Newman et al.	2017	Kenya	UCT; Asset transfer	CE: various	Yes	At 7-10 months and 14-22 months post-treatment, the cash grant impacts on income are generally larger than micro franchising (but only statistically significantly different at the 10% level in the 7–10-month follow-up) and less expensive ($286 vs. $376-494); Cash grants are more cost-effective as compared to micro franchising in increasing income.
Handa et al.	2018	Zambia	UCT	Multiplier: 1.6	Partially	Pooled estimates across 24- and 36-month follow-ups indicate a multiplier of 1.61 (households spend or save 61% more than what they receive).
Handa et al.	2018	Zambia	UCT	Multiplier: 1.7	Partially	Estimates pooled across 24- and 36-month follow-ups indicate a multiplier of 1.72 (households spend or save 72% more than what they receive).

Table S9. Summary of Cost-Benefit Analysis in Included Studies (continued, 4). BCR = benefit cost ratio; CCT = conditional cash transfer; CE = cost-effectiveness; IRR = internal rate of return; NPV = net present value; UCT = unconditional cash transfer.

Study	Setting	Intervention(s)	Outcome(s)	Studies	Effects	Pooled effect size
Brody et al. (2015)[a]	LMICs	Economic self-help groups	Empowerment	7	NR	0.18** [0.5, 0.31]
Duvendack et al. (2023)	LMICs	Microcredit and savings	Economic empowerment	17	173	0.027** [0.003, 0.051]
			Agency	12	49	0.019* [-0.003, 0.040]
Lwamba et al. (2022)	Fragile contexts	Asset transfers	Access to and ownership of assets, credit and incomes	11	NR	0.34*** [0.22, 0.46]
			Decent work	5	NR	0.10 [-0.00, 0.20]
			Decision-making	5	NR	0.07* [0.04, 0.11]
		Cash transfers	Access to and ownership of assets, credit and incomes	12	NR	0.22* [0.12, 0.31]
			Decent work	6	NR	0.18 [-0.01, 0.36]
			Decision-making	6	NR	0.03 [-0.31, 0.09]
		Self-help groups	Access to and ownership of assets, credit and incomes	7	NR	0.31** [0.03, 0.60]
			Freedom of movement and association	7	NR	0.18 [0.05, 0.31]
			Decision-making	6	NR	0.04 [-0.04. 0.12]
			Community participation	5	NR	0.07 [-0.01, 0.16]
Stöterau et al. (2022)	LMICs	Vocational training	Employment and earnings	50	465	0.109** [0.042, 0.177]
Vaessen et al. (2014)[b]	LMICs	Microcredit	Control over household spending	14	NR	0.069 [0.003, 0.141]

Table S10. Summary of related meta-analyses on women's economic achievement and agency outcomes (2013 – 2023). Pooled effects are included for the figure which represents the greatest number of studies within each review; pooled effects are only if studies meta-analyze five or more studies; *p<0.05, **p<0.01, ***p<0.001; LMICs = low- and middle-income countries, NR = not reported. [a] pooled effects are reported from experimental and quasi-experimental medium risk of bias, as these represent the effects for the largest pool of studies.[b] pooled effects are for quasi-experimental studies, as experimental studies are pooled from only four studies (effects are not significant); pooled effects for quasi-experimental studies without removing outliers (3 studies) results in positive effects: 0.129** [0.035, 0.222].

Section and Topic	Item #	Checklist item	Location where item is reported
**TITLE**			
Title	1	Identify the report as a systematic review.	Page 1
**ABSTRACT**			
Abstract	2	See the PRISMA 2020 for Abstracts checklist.	Table S12
**INTRODUCTION**			
Rationale	3	Describe the rationale for the review in the context of existing knowledge.	Pages 2-3, 5-6
Objectives	4	Provide an explicit statement of the objective(s) or question(s) the review addresses.	Page 4
**METHODS**			
Eligibility criteria	5	Specify the inclusion and exclusion criteria for the review and how studies were grouped for the syntheses.	Pages 18 – 20, Table S1
Information sources	6	Specify all databases, registers, websites, organisations, reference lists and other sources searched or consulted to identify studies. Specify the date when each source was last searched or consulted.	Pages 20 – 21, Figure 1
Search strategy	7	Present the full search strategies for all databases, registers and websites, including any filters and limits used.	Table S14
Selection process	8	Specify the methods used to decide whether a study met the inclusion criteria of the review, including how many reviewers screened each record and each report retrieved, whether they worked independently, and if applicable, details of automation tools used in the process.	Pages 21 – 24
Data collection process	9	Specify the methods used to collect data from reports, including how many reviewers collected data from each report, whether they worked independently, any processes for obtaining or confirming data from study investigators, and if applicable, details of automation tools used in the process.	Page 24 – 25
Data items	10a	List and define all outcomes for which data were sought. Specify whether all results that were compatible with each outcome domain in each study were sought (e.g. for all measures, time points, analyses), and if not, the methods used to decide which results to collect.	Pages 19 – 20, Table S1
	10b	List and define all other variables for which data were sought (e.g. participant and intervention characteristics, funding sources). Describe any assumptions made about any missing or unclear information.	Page 29 – 31, Table S8
Study risk of bias assessment	11	Specify the methods used to assess risk of bias in the included studies, including details of the tool(s) used, how many reviewers assessed each study and whether they worked independently, and if applicable, details of automation tools used in the process.	Page 25 – 26, Table S15
Effect measures	12	Specify for each outcome the effect measure(s) (e.g. risk ratio, mean difference) used in the synthesis or presentation of results.	Pages 26 – 28

Synthesis methods	13a	Describe the processes used to decide which studies were eligible for each synthesis (e.g. tabulating the study intervention characteristics and comparing against the planned groups for each synthesis (item #5)).	Pages 6 – 8, S12
	13b	Describe any methods required to prepare the data for presentation or synthesis, such as handling of missing summary statistics, or data conversions.	Pages S10 – 11, S15, Table S4
	13c	Describe any methods used to tabulate or visually display results of individual studies and syntheses.	Page 7 – 9
	13d	Describe any methods used to synthesize results and provide a rationale for the choice(s). If meta-analysis was performed, describe the model(s), method(s) to identify the presence and extent of statistical heterogeneity, and software package(s) used.	Pages 28 – 31
	13e	Describe any methods used to explore possible causes of heterogeneity among study results (e.g. subgroup analysis, meta-regression).	Pages 28 – 31
	13f	Describe any sensitivity analyses conducted to assess robustness of the synthesized results.	Page 31 – 34
Reporting bias assessment	14	Describe any methods used to assess risk of bias due to missing results in a synthesis (arising from reporting biases).	Page 34, Fig. S6
Certainty assessment	15	Describe any methods used to assess certainty (or confidence) in the body of evidence for an outcome.	Pages 32, Table S7
**RESULTS**			
Study selection	16a	Describe the results of the search and selection process, from the number of records identified in the search to the number of studies included in the review, ideally using a flow diagram.	Pages 6 – 7, 22 – 23, Fig. 1
	16b	Cite studies that might appear to meet the inclusion criteria, but which were excluded, and explain why they were excluded.	Page 23
Study characteristics	17	Cite each included study and present its characteristics.	Table S2, Pages S42 – S56
Risk of bias in studies	18	Present assessments of risk of bias for each included study.	Table S2
Results of individual studies	19	For all outcomes, present, for each study: (a) summary statistics for each group (where appropriate) and (b) an effect estimate and its precision (e.g. confidence/credible interval), ideally using structured tables or plots.	Not included, as explained on page 29: “ Due to the large number of individual effect sizes, we do not report summary statistics for each individual effect, including means for intervention and control groups, impact estimates and measures of precision (e.g., confidence intervals or standard errors).”

Results of syntheses	20a	For each synthesis, briefly summarise the characteristics and risk of bias among contributing studies.	Table 1
	20b	Present results of all statistical syntheses conducted. If meta-analysis was done, present for each the summary estimate and its precision (e.g. confidence/credible interval) and measures of statistical heterogeneity. If comparing groups, describe the direction of the effect.	Fig. 3, Tables 2, S3 – S6
	20c	Present results of all investigations of possible causes of heterogeneity among study results.	Pages 11 – 12, Table 2
	20d	Present results of all sensitivity analyses conducted to assess the robustness of the synthesized results.	Pages 32 – 34, Table S7
Reporting biases	21	Present assessments of risk of bias due to missing results (arising from reporting biases) for each synthesis assessed.	Not included as tests identified no significant risk of bias due to missing results: Page 34, Fig. S6
Certainty of evidence	22	Present assessments of certainty (or confidence) in the body of evidence for each outcome assessed.	Tables S3 – S6
**DISCUSSION**			
Discussion	23a	Provide a general interpretation of the results in the context of other evidence.	Pages 13 – 15
	23b	Discuss any limitations of the evidence included in the review.	Pages 16
	23c	Discuss any limitations of the review processes used.	Page 16
	23d	Discuss implications of the results for practice, policy, and future research.	Pages 13 – 17
**OTHER INFORMATION**			
Registration and protocol	24a	Provide registration information for the review, including register name and registration number, or state that the review was not registered.	Page 18
	24b	Indicate where the review protocol can be accessed, or state that a protocol was not prepared.	Page 18
	24c	Describe and explain any amendments to information provided at registration or in the protocol.	Page 18
Support	25	Describe sources of financial or non-financial support for the review, and the role of the funders or sponsors in the review.	Page 35, acknowledgements
Competing interests	26	Declare any competing interests of review authors.	Page 36, competing interest statement
Availability of data, code and other materials	27	Report which of the following are publicly available and where they can be found: template data collection forms; data extracted from included studies; data used for all analyses; analytic code; any other materials used in the review.	Page 34, data and code availability statement

Table S11. Preferred Reporting Items for Systematic Reviews and Meta-Analyses (PRISMA) Checklist.

Section and Topic	Item #	Checklist item	Reported (Yes/No)
**TITLE**			
Title	1	Identify the report as a systematic review.	Yes
**BACKGROUND**			
Objectives	2	Provide an explicit statement of the main objective(s) or question(s) the review addresses.	Yes
**METHODS**			
Eligibility criteria	3	Specify the inclusion and exclusion criteria for the review.	Yes
Information sources	4	Specify the information sources (e.g. databases, registers) used to identify studies and the date when each was last searched.	Yes
Risk of bias	5	Specify the methods used to assess risk of bias in the included studies.	Yes
Synthesis of results	6	Specify the methods used to present and synthesize results.	Yes
**RESULTS**			
Included studies	7	Give the total number of included studies and participants and summarise relevant characteristics of studies.	Yes
Synthesis of results	8	Present results for main outcomes, preferably indicating the number of included studies and participants for each. If meta-analysis was done, report the summary estimate and confidence/credible interval. If comparing groups, indicate the direction of the effect (i.e. which group is favored).	Yes
**DISCUSSION**			
Limitations of evidence	9	Provide a brief summary of the limitations of the evidence included in the review (e.g. study risk of bias, inconsistency and imprecision).	Yes
Interpretation	10	Provide a general interpretation of the results and important implications.	Yes
**OTHER**			
Funding	11	Specify the primary source of funding for the review.	Provided in acknowledgements
Registration	12	Provide the register name and registration number.	Provided in main text

Table S12. Preferred Reporting Items for Systematic Reviews and Meta-Analyses (PRISMA) Checklist for Abstracts.

No	Type	Authors	Title	Hits across databases		
				Scopus	PubMed	Web of Science
Total hits (English only, journal articles and working papers)				1,815	180	1,387
1	Childcare voucher	Clark et al. 2019	The impact of childcare on poor urban women's economic empowerment in Africa	Yes	Yes	Yes
2	Cash transfer	Baird et al. 2019	When the money runs out: Do cash transfers have sustained effects on human capital accumulation?	No	Yes*	No
3	Cash transfer	Kilburn et al. 2019	Cash Transfers, Young Women's Economic Well-Being, and HIV Risk: Evidence from HPTN 068	Yes	Yes*	Yes*
4	Business grant	Blattman et al. 2020	The long-term impacts of grants on poverty: Nine-Year evidence from Uganda's Youth Opportunities Program	Yes*	No	No
5	Public work program	Perova et al. 2021	Public works programs and Gender-based violence: Evidence from Lao PDR	Yes*	No	No
6	Food transfer	Peterman et al. 2021	Measuring women's decisionmaking: Indicator choice and survey experiments from cash and food transfer evaluation in Ecuador, Uganda and Yemen	Yes*	No	No
7	Graduation model	Banerjee et al. 2015	A multifaceted program causes lasting progress for the very poor: Evidence from six countries	Yes*	No	No

Table S13. Search String Primary Papers and Pilot Results. Primary papers are well known examples published by 2022, considering a variety of SSN types and outcomes; Figures for total hits by database differ from Fig. 1 as the total in this table excludes ongoing hits since the start of the search process. * = indicates an indirect hit via citation in an existing identified paper.

English	Spanish	French
((TITLE-ABS-KEY (“social safety net*” OR “social assistance” OR “social security” OR “cash transfer*” OR “family allowance*” OR welfare OR (allowance W/3 financial) OR (allowance W/3 economic) OR (assistance W/3 financial) OR (assistance W/3 economic) OR “social security” OR “child grant*” OR “cash grant*” OR “child benefit*” OR “maternity benefit*” OR “paternity benefit*” OR “child subsid*” OR “cash support” OR “pension*” OR “death benefit*” OR “survivor* benefit*” OR “burial fund” OR “disability benefit*” OR “UCT” OR “CCT” OR “basic income” OR “income guarantee” OR “UBI” OR “unemployment benefit*” OR “cash plus” OR “food transfer*” OR “voucher*” OR “In-kind transfer*” OR “food stamp*” OR “food ration*” OR “food parcel” OR “school feeding” OR “school supplies” OR “therapeutic feeding” OR “food support” OR “asset transfer*” OR “livestock transfer*” OR “graduation program*” OR “graduation model” OR “business start-up grant*” OR “business grant*” OR “public work*” OR “cash-for-work” OR “cash for work” OR “food-for-work” OR “food for work” OR “fee waiver*” OR “subsid*” OR “tax credit*” OR “tax break*” OR “financial incentive*” OR incentive* OR “insurance exception” OR “insurance waiver*” OR “education waiver*” OR “housing subsid*” OR “housing allowance*” OR “rental subsid*” OR “input subsidy” OR “transportation benefit*” OR “utility subsid*” OR “child care” OR “day care” OR “home-based care” OR “family support services” OR “child protection” OR “disaster relief” OR “humanitarian aid” OR “emergency relief”)) AND	((TITLE-ABS-KEY (“red de seguridad social*” OR “asistencia social” OR “seguridad social” OR “transferencia de efectivo*” OR “subsidio familiar*” OR bienestar OR (subsidio W/3 financiero) OR (subsidio W/3 económico) OR (ayuda W/3 financiera) OR (ayuda W/3 económica) OR “seguridad social” OR “subsidio para niños*” OR “subsidio en efectivo*” OR “subsidio familiar*” OR “beneficio de maternidad*” OR “beneficio de paternidad*” OR “subsidio infantil*” OR “ayuda en efectivo” OR “pensión*” OR “beneficio por fallecimiento*” OR “beneficio* de supervivencia*” OR “fondo para gastos de entierro” OR “beneficio por incapacidad*” OR “transferencia de efectivo sin condiciones” OR “transferencia de efectivo condicionada” OR “renta básica” OR “garantía de ingresos” OR “renta básica universal” OR “subsidio de desempleo*” OR “efectivo plus” OR “transferencia de alimentos*” OR “voucher*” OR “transferencia en especie*” OR “cupón de alimentos*” OR “ración de alimentos*” OR “paquete de alimentos” OR “alimentación escolar” OR “útiles escolares” OR “alimentación terapéutica” OR “ayuda alimentaria” OR “transferencia de activos*” OR “transferencia de ganado*” OR “programa de graduación*” OR “modelo de graduación” OR “subvención para la creación de empresas*” OR “subvención empresarial*” OR “trabajo público*” OR “efectivo-por-trabajo” OR “efectivo por trabajo” OR “alimentos-por-trabajo” OR “alimentos por trabajo” OR “exención de tarifas*” OR “subsidio*” OR “crédito fiscal*” OR “exención fiscal*” OR “incentivo financiero*” OR incentivo* OR “excepción del seguro” OR “exención del seguro*” OR “exención educativa*” OR “subsid* de vivienda” OR “subsidio de alojamiento*” OR “subsid* de alquiler” OR “subvención de insumos” OR “beneficio* de transporte” OR “subsid* de	((TITLE-ABS-KEY (“filet* de sécurité sociale” OR “assistance sociale” OR “sécurité sociale” OR “transfert* d'argent” OR “allocation* familiale*” OR bien-être OR (allocation W/3 financière) OR (allocation W/3 économique) OR (assistance W/3 financière) OR (assistance W/3 économique) OR “sécurité sociale” OR “subvention* pour enfant*” OR “subvention* financière*” OR “prestation* pour enfant*” OR “prestation* de maternité” OR “prestation* de paternité” OR “subvention* pour enfant*” OR “soutien financier” OR “pension*” OR “prestation* de décès” OR “prestation* de survivant*” OR “fonds funéraires” OR “prestation* d'invalidité” OR “transfert monétaire inconditionnel” OR TMI OR “transfert monétaire conditionnel” OR TMC OR “revenu de base” OR “revenu* garanti*” OR “revenu de base universel” OR RBU OR “prestation* de chômage” OR “prestation* d’assurance-emploi” OR “troc plus” OR “transfert* de nourriture” OR “bon* d’achat*” OR “transfert* en nature” OR “coupon* alimentaire*” OR “ration* alimentaire*” OR “colis de nourriture” OR “alimentation scolaire” OR “fournitures scolaires” OR “alimentation thérapeutique” OR “soutien alimentaire” OR “transfert* d'actif*” OR “transfert* de bétail” OR “programme* de graduation” OR “modèle de graduation” OR “subvention* au démarrage d'entreprise*” OR “subvention* aux entreprises*” OR “trava* publi*” OR “travail rémunéré” OR “travail contre nourriture” OR “dispense* de frais” OR “subvention*” OR “crédit* d'impôt*” OR “allégement* fisca*” OR “incitatif* financier*” OR incitatif * OR “exclusion* d’assurance*” OR “renonciation* à l’assurance*” OR “renonciation* aux assurances” OR “dérogation* à l’éducation” OR “subvention* au* logement*” OR “allocation* pour logement*” OR “subvention* de location” OR “subvention aux intrants” OR “prestation* de

((TITLE-ABS-KEY ("woman*" OR "women*" OR "girl*" OR "gender" OR "female" OR "mother*" OR "grandmother*" OR "sister" OR "sex")) AND<br><br>((TITLE-ABS-KEY ("empowerment" OR "economic standing" OR "economic security" OR "financial wellbeing" OR "economic wellbeing" OR "financial welfare" OR "financial wellbeing" OR "financial security" OR "financial health" OR "financial resilience" OR "economic achievement" OR "labo*r force" OR "labo*r market" OR "employ*" OR "work" or "self-employ*" OR "business" OR "time use" OR "wage" OR "earning*" OR "income" OR "investment*" OR "profit*" OR "expenditure*" OR "childcare" OR "care" OR "unpaid labo*r" OR "care burden" OR "care responsibility" OR "domestic tasks" OR "domestic work" OR "domestic chore*" OR "savings" OR "save" OR "debt" OR "credit" OR "loan*" OR "asset*" OR "livestock" OR "land" OR (agriculture W/3 fertilizer) OR (agricultural W/3 productivity) OR (agriculture W/3 seed) OR (agriculture W/3 harvest) OR "housing" OR "house" OR "agency" OR "choice" OR "voice" OR "leadership" OR "decision-making" OR "decision making" OR "bargaining power" OR "self-efficacy" OR "self efficacy" OR "independence" OR "agency" OR "power" OR "aspiration*" OR "goal*" OR "vote" OR "voting" OR "participation in group*" OR "group participation" OR "social capital" OR "civic engagement" OR "political engagement" OR "political participation" OR "rights" OR "discriminat*")) AND<br><br>((TITLE-ABS-KEY ("random* control* trial*" OR "random* trial*" OR "control* random* trial*" OR "RCT" OR "lottery" OR	servicios públicos" OR "cuidado de niños" OR "guardería" OR "atención domiciliaria" OR "servicios de apoyo familiar" OR "protección de la infancia" OR "ayuda en desastres" OR "ayuda humanitaria" OR "ayuda de emergencia")) AND<br><br>((TITLE-ABS-KEY ("mujer*" OR "mujeres*" OR " chica*" OR " género" OR "femenino" OR "madre*" OR "abuela*" OR "hermana" OR "sexo")) AND<br><br>((TITLE-ABS-KEY ("empoderamiento" OR "situación económica" OR "seguridad económica" OR "bienestar financiero" OR "bienestar económico" OR "asistencia financiera" OR "prosperidad financiera" OR "seguridad financiera" OR "salud financiera" OR "resiliencia financiera" OR "logro económico" OR "fuerza labo*ral" OR "mercado labo*ral" OR "emplead*" OR "trabajo" or "autónomo" OR "independiente" OR "empresa" OR "uso del tiempo" OR "salario" OR "ingreso*" OR "sueldo" OR "inversi*" OR "ganancia*" OR "gasto*" OR "cuidado de niños" OR "asistencia" OR "trabajo no remunerado" OR "sobrecarga del cuidado" OR "responsabilidad del cuidado" OR "tareas domésticas" OR "trabajo doméstico" OR "labor*es domésticas" OR "ahorros" OR "ahorrar" OR "deuda" OR "crédito" OR "préstamo*" OR "activo*" OR "ganado" OR "tierra" OR (agricultura W/3 fertilizante) OR (productividad W/3 agrícola) OR (semillas W/3 agricultura) OR (cosecha W/3 agrícola) OR "alojamiento" OR "vivienda" OR "agencia" OR "elección" OR "voz" OR "liderazgo" OR "toma-de-decisiones" OR "toma de decisiones" OR "poder de negociación" OR "autoeficacia" OR "auto eficacia" OR "independencia" OR "organismo" OR "poder" OR "aspiración*" OR "objetivo*" OR "voto" OR "votación" OR "participación en grupo*" OR "participación grupal" OR "capital social" OR "compromiso	transport*" OR "subvention* aux services" OR "services de garde" OR "garderie" OR "soins à domicile" OR "services de soutien aux familles" OR "protection de l'enfance" OR "secours aux sinistrés" OR " aide humanitaire" OR "secours d'urgence")) AND<br><br>((TITLE-ABS-KEY ("femme" OR "femmes" OR "fille*" OR "genre" OR "féminin" OR "mère*" OR " grand*-mère*" OR "sœur" OR "sexe")) AND<br><br>((TITLE-ABS-KEY ("autonomisation" OR "situation économique" OR "sécurité économique" OR "bien-être financier" OR "bien-être économique" OR "prospérité financière" OR "bien-être financier" OR "sécurité financière" OR "santé financière" OR "résilience financière" OR "réussite économique" OR "population* active*" OR "marché* du travail" OR "emplo*" OR "travail" or "travail indépendant" OR "entreprise" OR "temps d'utilisation" OR "salaire" OR "gain*" OR "revenu" OR "investissement*" OR "profit*" OR "dépense*" OR "service* de garde d'enfant*" OR "soin" OR "travail non rémunéré" OR "charge des soins" OR "responsabilité* des soins" OR "tâche* domestique*" OR "travail domestique" OR "tâche domestique*" OR "épargne" OR "économiser" OR "dette" OR "crédit" OR "prêt*" OR "actif*" OR " bétail" OR "terrain" OR (agriculture W/3 engrais) OR (productivité W/3 agricole) OR (agriculture W/3 semence) OR (agriculture W/3 récolte) OR " logement" OR "maison" OR "agence" OR "choix" OR "voix" OR "leadership" OR " prise de décision" OR " prise de décision" OR " pouvoir de négociation" OR " auto-efficacité" OR " auto-efficacité " OR " indépendance" OR "agence*" OR "pouvoir" OR "aspiration*" OR "but*" OR "vote*" OR "voter" OR "participation en groupe*" OR " participation de groupe*" OR " capital social" OR "

“impact evaluation” OR “causal effect” OR “causal impact” or “clinical trial”))<br><br>((TITLE-ABS-KEY (“global south” OR "deprived countries" OR "deprived country" OR "deprived nation" OR "deprived nations" OR "deprived population" OR "deprived populations" OR "deprived world" OR "developing countries" OR "developing country" OR "developing economies" OR "developing economy" OR "developing nation" OR "developing nations" OR "developing population" OR "developing populations" OR "developing world" OR "less developed countries" OR "less developed country" OR "less developed economies" OR "less developed economy" OR "less developed nation" OR "less developed nations" OR "less developed population" OR "less developed populations" OR "less developed world" OR "lesser developed countries" OR "lesser developed country" OR "lesser developed economies" OR "lesser developed economy" OR "lesser developed nation" OR "lesser developed nations" OR "lesser developed population" OR "lesser developed populations" OR "lesser developed world" OR "LMIC" OR "LMICS" OR "low gdp" OR "low gnp" OR "low gross domestic" OR "low gross national" OR "low income countries" OR "low income country" OR "low income economies" OR "low income economy" OR "low income nation" OR "low income nations" OR "low income population" OR "low income populations" OR "lower gdp" OR "lower gnp" OR "lower gross domestic" OR "lower gross national" OR "lower income countries" OR "lower income country" OR "lower income economies" OR "lower income economy" OR "lower income nation" OR "lower income nations" OR "lower income population"	cívico” OR “compromiso político” OR “participación política” OR “derechos” OR “discrimina*”)) AND<br><br>((TITLE-ABS-KEY (“ensayo* de control* aleatorio*” OR “ensayo* aleatorio*” OR “ensayo* controlado* aleatorio*” OR “ECA” OR “sorteo” OR “evaluación de impacto” OR “efecto causal” OR “impacto causal” or “ensayo clínico”))<br><br>((TITLE-ABS-KEY (“sur global” OR " países desfavorecidos" OR "país desfavorecido" OR " nación desfavorecida" OR "naciones desfavorecidas" OR " población desfavorecida" OR "poblaciones desfavorecidas" OR "mundo desfavorecido " OR "países en desarrollo" OR " país en desarrollo" OR "economías en desarrollo" OR "economía en desarrollo" OR "nación en desarrollo" OR "naciones en desarrollo" OR "población en desarrollo" OR "poblaciones en desarrollo" OR "mundo en desarrollo" OR "países menos desarrollados" OR "país menos desarrollado" OR "economías menos desarrolladas" OR "economía menos desarrollada" OR " nación menos desarrollada" OR "naciones menos desarrolladas" OR "población menos desarrollada" OR "poblaciones menos desarrolladas" OR "mundo menos desarrollado" OR "países subdesarrollados" OR "país subdesarrollado" OR "economías subdesarrolladas" OR "economía subdesarrollada" OR "nación subdesarrollada" OR "naciones subdesarrolladas " OR "población subdesarrollada" OR "poblaciones subdesarrolladas" OR "mundo subdesarrollado" OR "países con ingresos bajos y medios" OR "países de renta baja y media" OR " PIB bajo" OR "PNB bajo" OR "bajo producto interno bruto" OR "bajo ingreso nacional bruto" OR "países de bajos ingresos" OR "país de bajos ingresos" OR "economías de bajos ingresos" OR "economía de bajos ingresos" OR "nación de bajos	engagement citoyen” OR “engagement politique” OR “participation politique” OR “droits” OR “discrimin*”)) AND<br><br>((TITLE-ABS-KEY (“aléatoire* contrôle* essai*” OR “aléatoire* essai*” OR “contrôle* aléatoire* essai*” OR “essai* contrôlé* randomisé*" OR "ECR” OR “ loterie” OR “ évaluation d'impact*” OR “effet* de causalité” OR “ impact causal” or “ essai* clinique*”))<br><br>((TITLE-ABS-KEY (“pays du sud” OR " pays défavorisés" OR "pays défavorisé" OR "nation défavorisée" OR "nations défavorisées" OR "population défavorisée" OR "populations défavorisées" OR "pays en développement " OR "pays en développement" OR "économies en développement" OR "économie en développement" OR "nation en développement " OR "nations en développement " OR "population en développement " OR "populations en développement" OR "pays moins développés" OR "pays moins développé" OR "économies moins développées" OR "économie moins développée" OR "nation moins développée" OR "nations moins développées" OR "population moins développée" OR "populations moins développées" OR "pays les moins développés" OR "pays le moins développé" OR "économies les moins développées" OR "économie la moins développée" OR "nation la moins développée" OR "nations les moins développées" OR "population la moins développée" OR "nations les moins développées" OR "pays à faible et moyen revenue” OR “PFMR" OR "pays à faible et moyen revenue” OR PFMRs OR "faible produit intérieur brut” OR "faible produit national brut" OR "faible produit intérieur" OR "faible produit national" OR "pays à faible revenu" OR "pays à faible revenu" OR "économies à faible revenu" OR "économie à faible revenu " OR "nation à faible revenu" OR "nations à

OR "lower income populations" OR "middle income countries" OR "middle income country" OR "middle income economies" OR "middle income economy" OR "middle income nation" OR "middle income nations" OR "middle income population" OR "middle income populations" OR "poor countries" OR "poor country" OR "Poor Economies" OR "Poor Economy" OR "poor nation" OR "poor nations" OR "poor population" OR "poor populations" OR "poor world" OR "poorer countries" OR "poorer country" OR "Poorer Economies" OR "Poorer Economy" OR "poorer nation" OR "poorer nations" OR "poorer population" OR "poorer populations" OR "poorer world" OR "third world" OR "transitional countries" OR "transitional country" OR "Transitional Economies" OR "Transitional Economy" OR "under developed countries" OR "under developed country" OR "under developed economies" OR "under developed economy" OR "under developed nation" OR "under developed nations" OR "under developed population" OR "under developed populations" OR "under developed world" OR "under served countries" OR "under served country" OR "under served nation" OR "under served nations" OR "under served population" OR "under served populations" OR "under served world" OR "underdeveloped countries" OR "underdeveloped country" OR "underdeveloped economies" OR "underdeveloped economy" OR "underdeveloped nation" OR "underdeveloped nations" OR "underdeveloped population" OR "underdeveloped populations" OR "underdeveloped world" OR "underserved countries" OR "underserved country" OR "underserved nation" OR "underserved nations" OR "underserved population" OR "underserved populations" OR "underserved world" OR	ingresos" OR "naciones de bajos ingresos" OR "población de bajos ingresos " OR "poblaciones de bajos ingresos" OR "menor PIB" OR "menor PNB" OR "menor producto interno bruto" OR "menor producto nacional bruto" OR "países de menores ingresos" OR "país de menores ingresos" OR "economías de menores ingresos" OR "economía de menores ingresos" OR "nación de menores ingresos" OR "naciones de menores ingresos" OR "población de menores ingresos" OR "poblaciones de menores ingresos" OR "países de ingresos medios" OR "país de ingresos medios" OR "economías de ingresos medios" OR "economía de ingresos medios" OR "nación de ingresos medios" OR "naciones de ingresos medios" OR "población de ingresos medios" OR "poblaciones de ingresos medios" OR "países pobres" OR "país pobre" OR "economías pobres" OR "economía pobre" OR "nación pobre" OR "naciones pobres" OR "población pobre" OR "poblaciones pobres" OR "mundo pobre" OR "países más pobres" OR "país más pobre" OR "economías más pobres" OR "economía más pobre" OR "nación más pobre" OR "naciones más pobres" OR "población más pobre" OR "poblaciones más pobres" OR "mundo más pobre" OR "tercer mundo" OR "países en transición" OR "país en transición" OR "economías en transición" OR "economía en transición" OR "países en vías de desarrollo" OR "país en vías de desarrollo" OR "economías en vías de desarrollo" OR "economía en vías de desarrollo" OR "nación en vías de desarrollo" OR "naciones en vías de desarrollo" OR "población en vías de desarrollo" OR "poblaciones en vías de desarrollo" OR "mundo en vías de desarrollo" OR "países con servicios insuficientes" OR "país con servicios insuficientes" OR "nación con servicios insuficientes" OR "naciones con servicios insuficientes" OR "población con servicios insuficientes" OR "poblaciones con servicios insuficientes" OR	faible revenu" OR "population à faible revenu" OR "populations à faible revenu" OR "plus faible produit intérieur brut" OR "PIB" OR "plus faible produit national brut" OR "PNB" OR "plus faible produit intérieur" OR "plus faible produit national" OR "pays à plus faible revenu" OR " pays à plus faible revenu" OR " économies à plus faible revenu" OR " économie à plus faible revenu" OR " nation à plus faible revenu" OR " nations à plus faible revenu" OR "population à faible revenu" OR "populations à plus faible revenu " OR "pays à revenu moyen" OR "pays à revenu moyen" OR "économies à revenu moyen" OR "économie à revenu moyen" OR "nation à revenu moyen" OR "nations à revenu moyen" OR "population à revenu moyen" OR " populations à revenu moyen" OR "pays pauvres" OR "pays pauvre" OR "économies pauvres" OR " économie pauvre" OR "nation pauvre" OR "nations pauvres" OR "population pauvre" OR "populations pauvres" OR " pays les plus pauvres" OR "pays le plus pauvre" OR "économies les plus pauvres" OR "économie la plus pauvre" OR "nation la plus pauvre" OR "nations les plus pauvres" OR "population la plus pauvre" OR "populations les plus pauvres" OR "tiers-monde" OR "pays en transition" OR " pays en transition" OR "économies en transition" OR "économie en transition" OR "pays sous-développés" OR "pays sous-développé" OR "économies sous-développées" OR "économie sous-développée" OR "nation sous-développée" OR " nations sous-développées" OR "population sous-développée " OR "populations sous-développées" OR "pays sous-desservis" OR "pays sous-desservi" OR "nation sous-desservie" OR " nations sous-desservies" OR "population sous-desservie" OR "populations sous-desservies" OR "pays sous-développés" OR "pays sous-développé" OR "économies sous-développées" OR "économie sous-développée" OR "nation sous-développée" OR "nations sous-développées" OR

Afghanistan OR Albania OR Algeria OR “American Samoa” OR Angola OR Argentina OR “Argentine Republic” OR Armenia OR Azerbaijan OR Bangladesh OR Belarus OR Byelarus OR Belorussia OR Belize OR Benin OR Bhutan OR Bolivia OR Bosnia OR Botswana OR Brazil OR Bulgaria OR Burma OR “Burkina Faso” OR Burundi OR “Cabo Verde” OR “Cape Verde” OR Cambodia OR Cameroon OR “Central African Republic” OR Chad OR Chile OR China OR Colombia OR Comoros OR Comoros OR Comoro OR Congo OR “Costa Rica” OR “Côte d'Ivoire” OR Cuba OR Djibouti OR Dominica OR “Dominican Republic” OR Ecuador OR Egypt OR “El Salvador” OR Eritrea OR Ethiopia OR Fiji OR Gabon OR Gambia OR Gaza OR “Georgia Republic” OR Georgian OR Ghana OR Grenada OR Grenadines OR Guatemala OR Guinea OR “Guinea Bissau” OR Guyana OR Haiti OR Herzegovina OR Hercegovina OR Honduras OR India OR Indonesia OR Iran OR Iraq OR Jamaica OR Jordan OR Kazakhstan OR Kenya OR Kiribati OR Korea OR Kosovo OR Kyrgyz OR Kirghizia OR Kirghiz OR Kirgizstan OR Kyrgyzstan OR “Lao PDR” OR Laos OR Lebanon OR Lesotho OR Liberia OR Libya OR Macedonia OR Madagascar OR Malawi OR Malay OR Malaya OR Malaysia OR Maldives OR Mali OR “Marshall Islands” OR Mauritania OR Mauritius OR Mexico OR Micronesia OR Moldova OR Mongolia OR Montenegro OR Morocco OR Mozambique OR Myanmar OR Namibia OR Nauru OR Nepal OR Nicaragua OR Niger OR Nigeria OR Pakistan OR Palau OR Panama OR “Papua New Guinea” OR Paraguay OR Peru OR Philippines OR Philippines OR Philippines OR Philippines OR Principe OR Romania OR Rwanda OR Ruanda OR Samoa OR “Sao Tome” OR Senegal OR	"mundo con servicios insuficientes" OR "países en subdesarrollo" OR "país en subdesarrollo" OR "economías en subdesarrollo" OR "economía en subdesarrollo" OR "nación en subdesarrollo" OR "naciones en subdesarrollo" OR "población en subdesarrollo" OR "poblaciones en subdesarrollo" OR "mundo en subdesarrollo" OR "países marginados" OR "país marginado" OR "nación marginada" OR "naciones marginadas" OR " población marginada" OR "poblaciones marginadas" OR "mundo marginado" OR Afganistán OR Albania OR Argelia OR "Samoa Americana" OR Angola OR Argentina OR "República Argentina" OR Armenia OR Azerbaiyán OR Bangladesh OR Bielorrusia OR Bielorrusia OR Belice OR Benín OR Bután OR Bolivia OR Bosnia OR Botsuana OR Brasil OR Bulgaria OR Birmania OR "Burkina Faso" OR Burundi OR "Cabo Verde" OR "Cabo Verde" OR Camboya OR Camerún OR "República Centroafricana" OR Chad OR Chile OR China OR Colombia OR Comoras OR Comores OR Comoro OR Congo OR "Costa Rica". OR "Costa de Marfil" OR Cuba OR Djibouti OR Dominica OR "República Dominicana" OR Ecuador OR Egipto OR "El Salvador" OR Eritrea OR Etiopía OR Fiji OR Gabón OR Gambia OR Gaza OR "República de Georgia" OR georgiano OR Ghana OR Granada OR Granadinas OR Guatemala OR Guinea OR "Guinea Bisáu" OR Guyana OR Haití OR Herzegovina OR Hercegovina OR Honduras OR India OR Indonesia OR Irán OR Iraq OR Jamaica OR Jordán OR Kazajstán OR Kenia OR Kiribati OR Corea OR Kosovo OR Kyrgyz OR Kirghizia OR Kirguistán OR Kirguizistán OR "Lao PDR" OR Laos OR Líbano OR Lesoto OR Liberia OR Libia OR Macedonia OR Madagascar OR Malawi OR Malayo OR Malaya OR Malasia OR Maldivas OR Malí OR "Islas Marshall" OR Mauritania OR Mauricio OR México OR Micronesia OR Moldavia	"population sous-développée" OR "populations sous-développées" OR "pays sous desservis" OR "pays sous desservi" OR "nation sous desservie" OR " nations sous desservies" OR "population sous desservie" OR "populations sous desservies" OR Afghanistan OR Albanie OR Algérie OR “Samoa américaines” OR Angola OR Argentine OR “République d'Argentine” OR Arménie OR Azerbaïdjan OR Bangladesh OR Bélarus OR Biélorussie OR Bélize OR Belize OR Bénin OR Bhoutan OR Bolivie OR Bosnie OR Botswana OR Brésil OR Bulgarie OR Birmanie OR “Burkina Faso” OR Burundi OR “Cap-Vert” OR Cambodge OR Cameroun OR “République centrafricaine” OR Tchad OR Chili OR Chine OR Colombie OR Comores OR Congo OR “Costa Rica” OR “Côte d'Ivoire” OR Cuba OR Djibouti OR Dominique OR “République dominicaine” OR Équateur OR Égypte OR “El Salvador” OR Salvador OR Érythrée OR Éthiopie OR Fidji OR Gabon OR Gambie OR Gaza OR “République de Géorgie” OR Géorgie OR Ghana OR Grenade OR Grenadines OR Guatemala OR Guinée OR “Guinée-Bissau” OR Guyane OR Haïti OR Herzégovine OR Honduras OR Inde OR Indonésie OR Iran OR Irak OR Jamaïque OR Jordanie OR Kazakhstan OR Kenya OR Kiribati OR Corée OR Kosovo OR Kirghize OR Kirghizie OR Kirghizstan OR Kirghizistan OR “République démocratique populaire (RDP) lao” OR Laos OR Liban OR Lesotho OR Liberia OR Libye OR Macédoine OR Madagascar OR Malawi OR Malaisie OR Maldives OR Mali OR “Îles Marshall” OR Mauritanie OR Île Maurice OR Mexique OR Micronésie OR Moldavie OR Mongolie OR Monténégro OR Maroc OR Mozambique OR Myanmar OR Namibie OR Nauru OR Népal OR Nicaragua OR Niger OR Nigéria OR Pakistan OR Palaos OR Panama OR “Papouasie-Nouvelle-Guinée” OR Paraguay OR Pérou OR Philippines OR Principe OR Roumanie OR Rwanda OR Samoa

Serbia OR “Sierra Leone” OR “Solomon Islands” OR Somalia OR “South Africa” OR “South Sudan” OR “Sri Lanka” OR “St Lucia” OR “St Vincent” OR Sudan OR Surinam OR Suriname OR Swaziland OR Syria OR “Syrian Arab Republic” OR Tajikistan OR Tadzhikistan OR Tajikistan OR Tadzhik OR Tanzania OR Thailand OR Timor OR Togo OR Tonga OR Tunisia OR Turkey OR Turkmen OR Turkmenistan OR Tuvalu OR Uganda OR Ukraine OR Uruguay OR Uzbek OR Uzbekistan OR Vanuatu OR Venezuela OR Vietnam OR “West Bank” OR Yemen OR Zambia OR Zimbabwe OR “Sub-Sahara* Africa*” OR “Sub-Saharan Africa*” OR “middle east” OR “north Africa” OR “west Africa” OR “Southern Africa” OR “east Africa” or “Arab region” OR “South Asia” OR “Asia Pacific” OR “Pacific Islands” OR “East Asia” OR “Latin America” OR “South America” OR “Caribbean”))<br><br>AND PUBYEAR > 2003 AND PUBYEAR < 2023	OR Mongolia OR Montenegro OR Marruecos OR Mozambique OR Myanmar OR Namibia OR Nauru OR Nepal OR Nicaragua OR Níger OR Nigeria OR Pakistán OR Palau OR Panamá OR "Papúa Nueva Guinea" OR Paraguay OR Perú OR Filipinas OR "Filipinas" OR "Príncipe" OR Rumanía OR Ruanda OR Ruanda OR Samoa OR "Santo Tomé" OR Senegal OR Serbia OR "Sierra Leona" OR "Islas Salomón" OR Somalia OR "Sudáfrica" OR "Sudán del Sur" OR "Sri Lanka" OR "Santa Lucía" OR "San Vicente" OR Sudán OR Surinam OR Suriname OR Swazilandia OR Esuatini OR Siria OR "República Árabe Siria" OR Tajikistán OR Tadzhikistán OR Tayikistán OR Tadzhik OR Tanzania OR Tailandia OR Timor OR Togo OR Tonga OR Túnez OR Turquía OR Turkmenistán OR Tuvalu OR Uganda OR Ucrania OR Uruguay OR Uzbekistán OR Vanuatu OR Venezuela OR Vietnam OR "Cisjordania" OR Yemen OR Zambia OR Zimbabue OR "África Subsahariana*" OR "África subsahariana*" OR "Oriente Medio" OR "África del Norte" OR "África Occidental" OR "África Austral" OR "África Oriental" OR "Región Árabe" OR "Asia Meridional" OR "Asia Pacífico" OR "Islas del Pacífico" OR "Asia Oriental" OR "América Latina" OR "América del Sur" OR "Caribe"))<br><br>AND PUBYEAR > 2003 AND PUBYEAR < 2023	OR “Sao Tomé” OR Sénégal OR Serbie OR “Sierra Leone” OR “Îles Salomon” OR Somalie OR “Afrique du Sud” OR “Soudan du Sud” OR “Sri Lanka” OR “Sainte-Lucie” OR “Saint-Vincent” OR Soudan OR Suriname OR Swaziland OR Eswatini OR Syrie OR “République arabe syrienne” OR Tadjikistan OR Tadjik OR Tanzanie OR Thaïlande OR Timor OR Togo OR Tonga OR Tunisie OR Turquie OR Turkmène OR Turkménistan OR Tuvalu OR Ouganda OR Ukraine OR Uruguay OR Ouzbek OR Ouzbékistan OR Vanuatu OR Venezuela OR Vietnam OR “Cisjordanie” OR Yémen OR Zambie OR Zimbabwe OR “Afrique sub-sahara*” OR “Afrique subsaharien*” OR “Moyen-Orient” OR “Afrique du Nord” OR “Afrique de l'Ouest” OR “Afrique australe” OR “Afrique de l'Est” or “Région arabe” OR “Asie du Sud” OR “Asie-Pacifique” OR “Îles du Pacifique” OR “Asie de l'Est” OR “Amérique Latine” OR “Amérique du Sud” OR “ Caraïbes”))<br><br>AND PUBYEAR > 2003 AND PUBYEAR < 2023

Table S14. Scopus Search String in English, Spanish and French.

Item	Description	Question	Responses
1	Randomization	Was true randomization used for assignment of participants to treatment groups?	Yes, No, Unclear
2	Baseline balance	Were treatment and control groups similar at baseline?	Yes, No, Unclear, NA
3	Participants blinded at baseline	Were participants blind to treatment assignment at baseline?	Yes, No, Unclear, NA
4	Data collectors blinded at baseline	Were outcome assessors blind to treatment assignment at baseline?	Yes, No, Unclear, NA
5	Groups treated identically	Were treatment (and control) groups treated identically over time—except for the intervention of interest?	Yes, No, Unclear
6	Attrition	Was follow-up complete and if not, were differences between groups in terms of their follow-up adequately described and analyzed?	Yes, No, Unclear, NA
7	ITT analysis	Were participants analyzed in the groups to which they were randomized?	Yes, No, Unclear
8	Outcome measure	Were outcomes measured the same way for treatment groups?	Yes, No, Unclear
9	Appropriate power	Was the study appropriately powered	Yes, No, Unclear
10	Statistical analysis	Was appropriate statistical analysis used?	Yes, No, Unclear

Table S15. Revised Joanna Briggs Institute Quality Assessment for Experimental Studies. ITT = intent-to-treat, NA = not applicable; Original items (2) and (5) were dropped ("if allocation to treatment groups was concealed" and "if those delivering the treatment were blinded to assignment") and existing items (3) and (4) were modified to specify applicability to baseline only, rather than over the course of the study. These modifications were made to accommodate norms and feasibility of concealment in social science experiments in which treatment cannot be fully hidden at later stages of the study. Original item (9) regarding conducting validity and reliability checks of outcome measures was also dropped, as psychometric testing and validation is rarely done for economic and agency outcomes. Finally existing item (9) was singled out as its own item (originally part of item 10), as power to detect effects and sample size calculations were deemed to be an important component within social science experimental studies. All JBI tools are available online at: https://synthesismanual.jbi.global

3. Works cited for included papers (alphabetical order)

1. Abdullahi, A., Ali, M., Kipchumba, E. & Sulaiman, M. Supporting Micro-enterprise in Humanitarian Programming: Impact Evaluation of Business Grants versus Unconditional Cash Transfer. *Journal of African Economies* ejac012 (2022) doi:10.1093/jae/ejac012.
2. Aguila, E. & Smith, J. P. Supplemental income program design: A cluster-randomized controlled trial to examine the health and wellbeing effects on older adults by gender, duration, and payment frequency. *Social Science & Medicine* **259**, 113139 (2020).
3. Ahmed, A. U., Hoddinott, J., Roy, S. & Sraboni, E. Transfers, nutrition programming, and economic well-being: Experimental evidence from Bangladesh. *World Development* **173**, 106414 (2024).
4. Ajayi, K. F., Dao, A. & Koussoubé, E. *The Effects of Childcare on Women and Children: Evidence from a Randomized Evaluation in Burkina Faso*. https://www.cgdev.org/publication/effects-childcare-women-and-children-evidence-randomized-evaluation-burkina-faso (2022) doi:10.1596/1813-9450-10239.
5. Alderman, H. *et al. Impact Evaluation of the Strengthen PSNP4 Institutions and Resilience (SPIR) Development Food Security Activity (DFSA) Endline Report*. (2021).
6. Alzúa, M. L., Cruces, G. & Ripani, L. Welfare programs and labor supply in developing countries: experimental evidence from Latin America. *J Popul Econ* **26**, 1255–1284 (2013).

7. Ambler, K., de Brauw, A. & Godlonton, S. Lump-sum Transfers for Agriculture and Household Decision Making. *Department of Economics Working Papers* (2019).

8. American Institutes for Research (AIR). *Zambia's Multiple Category Targeting Grant: 24-Month Impact Report*. https://www.air.org/resource/report/zambias-multiple-category-targeting-grant-24-month-impact-report (2014).

9. Angeles, G. *et al.* Government of Malawi's unconditional cash transfer improves youth mental health. *Social Science & Medicine* **225**, 108–119 (2019).

10. Angelucci, M., Heath, R. & Noble, E. Multifaceted programs targeting women in fragile settings: Evidence from the Democratic Republic of Congo. *Journal of Development Economics* **164**, 103146 (2023).

11. Armand, A., Carneiro, P., Tagliati, F. & Xia, Y. *Can Subsidized Employment Tackle Long-Term Unemployment? Experimental Evidence from North Macedonia*. https://www.iza.org/publications/dp/13478/can-subsidized-employment-tackle-long-term-unemployment-experimental-evidence-from-north-macedonia (2022).

12. Asfaw, S., Davis, B., Dewbre, J., Handa, S. & Winters, P. Cash Transfer Programme, Productive Activities and Labour Supply: Evidence from a Randomised Experiment in Kenya. *The Journal of Development Studies* **50**, 1172–1196 (2014).

13. Attanasio, O., Kugler, A. & Meghir, C. Subsidizing Vocational Training for Disadvantaged Youth in Colombia: Evidence from a Randomized Trial. *American Economic Journal: Applied Economics* **3**, 188–220 (2011).

14. Attanasio, O. *et al.* Public Childcare, Labor Market Outcomes of Caregivers, and Child Development: Experimental Evidence from Brazil. Working Paper at https://doi.org/10.3386/w30653 (2022).

15. Baird, S., McIntosh, C. & Özler, B. When the money runs out: Do cash transfers have sustained effects on human capital accumulation? *Journal of Development Economics* **140**, 169–185 (2019).

16. Bakhtiar, M. M., Fafchamps, M., Goldstein, M., Leonard, K. L. & Papineni, S. *To Defer or Differ: Experimental Evidence on the Role of Cash Transfers on Nigerian Couples' Decision-Making*. https://hdl.handle.net/10568/152230 (2024).

17. Bandiera, O. *et al.* Labor Markets and Poverty in Village Economies. *The Quarterly Journal of Economics* **132**, 811–870 (2017).

18. Banerjee, A. *et al.* A multifaceted program causes lasting progress for the very poor: Evidence from six countries. *Science* **348**, 1260799 (2015).

19. Banerjee, A., Karlan, D., Osei, R., Trachtman, H. & Udry, C. Unpacking a multi-faceted program to build sustainable income for the very poor. *Journal of Development Economics* **155**, 102781 (2022).

20. Banerjee, A., Hanna, R., Kreindler, G. E. & Olken, B. A. Debunking the Stereotype of the Lazy Welfare Recipient: Evidence from Cash Transfer Programs. *The World Bank Research Observer* **32**, 155–184 (2017).

21. Bedi, T., King, M. & Vaillant, J. Gender Targeting and Household Cooperation: Experimental Evidence from a Multifaceted Anti-poverty Program in Malawi. Preprint at (2023).

22. Bedoya, G. *et al.* The Enduring Impacts of a Big Push during Multiple Crises: Experimental Evidence from Afghanistan. (2023) doi:10.1596/1813-9450-10596.

23. Bedoya, G., Coville, A., Haushofer, J., Isaqzadeh, M. & Shapiro, J. P. No Household Left Behind: Afghanistan Targeting the Ultra Poor Impact Evaluation. (2019) doi:10.3386/w25981.

24. Benedetti, F., Ibarrarán, P. & McEwan, P. J. Do Education and Health Conditions Matter in a Large Cash Transfer? Evidence from a Honduran Experiment. *Economic Development and Cultural Change* **64**, 759–793 (2016).

25. Bjorvatn, K. *et al.* Childcare, Labor Supply, and Business Development: Experimental Evidence from Uganda. SSRN Scholarly Paper at https://papers.ssrn.com/abstract=4121426 (2022).

26. Blattman, C., Fiala, N. & Martinez, S. Generating Skilled Self-Employment in Developing Countries: Experimental Evidence from Uganda*. *Quarterly Journal of Economics* **129**, 697–752 (2014).

27. Blattman, C., Fiala, N. & Martinez, S. The Long-Term Impacts of Grants on Poverty: Nine-Year Evidence from Uganda's Youth Opportunities Program. *American Economic Review: Insights* **2**, 287–304 (2020).

28. Blattman, C., Green, E. P., Jamison, J., Lehmann, M. C. & Annan, J. The Returns to Microenterprise Support among the Ultrapoor: A Field Experiment in Postwar Uganda. *American Economic Journal: Applied Economics* **8**, 35–64 (2016).

29. Bonilla, J. *et al.* Cash for Women's Empowerment? A Mixed-Methods Evaluation of the Government of Zambia's Child Grant Program. *World Development* **95**, 55–72 (2017).

30. Bossuroy, T. *et al.* Tackling psychosocial and capital constraints to alleviate poverty. *Nature* **605**, 291–297 (2022).

31. Botea, I., Brudevold-Newman, A., Goldstein, M., Low, C. & Roberts, G. Supporting Women's Livelihoods at Scale: Evidence from a Nationwide Multi-Faceted Program. SSRN Scholarly Paper at https://papers.ssrn.com/abstract=4560552 (2023).

32. Briaux, J. *et al.* Evaluation of an unconditional cash transfer program targeting children's first-1,000–days linear growth in rural Togo: A cluster-randomized controlled trial. *PLOS Medicine* **17**, e1003388 (2020).

33. Brooks, W., Donovan, K., Johnson, T. R. & Oluoch-Aridi, J. Cash transfers as a response to COVID-19: Experimental evidence from Kenya. *Journal of Development Economics* **158**, 102929 (2022).

34. Brudevold-Newman, A., Honorati, M., Jakiela, P. & Ozier, O. *A Firm of One's Own : Experimental Evidence on Credit Constraints and Occupational Choice*. https://documents.worldbank.org/en/publication/documents-reports/documentdetail/428361487270218330/A-firm-of-ones-own-experimental-evidence-on-credit-constraints-and-occupational-choice (2017).

35. Buller, A. M., Hidrobo, M., Peterman, A. & Heise, L. The way to a man's heart is through his stomach?: a mixed methods study on causal mechanisms through which cash and in-kind food transfers decreased intimate partner violence. *BMC Public Health* **16**, 488 (2016).

36. Calderone, M., Fiala, N., Melyoki, L. L., Schoofs, A. & Steinacher, R. *Making Intense Skills Training Work at Scale: Evidence on Business and Labor Market Outcomes in Tanzania*. https://www.econstor.eu/handle/10419/266696 (2022) doi:10.4419/96973113.

37. Caria, S. *et al. Child Care Subsidies, Employment Services and Women's Labor Market Outcomes in Egypt: First Midline Results*. https://openknowledge.worldbank.org/handle/10986/37825 (2022).

38. Carneiro, P., Rasul, I., Mason, G. & Kraftman, L. *Child Development Grant Programme Evaluation*. (2019).

39. Christian, P. *et al. El Salvador, Cash-Based Transfers on Food Security and Gender Equality: Impact Evaluation*. https://www.wfp.org/publications/el-salvador-cash-based-transfers-food-security-and-gender-equality-impact-evaluation (2023).

40. Clark, S., Kabiru, C. W., Laszlo, S. & Muthuri, S. The Impact of Childcare on Poor Urban Women's Economic Empowerment in Africa. *Demography* **56**, 1247–1272 (2019).

41. Crépon, B., El Komi, M. & Osman, A. Is It Who You Are or What You Get? Comparing the Impacts of Loans and Grants for Microenterprise Development. *American Economic Journal: Applied Economics* (2023) doi:10.1257/app.20210683.

42. Daidone, S., Davis, B., Dewbre, J. & Covarrubias, K. *Lesotho`s Child Grant Programme: 24-Month Impact Report on Productive Activities and Labour Allocation - Lesotho Country Case Study Report*. https://www.fao.org/reduce-rural-poverty/resources/resources-detail/en/c/468329/ (2014).

43. Daidone, S., Davis, B., Handa, S. & Winters, P. The Household and Individual-Level Productive Impacts of Cash Transfer Programs in Sub-Saharan Africa. *American Journal of Agricultural Economics* **101**, 1401–1431 (2019).

44. de Mel, S., McKenzie, D. & Woodruff, C. Are Women More Credit Constrained? Experimental Evidence on Gender and Microenterprise Returns. *American Economic Journal: Applied Economics* **1**, 1–32 (2009).

45. de Mel, S., McKenzie, D. & Woodruff, C. One-Time Transfers of Cash or Capital Have Long-Lasting Effects on Microenterprises in Sri Lanka. *Science* **335**, 962–966 (2012).

46. de Mel, S., McKenzie, D. & Woodruff, C. Business training and female enterprise start-up, growth, and dynamics: Experimental evidence from Sri Lanka. *Journal of Development Economics* **106**, 199–210 (2014).

47. Devoto, F., Galasso, E., Beegle, K. & Brodmann, S. *Women at Work : Evidence from a Randomized Experiment in Urban Djibouti*. https://documents.worldbank.org/en/publication/documents-reports/documentdetail/099403209162414125/IDU17b26efef1ad3814dcd1b0db17b1018e75b50 (2024).

48. Donald, A. & Vaillant, J. Experimental Evidence on Rural Childcare Provision. Working Paper (2023).

49. Edmonds, E. & Theoharides, C. The short term impact of a productive asset transfer in families with child labor: Experimental evidence from the Philippines. *Journal of Development Economics* **146**, 102486 (2020).

50. Fafchamps, M., McKenzie, D., Quinn, S. & Woodruff, C. Microenterprise growth and the flypaper effect: Evidence from a randomized experiment in Ghana. *Journal of Development Economics* **106**, 211–226 (2014).

51. Fiala, N., Rose, J., Aryemo, F. & Peters, J. The (Very) Long-Run Impacts of Cash Grants during a Crisis. Preprint at https://g2lm-lic.iza.org/publications/wp/the-very-long-run-impacts-of-cash-grants-during-a-crisis/ (2022).

52. Field, E. M. & Maffioli, E. M. Are Behavioral Change Interventions Needed to Make Cash Transfer Programs Work for Children? Experimental Evidence from Myanmar. Working Paper at https://doi.org/10.3386/w28443 (2021).

53. Gallardo, M. A. J., Kananu, W., Lazicky, C., McManus, J. & Njogu-Ndongwe, F. *Village Enterprise Development Impact Bond Evaluation Findings*. https://www.idinsight.org/publication/village-enterprise-development-impact-bond-evaluation-findings/ (2022).

54. Gazeaud, J., Khan, N., Mvukiyehe, E. & Sterck, O. With or without him? Experimental evidence on cash grants and gender-sensitive trainings in Tunisia. *Journal of Development Economics* **165**, 103169 (2023).

55. Gazeaud, J., Mvukiyehe, E. & Sterck, O. *Public Works and Welfare : A Randomized Control Trial of the Comoros Social Safety Net Project - Endline Report*. https://documents.worldbank.org/pt/publication/documents-reports/documentdetail/753341575624212242/Public-Works-and-Welfare-A-Randomized-Control-Trial-of-the-Comoros-Social-Safety-Net-Project-Endline-Report (2022).

56. Gibbs, A. *et al.* The impacts of combined social and economic empowerment training on intimate partner violence, depression, gender norms and livelihoods among women: an individually randomised controlled trial and qualitative study in Afghanistan. *BMJ Glob Health* **5**, e001946 (2020).

57. Gobin, V. J., Santos, P. & Toth, R. No Longer Trapped? Promoting Entrepreneurship Through Cash Transfers to Ultra-Poor Women in Northern Kenya. *American Journal of Agricultural Economics* **99**, 1362–1383 (2017).

58. Gram, L. *et al.* Do Participatory Learning and Action Women's Groups Alone or Combined with Cash or Food Transfers Expand Women's Agency in Rural Nepal? *The Journal of Development Studies* **55**, 1670–1686 (2019).

59. Green, E. P., Blattman, C., Jamison, J. & Annan, J. Women's entrepreneurship and intimate partner violence: A cluster randomized trial of microenterprise assistance and partner participation in post-conflict Uganda (SSM-D-14-01580R1). *Social Science & Medicine* **133**, 177–188 (2015).

60. Groh, M., Krishnan, N., McKenzie, D. & Vishwanath, T. *Soft Skills or Hard Cash? The Impact of Training and Wage Subsidy Programs on Female Youth Employment in Jordan*. https://openknowledge.worldbank.org/handle/10986/11970 (2012) doi:10.1596/1813-9450-6141.

61. Gupta, P. *et al.* Cash transfers amid shocks: A large, one-time, unconditional cash transfer to refugees in Uganda has multidimensional benefits after 19 months. *World Development* **173**, 106339 (2024).

62. Handa, S., Natali, L., Seidenfeld, D., Tembo, G. & Davis, B. Can unconditional cash transfers raise long-term living standards? Evidence from Zambia. *Journal of Development Economics* **133**, 42–65 (2018).

63. Harris-Fry, H. *et al.* Relative power: Explaining the effects of food and cash transfers on allocative behaviour in rural Nepalese households. *Journal of Development Economics* **154**, 102784 (2022).

64. Haushofer, J., Ringdal, C., Shapiro, J. P. & Wang, X. Y. Income Changes and Intimate Partner Violence: Evidence from Unconditional Cash Transfers in Kenya. Working Paper at https://doi.org/10.3386/w25627 (2019).

65. Haushofer, J. & Shapiro, J. The short-term impact of unconditional cash transfers to the poor: Experimental evidence from Kenya. *The quarterly journal of economics* **131 4**, 1973–2042 (2016).

66. Heath, R., Hidrobo, M. & Roy, S. Cash transfers, polygamy, and intimate partner violence: Experimental evidence from Mali. *Journal of Development Economics* **143**, 102410 (2020).

67. Hidrobo, M. & Fernald, L. Cash transfers and domestic violence. *Journal of health economics* **32 1**, 304–19 (2013).

68. Hidrobo, M., Peterman, A. & Heise, L. The Effect of Cash, Vouchers, and Food Transfers on Intimate Partner Violence: Evidence from a Randomized Experiment in Northern Ecuador. *American Economic Journal: Applied Economics* **8**, 284–303 (2016).

69. Hojman, A. & Lopez Boo, F. Public childcare benefits children and mothers: Evidence from a nationwide experiment in a developing country. *Journal of Public Economics* **212**, 104686 (2022).

70. Hossain, S. J. *et al.* Effects of integrated psychosocial stimulation (PS) and Unconditional Cash Transfer (UCT) on Children's development in rural Bangladesh: A cluster randomized controlled trial. *Social Science & Medicine* **293**, 114657 (2022).

71. Hussam, R., Kelley, E. M., Lane, G. & Zahra, F. The Psychosocial Value of Employment: Evidence from a Refugee Camp. *American Economic Review* **112**, 3694–3724 (2022).

72. Ismayilova, L. & Karimli, L. Harsh Parenting and Violence Against Children: A Trial with Ultrapoor Families in Francophone West Africa. *Journal of Clinical Child & Adolescent Psychology* **49**, 18–35 (2020).

73. Janzen, S., Magnan, N., Sharma, S. & Thompson, W. *Pay It Forward: A Mechanism for Achieving Scale in Anti-Poverty Programs*. (2023).

74. Karasz, A., Anne, S., Hamadani, J. D. & Tofail, F. The ASHA (Hope) Project: Testing an Integrated Depression Treatment and Economic Strengthening Intervention in Rural Bangladesh: A Pilot Randomized Controlled Trial. *International Journal of Environmental Research and Public Health* **18**, 279 (2021).

75. Karimli, L., Bose, B. & Kagotho, N. Integrated Graduation Program and its Effect on Women and Household Economic Well-being: Findings from a Randomised Controlled Trial in Burkina Faso. *The Journal of Development Studies* **56**, 1277–1294 (2020).

76. Kashefi, F. & Naito, H. Does receiving a cash grant improve individual earnings in a war-torn country? Evidence from a randomized experiment in Afghanistan. Preprint at https://doi.org/10.12688/f1000research.72893.2 (2023).

77. Kilburn, K. *et al.* Cash Transfers, Young Women's Economic Well-Being, and HIV Risk: Evidence from HPTN 068. *AIDS Behav* **23**, 1178–1194 (2019).

78. Kilburn, K. *et al.* Conditional cash transfers and the reduction in partner violence for young women: an investigation of causal pathways using evidence from a

randomized experiment in South Africa (HPTN 068). *Journal of the International AIDS Society* **21**, e25043 (2018).

79. Kuringe, E. *et al.* Effectiveness of Cash Transfer Delivered Along With Combination HIV Prevention Interventions in Reducing the Risky Sexual Behavior of Adolescent Girls and Young Women in Tanzania: Cluster Randomized Controlled Trial. *JMIR Public Health and Surveillance* **8**, null (2022).

80. Lambon-Quayefio, M. *et al.* Unconditional cash transfers and safe transitions to adulthood in Malawi. *World Development* **175**, 106483 (2024).

81. Leight, J. & Mvukiyehe, E. *Short-Term and Long-Term Effects of Cash for Work: Evidence from a Randomized Controlled Trial in Tunisia*. (Intl Food Policy Res Inst, 2023).

82. Macours, K. & Vakis, R. Changing Households' Investment Behaviour through Social Interactions with Local Leaders: Evidence from a Randomised Transfer Programme. *The Economic Journal* **124**, 607–633 (2014).

83. Martínez, C. & Perticará, M. Childcare effects on maternal employment: Evidence from Chile. *Journal of Development Economics* **126**, 127–137 (2017).

84. Molina Millán, T., Macours, K., John A., M. & Tejerina, L. *The Long-Term Impacts of Honduras' CCT Program: Higher Education and International Migration*. https://publications.iadb.org/en/long-term-impacts-honduras-cct-program-higher-education-and-international-migration (2019) doi:10.18235/0001670.

85. Nandi, A., Agarwal, P., Chandrashekar, A. & Harper, S. Access to affordable daycare and women's economic opportunities: evidence from a cluster-randomised intervention in India. *Journal of Development Effectiveness* **12**, 219–239 (2020).

86. Olney, D. K. *et al.* A 2-Year Integrated Agriculture and Nutrition Program Targeted to Mothers of Young Children in Burkina Faso Reduces Underweight among Mothers and Increases Their Empowerment: A Cluster-Randomized Controlled Trial123. *The Journal of Nutrition* **146**, 1109–1117 (2016).

87. Orkin, K. *et al.* Aspiring to a Better Future: How a Simple Psychological Intervention Increases Investment. Preprint at https://www.dropbox.com/s/u17dl4dbpx5bp4p/Aspiring_to_a_better_future_rev.pdf?dl=0 (2023).

88. Pace, N., Daidone, S., Davis, B. & Pellerano, L. Shaping Cash Transfer Impacts Through 'Soft-Conditions': Evidence from Lesotho†. *Journal of African Economies* **28**, 39–69 (2019).

89. Palermo, T., Prencipe, L. & Kajula, L. Effects of Government-Implemented Cash Plus Model on Violence Experiences and Perpetration Among Adolescents in Tanzania, 2018–2019. *Am J Public Health* **111**, 2227–2238 (2021).

90. Perova, E., Johnson, E., Mannava, A., Reynolds, S. & Teman, A. *Public Work Programs and Gender-Based Violence: Evidence from Lao PDR*. https://openknowledge.worldbank.org/handle/10986/35728 (2021) doi:10.1596/1813-9450-9691.

91. Peterman, A., Schwab, B., Roy, S., Hidrobo, M. & Gilligan, D. O. Measuring women's decisionmaking: Indicator choice and survey design experiments from cash and food transfer evaluations in Ecuador, Uganda and Yemen. *World Development* **141**, 105387 (2021).

92. Rahman, A., Bhattacharjee, A. & Das, N. A good mix against ultra-poverty? Evidence from a Randomized Controlled Trial (RCT) in Bangladesh. *Review of Development Economics* **25**, 2052–2083 (2021).

93. Ranganathan, M. *et al.* Government of Ethiopia's public works and complementary programmes: A mixed-methods study on pathways to reduce intimate partner violence. *Social Science & Medicine* **294**, 114708 (2022).

94. Rosas, N. & Sabarwal, S. *Can You Work It? Evidence on the Productive Potential of Public Works from a Youth Employment Program in Sierra Leone*. https://papers.ssrn.com/sol3/papers.cfm?abstract_id=2849107 (2016).

95. Rosas, N., Acevedo, M. C. & Zaldivar, S. Starting points matter: Cash plus training effects on youth entrepreneurship, skills, and resilience during an epidemic. *World Development* **149**, 105698 (2022).

96. Roy, S., Ara, J., Das, N. & Quisumbing, A. R. "Flypaper effects" in transfers targeted to women: Evidence from BRAC's "Targeting the Ultra Poor" program in Bangladesh. *Journal of Development Economics* **117**, 1–19 (2015).

97. Roy, S., Hidrobo, M., Hoddinott, J. & Ahmed, A. Transfers, Behavior Change Communication, and Intimate Partner Violence: Postprogram Evidence from Rural Bangladesh. *The Review of Economics and Statistics* **101**, 865–877 (2019).

98. Roy, S., Hidrobo, M., Hoddinott, J., Koch, B. & Ahmed, A. Can transfers and complementary nutrition programming reduce intimate partner violence four years post-program? Experimental evidence from Bangladesh. *Journal of Human Resources* (2024) doi:10.3368/jhr.0720-11014R2.

99. Tanzania Social Action Fund, UNICEF Office of Research, Innocenti & Policy Research for Development (REPOA). *Tanzania Youth Study of the Productive Social Safety Net (PSSN) Evaluation: Endline Report*. https://www.unicef-irc.org/publications/942-.html (2018).

100. Urbina, D. R. In the Hands of Women: Conditional Cash Transfers and Household Dynamics. *Journal of Marriage and Family* **82**, 1571–1586 (2020).

101. van den Bold, M. *et al.* Can Integrated Agriculture-Nutrition Programmes Change Gender Norms on Land and Asset Ownership? Evidence from Burkina Faso. *The Journal of Development Studies* **51**, 1155–1174 (2015).

102. Wolf, S., Kembou, S., Ogan, A. & Jasinska, K. Cash Transfers Improve Economic Conditions and Reduce Maternal Stress in Rural Côte d'Ivoire. *J Child Fam Stud* **33**, 1251–1265 (2024).
