# Supplementary material for: Social safety nets, women’s economic achievements and agency in 45 countries: a systematic review and meta-analysis
Source: Nat Hum Behav. 2026 Feb 5;10(4):698–714. doi: 10.1038/s41562-025-02394-0 (PMC13121000; doi:10.1038/s41562-025-02394-0)
Supplement: Supplementary file 2 — Reporting Summary [file 41562_2025_2394_MOESM2_ESM.pdf]

nature portfolio

Corresponding author(s): Janina Steinert
Last updated by author(s): Jul 30, 2025

Reporting Summary

Nature Portfolio wishes to improve the reproducibility of the work that we publish. This form provides structure for consistency and transparency in reporting. For further information on Nature Portfolio policies, see our Editorial Policies and the Editorial Policy Checklist.

Statistics

For all statistical analyses, confirm that the following items are present in the figure legend, table legend, main text, or Methods section.

n/a	Confirmed	
☐	☒	The exact sample size (*n*) for each experimental group/condition, given as a discrete number and unit of measurement
☒	☐	A statement on whether measurements were taken from distinct samples or whether the same sample was measured repeatedly
☐	☒	The statistical test(s) used AND whether they are one- or two-sided<br>*Only common tests should be described solely by name; describe more complex techniques in the Methods section.*
☐	☒	A description of all covariates tested
☐	☒	A description of any assumptions or corrections, such as tests of normality and adjustment for multiple comparisons
☐	☒	A full description of the statistical parameters including central tendency (e.g. means) or other basic estimates (e.g. regression coefficient) AND variation (e.g. standard deviation) or associated estimates of uncertainty (e.g. confidence intervals)
☐	☒	For null hypothesis testing, the test statistic (e.g. *F*, *t*, *r*) with confidence intervals, effect sizes, degrees of freedom and *P* value noted<br>*Give P values as exact values whenever suitable.*
☒	☐	For Bayesian analysis, information on the choice of priors and Markov chain Monte Carlo settings
☐	☒	For hierarchical and complex designs, identification of the appropriate level for tests and full reporting of outcomes
☐	☒	Estimates of effect sizes (e.g. Cohen's *d*, Pearson's *r*), indicating how they were calculated

Our web collection on statistics for biologists contains articles on many of the points above.

Software and code

Policy information about availability of computer code

Data collection	Eligible articles and effects were identified through bibliometric and research repository database searches, title and abstract screening using Covidence software and subsequent full text screening. Futher description of search and screening protocols, including search strings in English, French and Spanish are included in Supplementary Material.
Data analysis	Descriptive analysis of key sample parameters was conducted in Stata 15.0 and meta-analysis was conducted in R 4.3.1, including packages "robumeta," "metafor" and "orchaRd." Replication materials are available on the OSF repository: https://osf.io/brvy2

For manuscripts utilizing custom algorithms or software that are central to the research but not yet described in published literature, software must be made available to editors and reviewers. We strongly encourage code deposition in a community repository (e.g. GitHub). See the Nature Portfolio guidelines for submitting code & software for further information.

Data

Policy information about availability of data

All manuscripts must include a data availability statement. This statement should provide the following information, where applicable:

- Accession codes, unique identifiers, or web links for publicly available datasets
- A description of any restrictions on data availability
- For clinical datasets or third party data, please ensure that the statement adheres to our policy

Data availability: Data are available on the Center for Open Science public repository via the following link: https://osf.io/brvy2. The data extraction form is not

publicly available.
Code availability: All replication files and code used for the analysis are available at: https://osf.io/brvy2/.

Research involving human participants, their data, or biological material

Policy information about studies with human participants or human data. See also policy information about sex, gender (identity/presentation), and sexual orientation and race, ethnicity and racism.

Reporting on sex and gender	N/A
Reporting on race, ethnicity, or other socially relevant groupings	N/A
Population characteristics	N/A
Recruitment	N/A
Ethics oversight	N/A

Note that full information on the approval of the study protocol must also be provided in the manuscript.

Field-specific reporting

Please select the one below that is the best fit for your research. If you are not sure, read the appropriate sections before making your selection.

☐ Life sciences ☒ Behavioural & social sciences ☐ Ecological, evolutionary & environmental sciences

For a reference copy of the document with all sections, see nature.com/documents/nr-reporting-summary-flat.pdf

Behavioural & social sciences study design

All studies must disclose on these points even when the disclosure is negative.

Study description	We conduct a systematic review and meta-analysis of quantitative experimental studies examining the impacts of social safety nets on women's economic achievement and agency outcomes. Our key research questions, study design, search protocol and analysis plan was pre-registered with PROSPERO: https://www.crd.york.ac.uk/prospero/display_record.php?RecordID=382158
Research sample	The final sample for the analysis originated from 5,120 paper hits (4,146 from databases and 974 from other sources) and includes 1,307 effect sizes from 115 papers, representing 93 studies and 218,828 women. Included papers primarily interviewed women (aged 18 and over), however a minority of studies included male respondents reporting on women's outcomes. The sample was constructed according to standardized guidelines for systematic review with criteria developed with respect to eight parameters: intervention, setting, population, outcomes, methodology, time frame, type of publication and language. Individual papers varied in their data collection strategies, however typically include household-level survey data collection. The samples included in this review are largely not population representative, as they are meant to represent eligible populations for particular social safety net interventions.
Sampling strategy	Eligible papers, studies and effects were identified primarily through database searches, following standardized guidelines for systematic reviews. We did not have a formal sample size determination method for the pooled analysis or the sub-sample analysis conducted. Rather the sample size was determined by the number of eligible studies and qualifying effect sizes. However, meta-analyses and -regressions were only performed if the degrees of freedom (df) were greater than 4, as simulations have shown that the approximation of the variance distribution is not valid if df<4.
Data collection	Information from each qualifying study was extracted using excel by one study author and subsequently checked by a second study author. As the study is a review, there was no rationale to blind researchers to experimental condition and/or the study hypothesis.
Timing	Journal publications and grey literature released starting January 1, 2003 - December 1, 2024 which met other inclusion criteria were included.
Data exclusions	Of an original potential sample of 5,120 paper hits (4,146 from databases and 974 from other sources) all except 115 papers were excluded, primarily because they measured the wrong outcome, included the wrong population, did not study impact of a social safety net or did not conduct an experimental impact evaluation. Additional details on exclusions are included in Fig. 1.
Non-participation	N/A - no participants were involved, as this is a review study.
Randomization	N/A - no primary randomization was conducted, as this is a review study.

Reporting for specific materials, systems and methods

We require information from authors about some types of materials, experimental systems and methods used in many studies. Here, indicate whether each material, system or method listed is relevant to your study. If you are not sure if a list item applies to your research, read the appropriate section before selecting a response.

Materials & experimental systems

n/a	Involved in the study
☒	☐ Antibodies
☒	☐ Eukaryotic cell lines
☒	☐ Palaeontology and archaeology
☒	☐ Animals and other organisms
☒	☐ Clinical data
☒	☐ Dual use research of concern
☒	☐ Plants

Methods

n/a	Involved in the study
☒	☐ ChIP-seq
☒	☐ Flow cytometry
☒	☐ MRI-based neuroimaging

Plants

Seed stocks	N/A
Novel plant genotypes	N/A
Authentication	N/A
